# A methylation platform of unconventional inert aryl electrophiles: trimethylboroxine as a universal methylating reagent†

**DOI:** 10.1039/d0sc01641a

**Published:** 2020-05-25

**Authors:** Boya Feng, Yudong Yang, Jingsong You

**Affiliations:** Key Laboratory of Green Chemistry and Technology of Ministry of Education, College of Chemistry, Sichuan University 29 Wangjiang Road Chengdu 610064 P. R. China jsyou@scu.edu.cn yangyudong@scu.edu.cn

## Abstract

Methylation is one of the most fundamental conversions in medicinal and material chemistry. Extension of substrate types from aromatic halides to other unconventional aromatic electrophiles is a highly important yet challenging task in catalytic methylation. Disclosed herein is a series of transition metal-catalyzed methylations of unconventional inert aryl electrophiles using trimethylboroxine (TMB) as the methylating reagent. This transformation features a broad substrate type, including nitroarenes, benzoic amides, benzoic esters, aryl cyanides, phenol ethers, aryl pivalates and aryl fluorides. Another important merit of this work is that these widespread “inert” functionalities are capable of serving as directing or activating groups for selective functionalization of aromatic rings before methylation, which greatly expands the connotation of methylation chemistry.

## Abstract 2

A universal platform for catalytic methylation of a series of unconventional inert aryl electrophiles with trimethylboroxine (TMB) is established, which provides a synthetic chemist's toolbox for the construction of methylated arenes.

## Graphical abstract

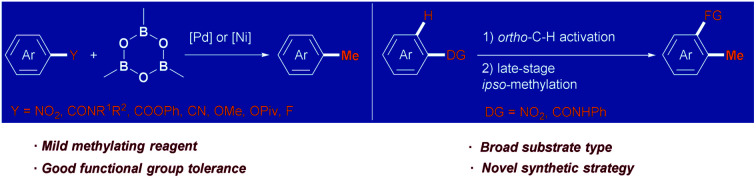

## Introduction

**Scheme 1:**
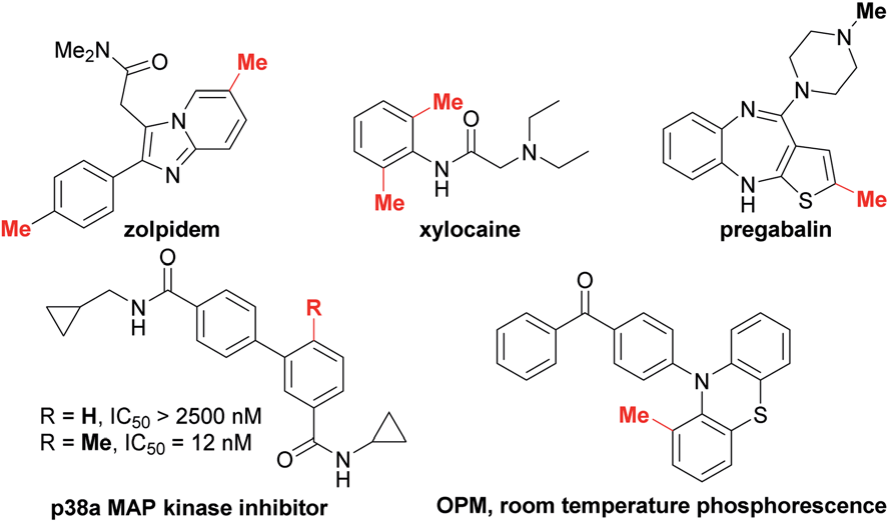
Examples of drugs and materials containing C(aryl)–Me bond(s).

Methyl groups are ubiquitous in biological molecules, pharmaceuticals and organic functional materials (Scheme 1).^1^ The incorporation of a methyl group may dramatically alter both the physical properties and biological activity of molecules, including solubility, hydrophilicity, half-life and conformation of drugs. For example, *ortho*-methylation significantly improves the potency (208-fold increase) of the p38α MAP kinase inhibitor.^2^ Nowadays, methylation has been widely used for modifying biomolecules in drug discovery owing to the well-known “magic methyl effect”.^3^ It is reported that more than 73% of the top 200 selling small-molecule drugs in 2018 contain at least one methyl group.^1*e*^ In addition, the methyl group is often used to optimize the performance of organic optoelectronic materials *via* regulating the molecular packing, planarity and exciton behaviors.^1*c*,*d*,4^

**Scheme 2:**
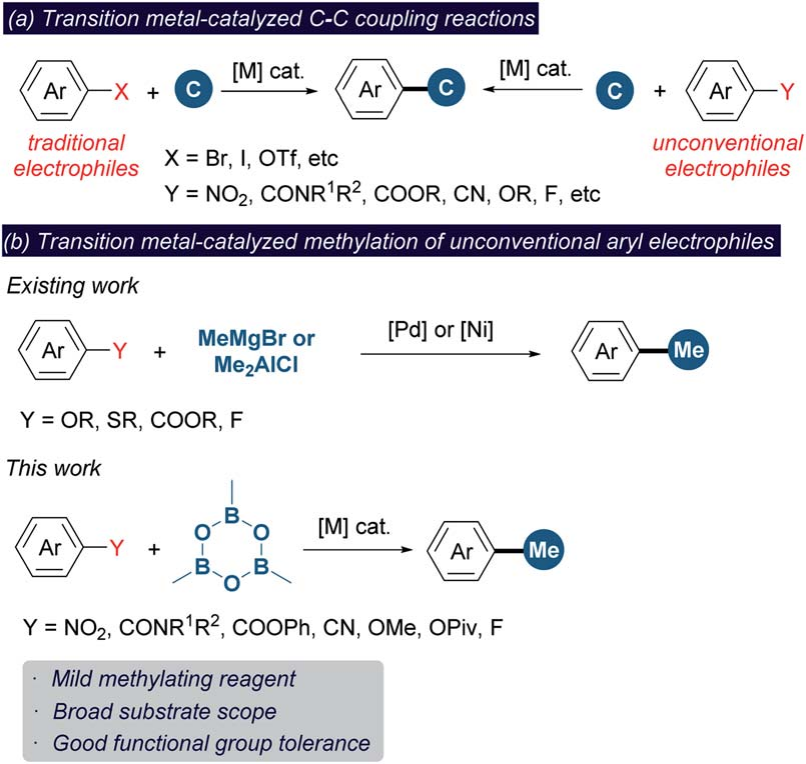
Transition metal-catalyzed methylation of unconventional aryl electrophiles.

Transition metal-catalyzed cross-coupling reaction between an aryl electrophile and a nucleophilic reagent represents a powerful synthetic tool for the construction of the C–C bond (Scheme 2a).^5^ Conventionally, these reactions mainly rely on aryl halides as the electrophilic coupling partner. Recently, the cross-coupling reactions using alternative aryl electrophiles, such as benzamides,^6^ benzoic esters,^6*a*,*b*,7^ nitro arenes,^8^ anisoles,^9^ aryl pivalates,^9*a*,10^ aryl fluorides^11^ and aryl cyanides,^12^ have emerged as a fascinating and challenging topic. Given that these aryl electrophiles are abundant and easily available, using them as surrogates instead of aryl halides would greatly expand the connotation of methylation chemistry. However, unlike aryl halides, implementing the methylation of unconventional aryl electrophiles may suffer from the following two obstacles: (1) a large energy barrier in the oxidative addition of inert chemical bonds to the metal center render this step sluggish to take place. (2) The oxidative addition is typically considered as the rate-determining step in these reactions.^7*c*,8*a*,13^ The methylating reagent may decompose during this process owing to unmatched reaction rates between oxidative addition and other elementary reactions such as transmetalation.^14^ Despite progress in this line, only a limited number of unconventional functionalities have been documented to undergo methylation (Scheme 2b).^7*d*,9*b*,9*c*^ These existing methods typically require highly reactive organometallic reagents (MeMgBr and Me_2_AlCl) as the methyl sources, which can enhance the electrophilicity by coordination but weaken the functional group tolerance. In addition, these reagents are readily hydrolyzed to unreactive “Me” species in the presence of water. Thus, developing the methylation chemistry of unconventional aryl electrophiles that is able to overcome the above restrictions is highly desired yet challenging. Herein we wish to disclose a methylation platform of unconventional inert aryl electrophiles with excellent functional group tolerance using trimethylboroxine (TMB) as the methyl source that is a cheaper and a fairly soluble anhydride alternative of methylboronic acid.^3*d*,15^

## Results and discussion

Nitroarenes are widespread and easily prepared by the nitration of aromatic rings. Thus, 1-nitronaphthalene was selected as the initially investigated substrate (Table 1). In the presence of Pd(acac)_2_ (5 mol%), screening of ligands indicated that BrettPhos was the most optimum, affording 1-methylnaphthalene in 80% yield (entries 1–5). The switch of the solvent and base as well as the reducing temperature led to diminished yields (entries 6–9). Other palladium catalysts such as Pd(OAc)_2_ and Pd_2_(dba)_3_ showed inferior efficiency (entries 10 and 11). Other common nucleophilic methylating reagents such as ZnMe_2_, MeMgBr and DABAL-Me_3_ proved to be ineffective (entries 12–14). The use of MeB(OH)_2_ gave the desired product in 55% yield along with the hydrodenitrated naphthalene (entry 15).

**Table 1:** Optimization of reaction conditions^a^

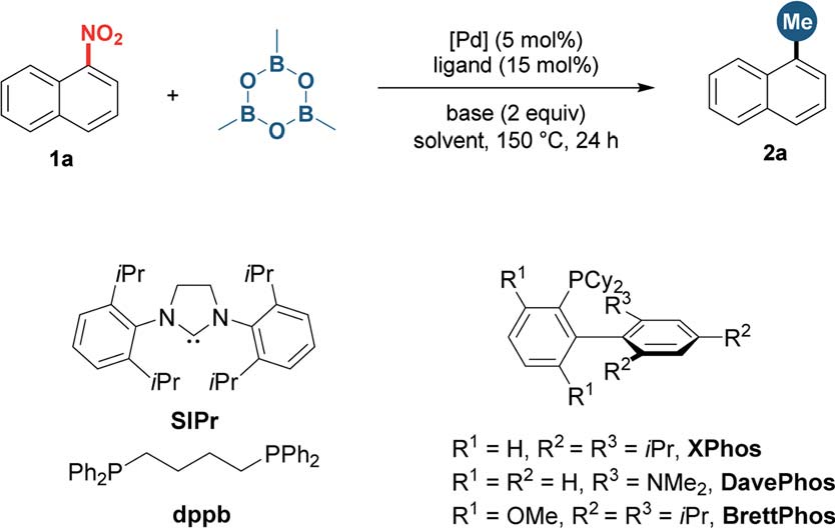					
Entry	“Me” reagent	Catalyst	Ligand	Base	Yield^b^ (%)
1	TMB	Pd(acac)_2_	SIPr	Cs_2_CO_3_	n.d.
2	TMB	Pd(acac)_2_	dppb	Cs_2_CO_3_	n.d.
3	TMB	Pd(acac)_2_	DavePhos	Cs_2_CO_3_	n.d.
4	TMB	Pd(acac)_2_	XPhos	Cs_2_CO_3_	18
5	TMB	Pd(acac)_2_	BrettPhos	Cs_2_CO_3_	80
6^c^	TMB	Pd(acac)_2_	BrettPhos	Cs_2_CO_3_	43
7^d^	TMB	Pd(acac)_2_	BrettPhos	Cs_2_CO_3_	53
8	TMB	Pd(acac)_2_	BrettPhos	K_3_PO_4_	25
9	TMB	Pd(acac)_2_	BrettPhos	CsF	32
10	TMB	Pd(OAc)_2_	BrettPhos	Cs_2_CO_3_	54
11	TMB	Pd_2_(dba)_3_	BrettPhos	Cs_2_CO_3_	50
12	ZnMe_2_	Pd(acac)_2_	BrettPhos	Cs_2_CO_3_	n.d.
13	MeMgBr	Pd(acac)_2_	BrettPhos	Cs_2_CO_3_	n.d.
14	DIBAL-Me_3_	Pd(acac)_2_	BrettPhos	Cs_2_CO_3_	n.d.
15^e^	MeB(OH)_2_	Pd(acac)_2_	BrettPhos	Cs_2_CO_3_	55
16	TMB	—	BrettPhos	Cs_2_CO_3_	n.d.

a Reaction conditions: **1a** (0.2 mmol, 1 equiv.), “Me” reagent (1.75 equiv.), catalyst (5 mol%), ligand (15 mol%) and base (2 equiv.) in toluene (0.6 mL) at 150 °C for 24 h under N_2_.

b Isolated yields.

c 1,4-Dioxane as the solvent.

d 130 °C.

e 5 equiv. of MeB(OH)_2_. DABAL-Me_3_ = bis(trimethylaluminum)-1,4-diazabicyclo[2.2.2]octane adduct.

With the optimal conditions in hand, we then examined the substrate scope. As shown in Table 2, α- and β-nitro naphthalenes were methylated in 80% and 68% yields, respectively (**2a** and **2b**). A set of nitrobenzenes with electronically and sterically different substituents could smoothly undergo methylation (**2c–2j**). Notably, functional groups such as ketone, morpholine, methoxy and fluoro groups were able to survive from this methylation reaction. A number of NO_2_-substituted condensed (hetero)aromatics, including 9-phenanthrenyl, 1-pyrenyl, 3-perylenyl and 5-quinolinyl derivatives, proved to be suitable substrates (**2k–2n**). To our delight, more complex natural product derivatives such as chromane and estrone were tolerated in this reaction, demonstrating the potential of this method in late-stage methylation (**2o** and **2p**).

**Table 2:** Scope of nitroarenes^a^

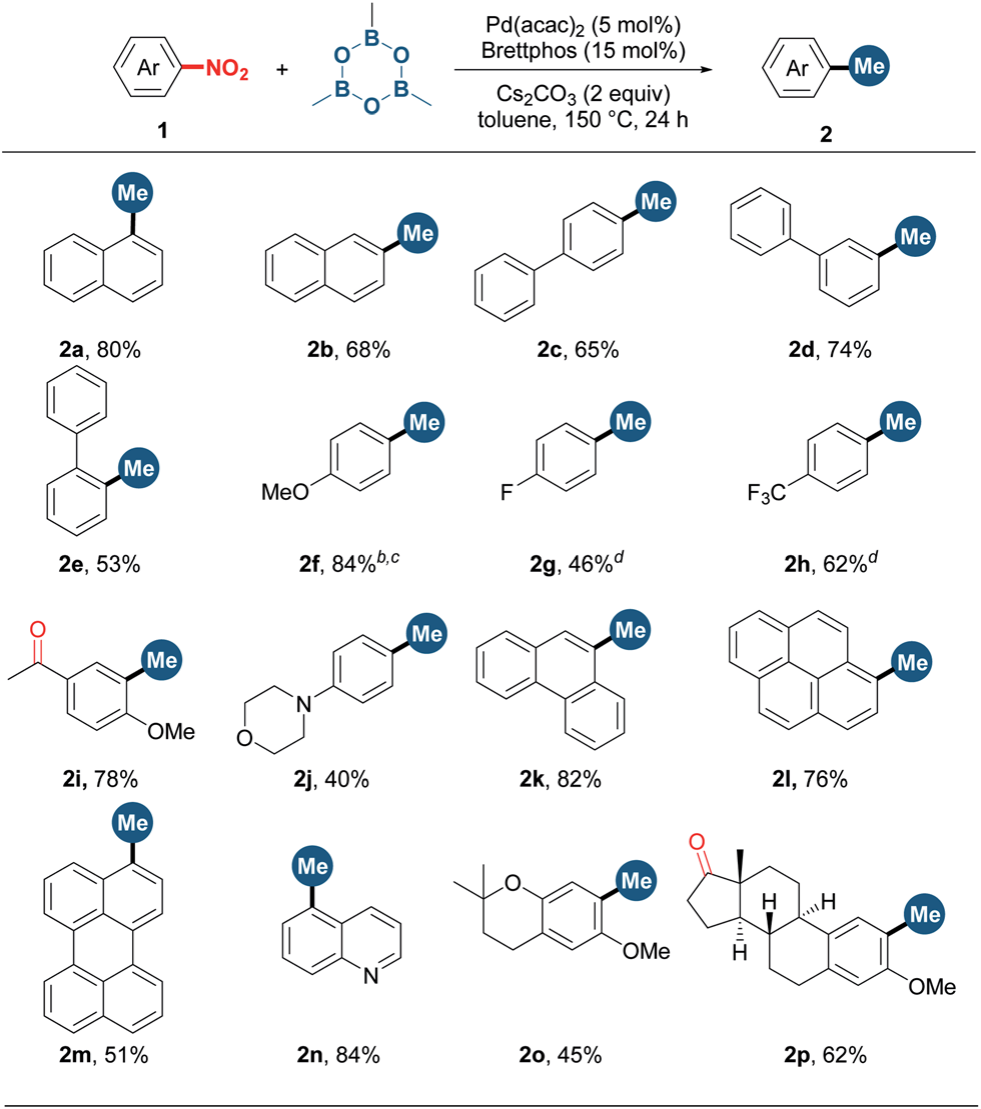

a Reaction conditions: **1** (0.2 mmol, 1 equiv.), TMB (1.75 equiv.), Pd(acac)_2_ (5 mol%), Brettphos (15 mol%) and Cs_2_CO_3_ (2 equiv.) in toluene (0.6 mL) at 150 °C for 24 h under N_2_. Isolated yields.

b 130 °C.

c GC yield (internal standard: diphenylacetylene).

d GC yield (internal standard: mesitylene).

Encouraged by the success of the denitrative methylation reaction, we next focused on aromatic amide and carboxylic ester derivatives. However, the initial attempts of the decarbonylative methylation of NH-free aromatic amides were disappointing. Inspired by Szostak's work,^6*c*,*f*^ we envisioned that the installation of a sterically demanding group on the amide would lead to a twisted N–C(O) bond and thus facilitate decarbonylative methylation. Screening of the substituents of the amide indicated that tosyl was the most suitable group (Table S1†). It is worthy of note that *N*-phenyl-*N*-tosylbenzamides are easily synthesized by the sulfonylation of benzamides with tosyl chloride. The optimized catalytic system consisted of [Pd(allyl)Cl]_2_/dppb/CsF/1,4-dioxane (Table S2†). The benzamides with substituents at *o*-, *m*- and *p*-positions all worked well in moderate to high yields (Table 3, **2a–2h** and **2q**). Heteroaryl substrates such as benzothiophene- and benzofuran-2-carboxamide derivatives also underwent decarbonylative methylation (**2r** and **2s**). In the reactions of **3f**, **3g** and **3h**, the decomposition of starting materials was observed with substrate recoveries of 38%, 21% and 0%, respectively.

**Table 3:** Scope of the benzoic acid derivatives^a^

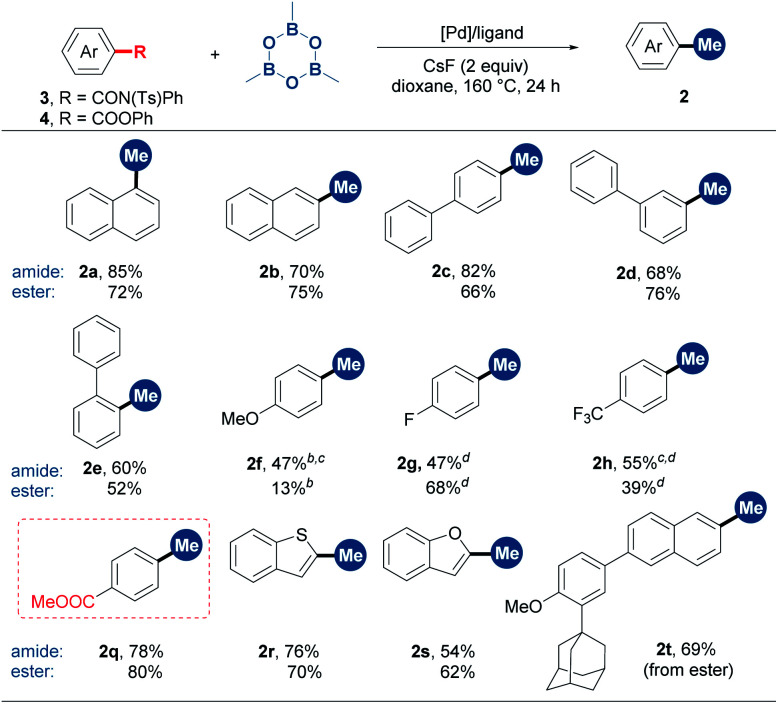

a Reaction conditions: **3** or **4** (0.2 mmol, 1 equiv.), TMB (1.75 equiv.), a palladium catalyst, a phosphine ligand and CsF (2 equiv.) in dioxane (0.6 mL) at 160 °C for 24 h under N_2_. Isolated yields. For amides, [Pd(allyl)Cl]_2_ (5 mol%) and dppb (20 mol%) were used. For esters, Pd(OAc)_2_ (10 mol%) and dcype (20 mol%) were used.

b GC yield (internal standard: diphenylacetylene).

c Et_3_N (2 equiv.) was added.

d GC yield (internal standard: mesitylene). dcype = 1,2-bis(dicyclohexylphosphino)ethane.

Although benzoic esters remained intact in the [Pd(allyl)Cl]_2_/dppb catalytic system, switching the ligand to dcype could lead to the decarbonylative cross-coupling reaction between the aromatic ester and TMB (**2a–2h**, **2r** and **2s**). The reaction condition optimization showed that 5 mol% of Pd(OAc)_2_ was enough for the decarbonylative methylation of ester **4a** (Table S3, entry 4†). However, diminished yields were observed in some cases in the substrate scope examination in this catalyst loading (5 mol%). Esters **4f** and **4h** showed poor reactivities with substrate recoveries of 59% and 0%, respectively, because of the substrate decomposition. Very interestingly, when an arene containing both the methyl ester and the phenyl ester was subjected to the reaction, only the phenyl ester fragment was cleaved, providing an opportunity for chemoselective modification (**2q**). The phenyl ester derivative of Adapalene, a medicine used for treatment of acne,^16^ was methylated successfully (**2t**). Notably, TMB exhibited significantly higher efficiency than MeB(OH)_2_. For example, the reaction with MeB(OH)_2_ led to a diminished yield (for aromatic amide **3a**: 55% yield) or only a trace yield (for carboxylic ester **4a**) (Table S2 and S3†). In addition, when the reaction was conducted at 60 °C using tricyclohexyl phosphine as the ligand instead, non-decarbonylative coupling took place, delivering a methyl aryl ketone *via* the cleavage of (O)C–N and (O)C–O bonds, respectively (Table S4, entry 3 and Table S5, entry 3†).

Next, a series of competition experiments between electronically different substrates were conducted (Scheme S4†). The results suggested that the aryl rings bearing electron-withdrawing groups were more favorable for methylation.

**Scheme 3:**
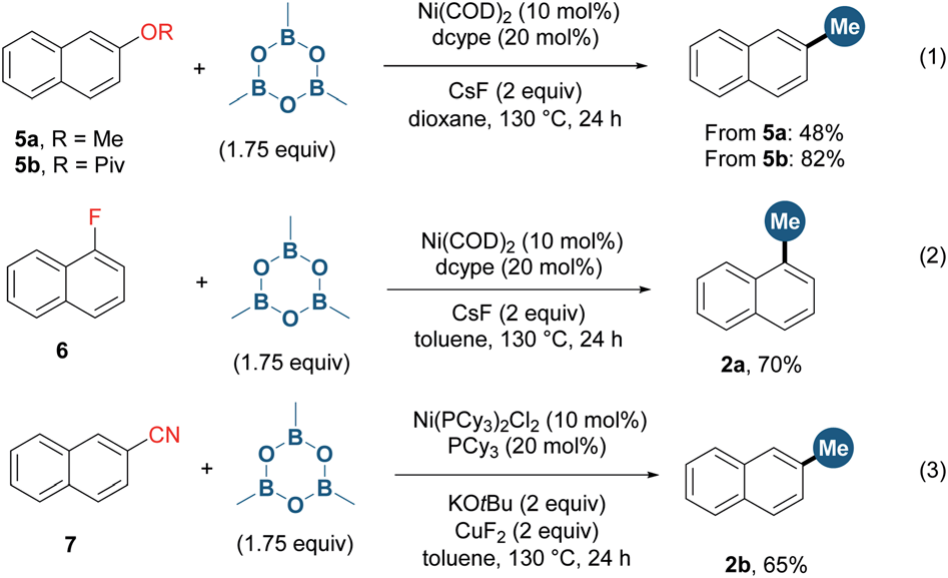
Extension of the substrate types to other inert aryl electrophiles.

To further demonstrate the generality of TMB in the methylation of unconventional aryl electrophiles, the reactions of aryl methyl ether, pivalate, fluoride and cyanide with TMB were performed through nickel catalysis,^9*b*,12*b*^ obtaining the desired products in moderate to high yields (Scheme 3).

**Scheme 4:**
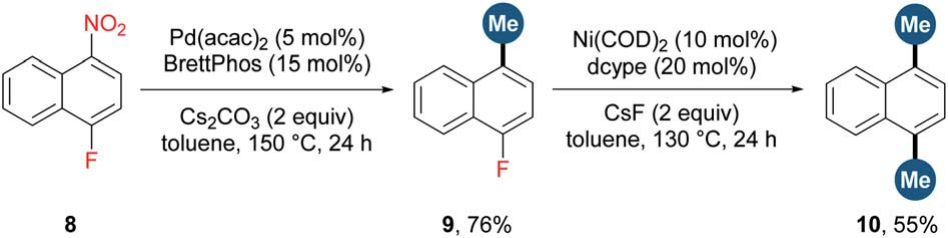
Sequential methylation of 1-fluoro-4-nitronaphthalene.

**Scheme 5:**
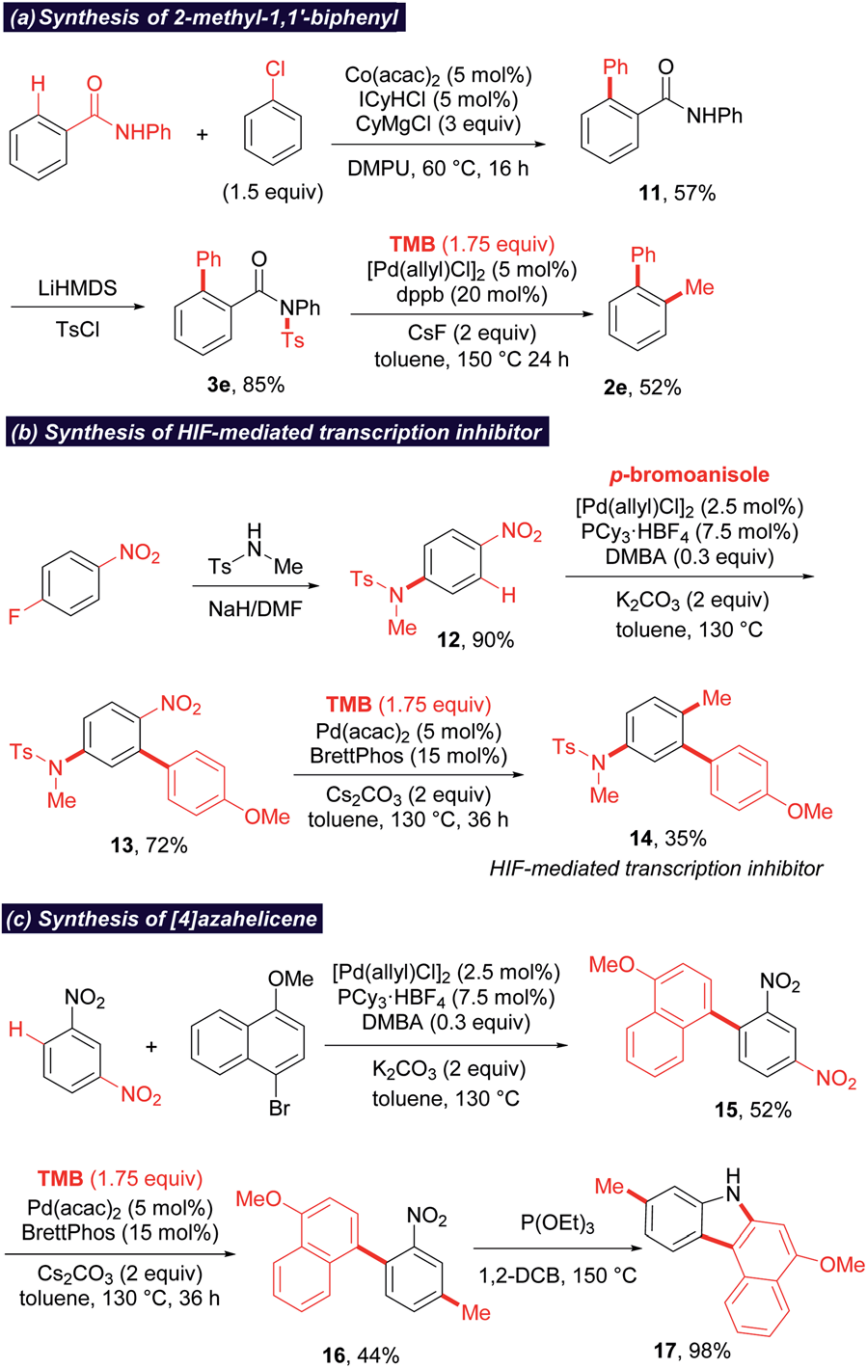
Synthetic applications. ICyHCl = 1,3-dicyclohexyl-imidazolium chloride; DMPU = *N*,*N*-dimethyl propionyl urea; DMBA = 2,2-dimethylbutanoic acid; 1,2-DCB = 1,2-dichlorobenzene.

Catalytic methylation *via* the cleavage of the relatively “inert” chemical bonds would facilitate orthogonal cross-coupling and late-stage methylation. As illustrated in Scheme 4, C–NO_2_ and C–F could be chemoselectively cleaved and methylated, enabling the sequential methylation of 1-fluoro-4-nitronaphthalene at different stages. Moreover, widespread functionalities such as amide and nitro enable a directing or activating group for the regioselective functionalizations of aromatic rings. The combination of these functionalizations with methylation can provide a useful toolbox for the preparation of methylated arenes. To illustrate the potential application of this protocol, three examples are displayed in Scheme 5. (1) A 2-methyl biaryl structure could be easily constructed by the sequential cobalt-catalyzed *ortho*-C–H arylation^17^/sulfonylation/*ipso*-decarbonylative methylation of aromatic amide (Scheme 5a). (2) The strong electron-withdrawing effect of the nitro group facilitates *ortho*-selective C–H arylation.^18^ Thus, an HIF-mediated transcription inhibitor (**14**)^19^ was successfully synthesized by sequential C–F amination/C–H arylation/denitrative methylation starting from *p*-fluoronitrobenzene (Scheme 5b). (3) The 7*H*-benzo[*c*]carbazole derivative (**17**), a scaffold in organic electric devices,^20^ could be synthesized concisely *via* a C–H arylation/denitrative methylation/Cadogan reaction sequence starting from 1,3-dinitrobenzene (Scheme 5c). Notably, the denitrative methylation occurred regioselectively at the less hindered position when two nitro groups exist.

## Conclusions

In conclusion, a palladium- or nickel-catalyzed methylation of unconventional aryl electrophiles with trimethylboroxine has been established. A series of inert chemical bonds including C–NO_2_, C–CON(Ts)Ph, C–COOPh, C–OPiv, C–OMe, C–F and C–CN are cleaved and *ipso*-methylated. TMB is demonstrated as a universal and preferable methylating reagent in these reactions. The present findings not only overcome the limitations on the substrate types in catalytic methylation but also promote the development of inert chemical bond activation. This protocol would offer a powerful platform for the construction of C(aryl)–Me in drug and new material discovery.

## Conflicts of interest

There are no conflicts to declare.

## Supplementary Material

SC-011-D0SC01641A-s001
