# Supplementary material for: A methylation platform of unconventional inert aryl electrophiles: trimethylboroxine as a universal methylating reagent
Source: Chem Sci. 2020 May 25;11(23):6031–5. doi: 10.1039/d0sc01641a (PMC8159405; doi:10.1039/d0sc01641a)

Electronic Supplementary Material (ESI) for Chemical Science.
This journal is © The Royal Society of Chemistry 2020

Supplementary Information

Methylation Platform of Unconventional Inert Aryl Electrophiles: Trimethylboroxine as a Universal Methylating Reagent

Boya Feng, Yudong Yang and Jingsong You**

Key Laboratory of Green Chemistry and Technology of Ministry of Education, College of Chemistry, Sichuan University, 29 Wangjiang Road, Chengdu 610064, PR China

E-mail: *jsyou@scu.edu.cn*; *yangyudong@scu.edu.cn*

Table of Contents

I. General remarks......S1

II. Preparation of starting materials......S1

2.1 Preparation of nitroaromatic substrates......S1

2.2 Preparation of amide substrates......S3

2.3 Preparation of benzoic phenyl ester substrates......S4

III. Optimization of reaction conditions......S4

IV. General procedures for palladium- or nickel-catalyzed methylation of unconventional electrophiles......S9

V. Characterization data of starting materials......S11

VI. Characterization data of methylated products......S18

VII. Sequential methylation of 1-fluoro-4-nitronaphthalene......S34

VIII. Synthetic applications of catalytic methylation of unconventional aryl electrophiles......S35

IX. Mechanistic studies......S38

8.1 General procedure for competition experiments......S38

8.2 Competition experiment for denitrative methylation......S39

8.3 Competition experiment for decarbonylative methylation of amide......S42

8.4 Competition experiment for decarbonylative methylation of ester......S44

X. References......S45

XI. Copies of NMR spectra......S47

I. General remarks

NMR spectra were obtained on an Agilent 400-MR DD2 spectrometer. The ^{1}H NMR (400 MHz) chemical shifts were measured relative to $CDCl_3$ as the internal reference ($CDCl_3$: δ = 7.26). The ^{13}C NMR (100 MHz) chemical shifts were given using $CDCl_3$ as the internal standard ($CDCl_3$: δ = 77.16). High-resolution mass spectra (HRMS) were obtained with a Shimadzu LCMS-IT-TOF (ESI) or a Waters-Q-TOF-Premier (ESI). GC-MS spectra were recorded by Shimadzu GCMS-QP2010 SE. Infrared (IR) spectra were recorded on a Shimadzu IRTracer-100 FT-IR spectrophotometer.

Unless otherwise noted, all reagents were obtained from commercial suppliers and used without further purification. The solvents were purified and dried using Innovative Technology PS-MD-5 Solvent Purification System. $Pd(acac)_2$ were synthesized according to the literature procedures.[1] $PdCl_2$ and $Pd(OAc)_2$ were purchased from Shanxi Kaida Chemical Engineering (China) CO., Ltd.. $[Pd(allyl)Cl]_2$ was purchased from Alfa Aesar. BrettPhos were purchased from Adamas-beta Ltd.. Dcype were purchased from Sigma-Aldrich. Trimethylboroxine (TMB, 3.5*N* in THF), 4-dimethylaminopyridine (4-DMAP), DavePhos, XPhos, aryl carboxylic acids and acyl chlorides were purchased from Energy Chemical. The yields of compound **2f**, **2g** and **2h** were determined by GC analysis using calibration curves based on the data from authentic samples of the corresponding compounds. For all GC calibration curves, the ratio of molar concentration is taking as the horizontal axis and the ratio of GC area is taking as the vertical axis. Unless otherwise noted, all reactions were performed with dry solvents under an atmosphere of nitrogen in dried glassware with standard vacuum-line techniques.

II. Preparation of starting materials

2.1 Preparation of nitroaromatic substrates

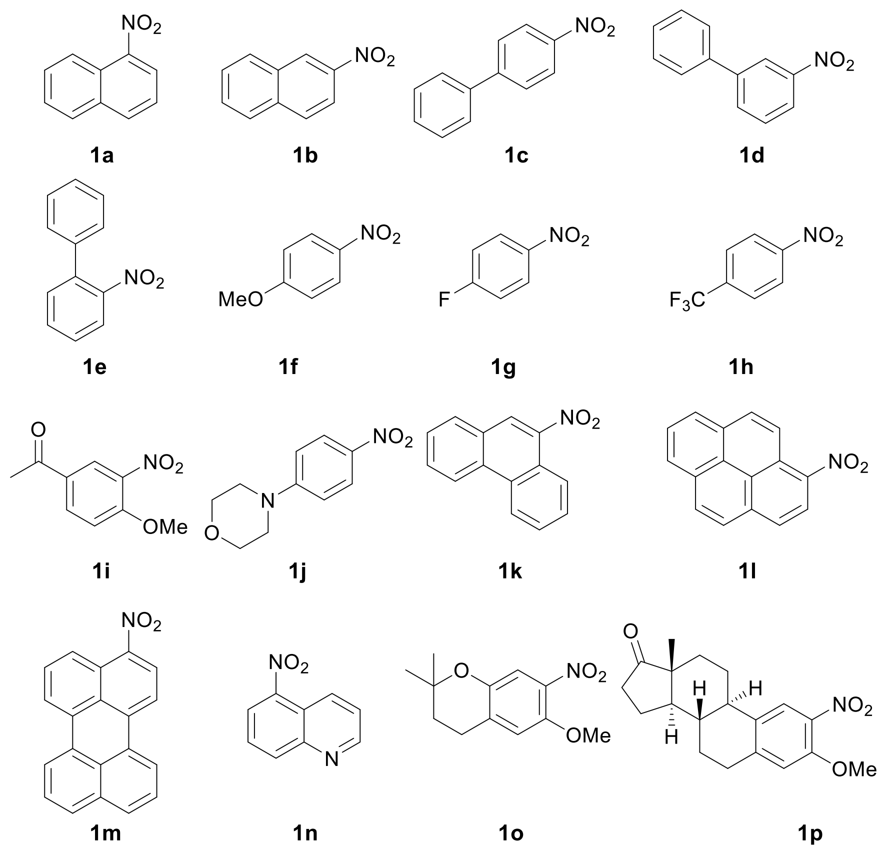

Scheme S1. Nitroaromatic substrates.

Compound **1a**, **1b**, **1f**, **1g**, **1h** and **1n** were purchased and used without further purification. Compound **1c**,[2] **1d**,[2] **1e**,[2] **1i**,[3] **1j**,[4] **1k**,[5] **1l**,[6] **1m**,[7] and **1p**[8] were prepared according to literature. The ^{1}H NMR and ^{13}C NMR data were in accordance with the related literature. Compound **1o** was prepared according to the follow procedure:

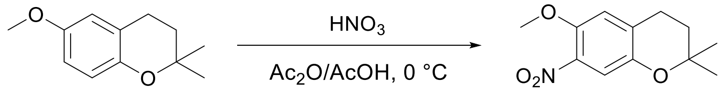

6-Methoxy-2,2-dimethyl-7-nitrochromane (**1o**) was prepared by the nitration of chromane derivative. A mixture of AcOH (1.5 mL), Ac_2O (1.5 mL) and chromane derivative (770 mg, 4 mmol) was cooled in ice bath. HNO_3 (0.35 mL) was added dropwise and stirred at 0 °C for 1 h. Then aqueous NaOH (1 mol/L) was added to neutralize the solution. The mixture was sequentially extracted with ethyl acetate (20 mL) and washed with brine (20 mL). The organic layer was dried over Na_2SO_4, filtered and concentrated under reduced pressure. The resulting crude mixture was purified by column chromatography on silica gel (200-300 mesh, petroleum ether/ethyl acetate = 6/1) to afford the desired product as yellow oil (712 mg, 75% yield). ^{1}H NMR (400 MHz, $CDCl_3$): δ = 1.36 (s, 6H), 1.86 (t, *J* = 6.8 Hz, 2H), 2.82 (t, *J* = 6.8 Hz, 2H), 3.78 (s, 3H), 6.87 (dt, *J* = 3.2, 1.2 Hz, 1H), 7.20 (d, *J* = 3.2 Hz, 1H) ppm. ^{13}C NMR (100 MHz, $CDCl_3$) δ =

22.9, 26.8, 32.2, 56.1, 76.2, 107.8, 120.9, 125.4, 142.7, 151.3 ppm. HRMS (ESI$^+$) calcd for $C_{12}H_{16}NO_4$ $[M+H]^+$ 238.1074, found 238.1075. IR (KBr): 2974, 2936, 2837, 1530, 1479, 1370, 1261, 1205, 1117, 1052, 924, 768 cm^{-1}.

2.2 Preparation of amide substrates

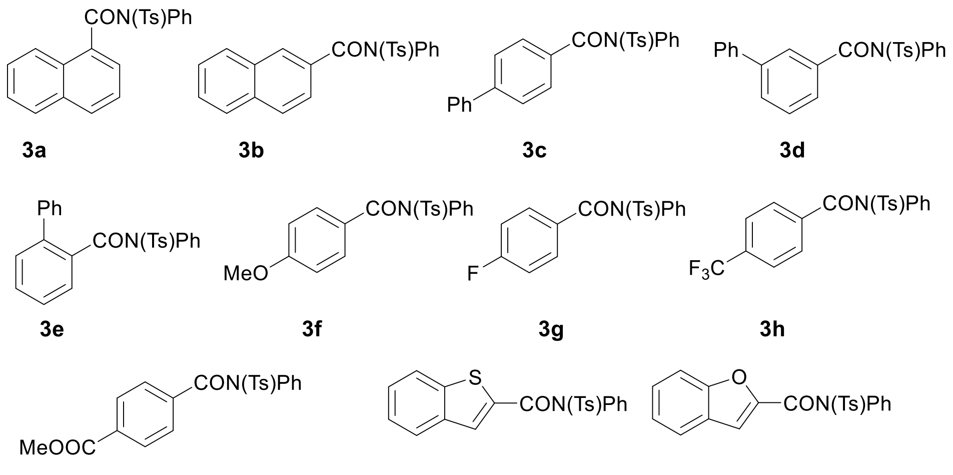

Scheme S2. Amide substrates.

General procedure: The amide substrates were prepared by a modified procedure according to the report.[9] The corresponding benzoyl chloride (5 mmol, 1.0 equiv) was added to a mixture of aniline (0.51 g, 5.5 mmol, 1.1 equiv), triethylamine (1.4 mL, 10 mmol, 2 equiv), 4-DMAP (31 mg, 0.25 mmol, 0.05 equiv) and DCM (10 mL) at 0 °C. The reaction mixture was stirred at room temperature overnight, and then diluted with DCM (25 mL). The mixture was washed with 1 *N* HCl (15 mL), saturated aqueous $NaHCO_3$ (15 mL), and brine (15 mL). Then the organic phase was dried over Na_2SO_4, filtered and concentrated under reduced pressure. The crude was recrystallized in ethanol. The resulting *NH*-free amide product (5 mmol) was dissolved in THF (25 mL). LiHMDS (1 mol/L in THF, 7.5 mL, 1.5 equiv) was added slowly at 0 °C. After stirring at 0 °C for 1 h, TsCl (1.14 g, 1.2 equiv) was added slowly. Then the reaction mixture was quenched by water after further stirring at room temperature for 15 h. The mixture was sequentially washed with 1 *N* HCl (15 mL), saturated aqueous $NaHCO_3$ (15 mL), and brine (15 mL). The organic layer was dried over Na_2SO_4, filtered and concentrated under reduced pressure. The resulting crude mixture was purified by column chromatography on silica gel (200-300 mesh) to afford the desired product.

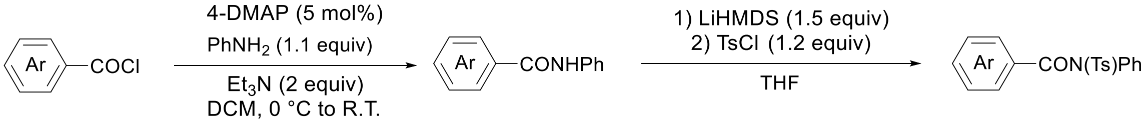

2.3 Preparation of benzoic phenyl ester substrates

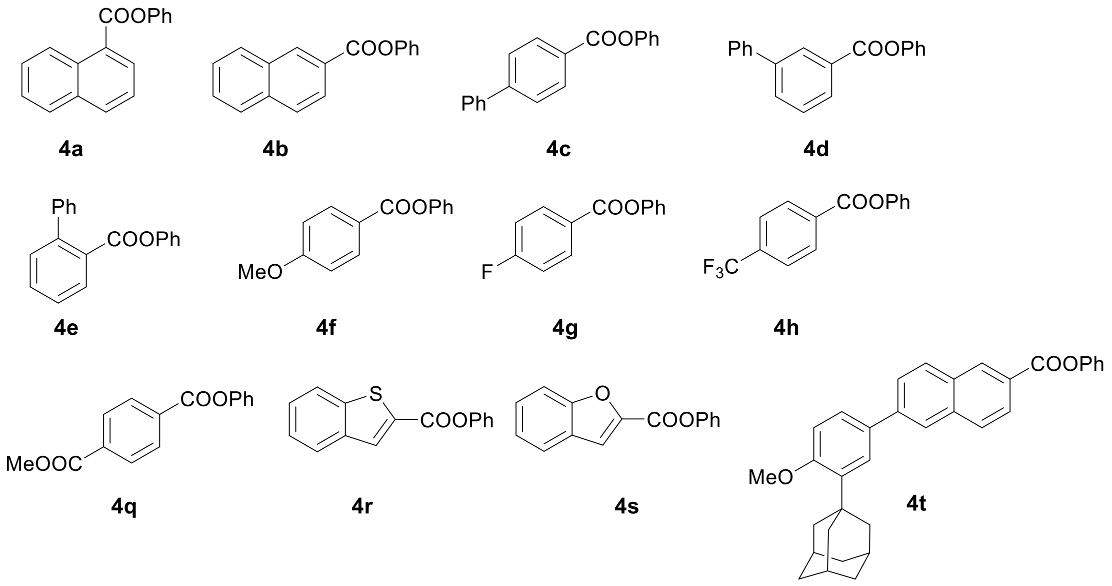

Scheme S3. Benzoic phenyl ester substrates.

General procedure: The corresponding benzoyl chloride (5 mmol, 1.0 equiv) was added to a mixture of phenol (0.51 g, 5.5 mmol, 1.1 equiv), triethylamine (1.4 mL, 10 mmol, 2 equiv), 4-DMAP (31 mg, 0.25 mmol, 0.05 equiv) and DCM (10 mL) at 0 °C. The reaction mixture was stirred at room temperature overnight, and then diluted with DCM (25 mL). The mixture was washed with 1 *N* HCl (15 mL), saturated aqueous $NaHCO_3$ (15 mL), and brine (15 mL). The organic phase was dried over Na_2SO_4, filtered and concentrated under reduced pressure. The product was further purified by recrystallization from ethanol.

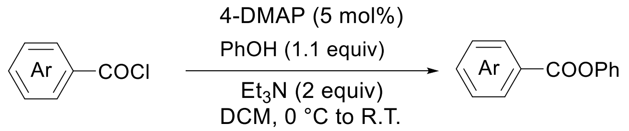

III. Optimization of reaction conditions

General procedure for reaction optimizations:

An oven-dried vial equipped with a stirring bar was charged with substrate **1a**/**3a**/**4a** (0.2 mmol, 1.0 equiv), TMB (100 µL, 3.5*N* in THF, 3.5 mmol, 1.75 equiv), catalyst, ligand and base (0.4 mmol, 2 equiv) under N_2. Solvent (0.6 mL) was added at room temperature. The reaction mixture was placed in a preheated oil bath at indicated temperature, and stirred for the indicated time. Next, the reaction mixture was cooled down to room temperature, diluted with CH_2Cl_2 (10 mL), filtered through celite, and concentrated under reduced

pressure. Purification by column chromatography on silica gel (200-300 mesh, petroleum ether) afforded the product **2a**.

Table S1. Examination of protecting group on amide.[a]

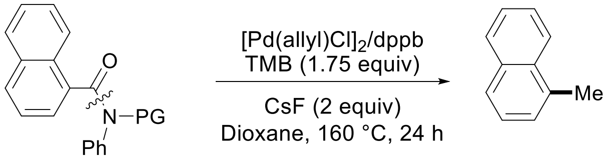

2a

Protecting group:	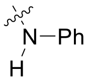	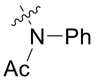	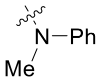	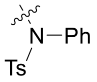	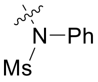	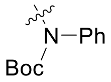
Yield of **2a**:	n.d.	<10%	trace	85%	n.d.	n.d.

[a] Reaction conditions: amide (0.2 mmol, 1 equiv), TMB (100 μL, 3.5N in THF, 3.5 mmol, 1.75 equiv), [Pd(allyl)Cl]2 (5 mol%), dppb (20 mol%), CsF (2 equiv) in 1,4-dioxane (0.6 mL), 160 °C, 24 h. Isolated yields. dppb = 1,4-diphenyl phosphinobutane.

Table S2. Optimization of the reaction conditions for palladium-catalyzed decarbonylative methylation of amides.[a]

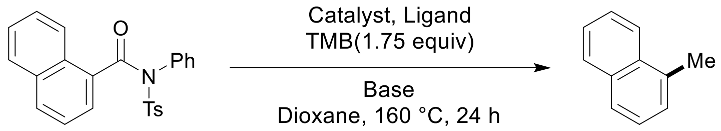

3a **2a**

Entry	Catalyst (10 mol%)	Ligand (20 mol%)	Base (2 equiv)	Yield (%)[b]
1	$Pd(OAc)_2$	Xantphos	CsF	30
2	$Pd(OAc)_2$	dppp	CsF	23
3	$Pd(OAc)_2$	dppf	CsF	35
4	$Pd(OAc)_2$	dcype	CsF	52
5	$Pd(OAc)_2$	dppb	CsF	65
6	$Pd(OAc)_2$	DPEPhos	CsF	60
7	$Pd(OAc)_2$	IPr·HCl	CsF	Trace
8	$PdCl_2$	dppb	CsF	32
9	$Pd(acac)_2$	dppb	CsF	42
10	$Pd(en)(NO_3)_2$	dppb	CsF	40
11	$Pd(COD)Cl_2$	dppb	CsF	45
12	$[Pd(allyl)Cl]_2$(5 mol%)	dppb	CsF	85
13	$[Pd(allyl)Cl]_2$ (5 mol%)	dppb	Cs_2CO_3	<10
14	$[Pd(allyl)Cl]_2$ (5 mol%)	dppb	K_3PO_4	35
15[c]	$[Pd(allyl)Cl]_2$ (5 mol%)	dppb	CsF	38
16[d]	$[Pd(allyl)Cl]_2$ (5 mol%)	dppb	CsF	55

[a] Reaction conditions: amide **3a** (0.2 mmol, 1 equiv), TMB (100 μL, 3.5*N* in THF, 3.5 mmol, 1.75 equiv), catalyst (10 mol%), ligand (20 mol%), base (2 equiv), solvent (0.6 mL) at 160 °C, 24 h. [b] Isolated yield. [c] Toluene was used as solvent. [d] $MeB(OH)_2$ (5 equiv) was used instead of TMB. Xantphos = 4,5-bis(diphenylphosphino)-9,9-dimethylxanthene, dppb = 1,4-diphenyl phosphinobutane, dppp = 1,3-bis(diphenylphosphino)propane, dppf = 1,1'-bis(diphenylphosphino)ferrocene, dcype = 1,2-bis(dicyclohexylphosphino)ethane, DPEPhos = bis[(2-diphenylphosphino)phenyl] ether, IPr·HCl = 1,3-bis(2,6-diisopropylphenyl)imidazolium chloride, en = ethylenediamine, COD = 1,5-cyclooctadiene.

Table S3. Optimization of the reaction conditions for palladium-catalyzed decarbonylative methylation of benzoic phenyl esters.[a]

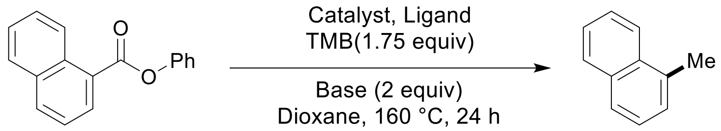

4a **2a**

Entry	Catalyst (10 mol%)	Ligand (20 mol%)	Base	Yield(%)[b]
1	$Pd(OAc)_2$	DPEPhos	CsF	n.d.
2	$[Pd(allyl)Cl]_2$ (5 mol%)	dppb	CsF	n.d.
3	$Pd(OAc)_2$	dcype	CsF	72
4	$Pd(OAc)_2$ (5 mol%)	dcype	CsF	70
5	$Pd(OAc)_2$	dcype	Cs_2CO_3	trace
6[c]	$Pd(OAc)_2$	dcype	CsF	45
7	$Ni(COD)_2$	dcype	CsF	40
8	$Ni(COD)_2$	*n*Bu_3P (40 mol%)	CsF	48
9	$Pd(OAc)_2$	dppp	CsF	n.d.
10	$Pd(en)(NO_3)_2$	dppb	CsF	40
11	$PdCl_2$	dppb	CsF	45
12	$Pd_2(dba)_3$ (5 mol%)	dppb	CsF	28
13[c]	$Pd(OAc)_2$ (5 mol%)	dcype	CsF	36
14[d]	$Pd(OAc)_2$	dcype	CsF	trace

[a] Reaction conditions: ester **4a** (0.2 mmol, 1 equiv), TMB (100 μL, 3.5*N* in THF, 3.5 mmol, 1.75 equiv), catalyst (10 mol%), ligand (20 mol%), base (2 equiv), solvent (0.6 mL) at 160 °C, 24 h. [b] Isolated yield. [c] Toluene was used as solvent. [d] $MeB(OH)_2$ (5 equiv) was used instead of TMB. dppb = 1,4-diphenyl phosphinobutane, dppp = 1,3-bis(diphenylphosphino)propane, dcype = 1,2-bis(dicyclohexylphosphino)ethane, DPEPhos = bis[(2-diphenylphosphino)phenyl] ether, en = ethylenediamine, COD = 1,5-cyclooctadiene.

Table S4. Non-decarbonylative methylation of amide.[a]

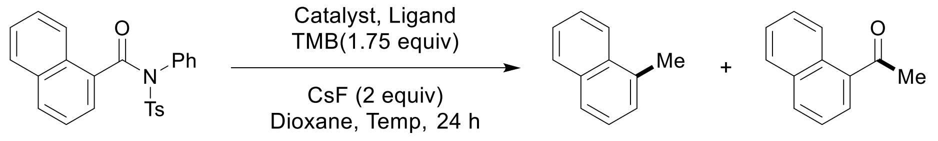

Entry	Catalyst (10 mol%)	Ligand (20 mol%)	Temp (°C)	Yield of **2a** (%)[b]	Yield of **2a'**(%)[b]
1	$[Pd(allyl)Cl]_2$	dppb	110	40	24
2	$[Pd(allyl)Cl]_2$	dppb	60	n.d.	n.d.
3	$[Pd(allyl)Cl]_2$	PCy_3	60	n.d.	30
4	$Ni(COD)_2$	PCy_3	60	n.d.	n.d.
5	$Ni(COD)_2$	SIPr·HCl/KO*t*Bu	60	18	n.d.

[a] Reaction conditions: amide **3a** (0.2 mmol, 1 equiv), TMB (100 µL, 3.5*N* in THF, 3.5 mmol, 1.75 equiv), catalyst (10 mol%), ligand (20 mol%), CsF (2 equiv), solvent (0.6 mL), 24 h. [b] Isolated yield. dppb = 1,4-diphenyl phosphinobutane, Cy = cyclohexyl, COD = 1,5-cyclooctadiene, SIPr = 1,3-bis(2,6-diisopropylphenyl)-4,5-dihydroimidazol-2-ylidine.

Table S5. Non-decarbonylative methylation of ester.[a]

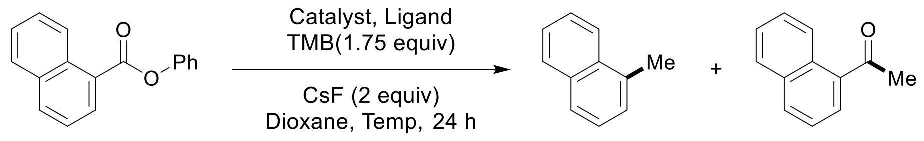

Entry	Catalyst (10 mol%)	Ligand (20 mol%)	Temp (°C)	Yield of **2a** (%)[b]	Yield of **2a'**(%)[b]
1	$Pd(OAc)_2$	dcype	110	36	<10
2	$Pd(OAc)_2$	dcype	60	n.d.	n.d.
3	$Pd(OAc)_2$	PCy_3	60	n.d.	35
4	$Ni(COD)_2$	PCy_3	60	n.d.	n.d.
5	$Ni(COD)_2$	SIPr·HCl/KO*t*Bu	60	n.d.	n.d.

[a] Reaction conditions: ester **4a** (0.2 mmol, 1 equiv), TMB (100 µL, 3.5*N* in THF, 3.5 mmol, 1.75 equiv), catalyst (10 mol%), ligand (20 mol%), base (2 equiv), solvent (0.6 mL) at 160 °C, 24 h. [b] Isolated yield. dcype = 1,2-bis(dicyclohexylphosphino)ethane, Cy = cyclohexyl, COD = 1,5-cyclooctadiene, SIPr = 1,3-bis(2,6-diisopropylphenyl)-4,5-dihydroimidazol-2-ylidine.

IV. General procedures for palladium- or nickel-catalyzed methylation of unconventional electrophiles

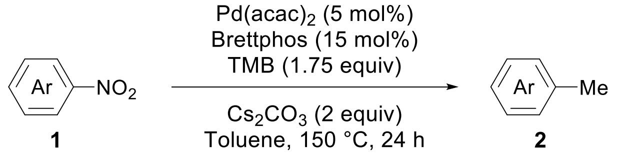

Denitrative methylation of nitrobenzene 1: An oven-dried vial equipped with a stirring bar was charged with nitroarene **1** (0.2 mmol, 1.0 equiv), TMB (100 μL, 3.5*N* in THF, 3.5 mmol, 1.75 equiv), $Pd(acac)_2$ (3.1 mg, 5 mol%), BrettPhos (16.1 mg, 15 mol%), Cs_2CO_3 (130 mg, 2 equiv) and toluene (0.6 mL) under N_2 at room temperature. The reaction mixture was placed in a preheated oil bath at 150 °C, and stirred for the indicated time. Then the reaction mixture was cooled down to room temperature, diluted with CH_2Cl_2 (10 mL), filtered through celite, and concentrated under reduced pressure. Purification by column chromatography on silica gel (200-300 mesh) afforded the corresponding methylating product **2**.

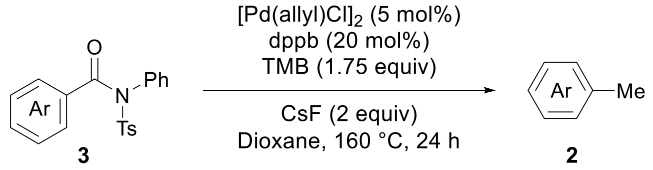

Decarbonylative methylation of benzamide 3: An oven-dried vial equipped with a stirring bar was charged with benzamide **3** (0.2 mmol, 1.0 equiv), TMB (100 μL, 3.5*N* in THF, 3.5 mmol, 1.75 equiv), $[Pd(allyl)Cl]_2$ (3.9 mg, 5 mol%), dppb (17.1 mg, 20 mol%), CsF (61 mg, 2.0 equiv) and dioxane (0.6 mL) under N_2 at room temperature. The reaction mixture was placed in a preheated oil bath at 160 °C, and stirred for the indicated time. After the indicated time, the reaction mixture was cooled down to room temperature, diluted with CH_2Cl_2 (10 mL), filtered through celite, and concentrated under reduced pressure. Purification by column chromatography on silica gel (200-300 mesh) afforded the corresponding methylating product **2**.

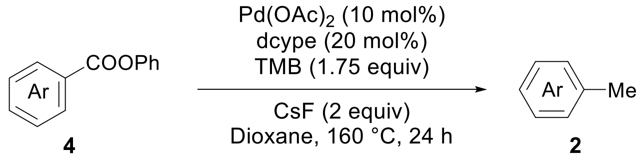

Decarbonylative methylation of benzoic phenyl ester 4: An oven-dried vial equipped with a stirring bar was charged with benzoic phenyl ester **4** (0.2 mmol, 1.0 equiv), TMB (100 μL, 3.5*N* in THF, 3.5 mmol, 1.75 equiv), $Pd(OAc)_2$ (2.2 mg, 5 mol%), dcype (8.5 mg, 10 mol%), CsF (61mg, 2 equiv) and dioxane (0.6 mL) under N_2 at room temperature. The reaction mixture was placed in a preheated oil bath at 160 °C, and stirred for the indicated time. Then the reaction mixture was

cooled down to room temperature, diluted with CH_2Cl_2 (10 mL), filtered through celite, and concentrated under reduced pressure. Purification by column chromatography on silica gel (200-300 mesh) afforded the corresponding methylating product **2**.

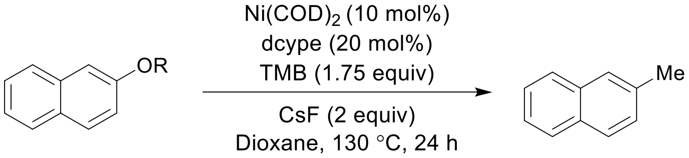

5a, R = Me
5b, R = Piv

2b

Methylation of 1-naphthyl methyl ether 5a and 1-naphthyl pivalate 5b: An oven-dried vial equipped with a stirring bar was charged with 1-naphthyl methyl ether **5a** or 1-naphthyl pivalate **5b** (0.2 mmol, 1.0 equiv), TMB (100 µL, 3.5*N* in THF, 3.5 mmol, 1.75 equiv), $Ni(COD)_2$ (5.5 mg, 10 mol%), dcype (17 mg, 20 mol%), CsF (61mg, 2.0 equiv) and dioxane (0.6 mL) under N_2 at room temperature. The reaction mixture was placed in a preheated oil bath at 130 °C, and stirred for 24 h. The reaction mixture was then cooled down to room temperature, diluted with CH_2Cl_2 (10 mL), filtered through celite, and concentrated. Purification by column chromatography on silica gel (200-300 mesh, petroleum ether) afforded 2-methylnaphthalene (**2b**) as colorless liquid (R = Me, 14 mg, 48% yield; R = Piv, 23 mg, 82% yield).

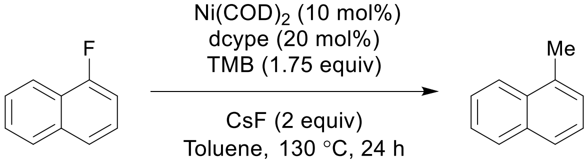

6 **2a**

Defluoromethylation of 1-fluoro naphthalene 6: An oven-dried vial equipped with a stirring bar was charged with 1-fluoro naphthalene **6** (0.2 mmol, 1.0 equiv), TMB (100 µL, 3.5*N* in THF, 3.5 mmol, 1.75 equiv), $Ni(COD)_2$ (5.5 mg, 10 mol%), dcype (17 mg, 20 mol%), CsF (61mg, 2.0 equiv) and toluene (0.6 mL) under N_2 at room temperature. The reaction mixture was placed in a preheated oil bath at 130 °C, and stirred for 24 h. The reaction mixture was then cooled down to room temperature, diluted with CH_2Cl_2 (10 mL), filtered through celite, and concentrated. Purification by column chromatography on silica gel (200-300 mesh, petroleum ether) afforded 1-methylnaphthalene (**2a**) as colorless liquid (20 mg, 70% yield).

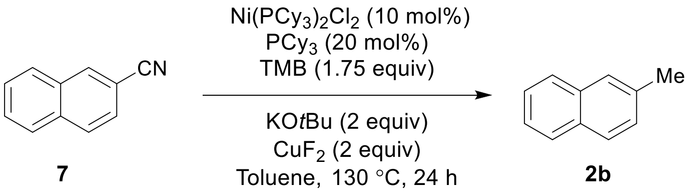

7 **2b**

Decyanative methylation of 2-naphthonitrile 7: An oven-dried vial equipped with a stirring bar was charged with 2-naphthonitrile **7** (0.2 mmol, 1.0 equiv), TMB (100 μL, 3.5*N* in THF, 3.5 mmol, 1.75 equiv), $Ni(PCy_3)Cl_2$ (13.8 mg, 10 mol%), PCy_3 (11.2 mg, 20 mol%), KO*t*Bu (45mg, 2.0 equiv), CuF_2 (40.6 mg, 2.0 equiv) and toluene (0.6 mL) under N_2 at room temperature. The reaction mixture was placed in a preheated oil bath at 130 °C, and stirred for 24 h. The reaction mixture was then cooled down to room temperature, diluted with CH_2Cl_2 (10 mL), filtered through celite, and concentrated. Purification by column chromatography on silica gel (200-300 mesh) afforded 2-methylnaphthalene (**2b**) as colorless liquid (18 mg, 65% yield).

V. Characterization data of starting materials

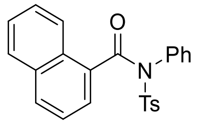

***N*-Phenyl-*N*-tosyl-1-naphthamide (3a)**

According to the general procedure for amide synthesis, **3a** was obtained as a white solid (petroleum ether/EtOAc = 8/1 to 4/1). ^1H NMR (400 MHz, $CDCl_3$): δ 2.50 (s, 3H), 7.07 – 7.14 (m, 5H), 7.19 (t, *J* = 7.7 Hz, 1H), 7.34 (dd, *J* = 7.2, 1.2 Hz, 1H), 7.38 (d, *J* = 8.6 Hz, 2H), 7.41 – 7.50 (m, 2H), 7.67 – 7.72 (m, 2H), 7.89 (d, *J* = 8.4 Hz, 1H), 7.98 (d, *J* = 8.4 Hz, 2H) ppm. ^{13}C NMR (100 MHz, $CDCl_3$) δ 21.9, 124.2, 124.6, 126.5, 126.7, 127.4, 128.4, 129.0, 129.2, 129.6, 130.0, 130.1, 130.8, 132.2, 133.2, 135.7, 136.8, 145.2, 169.9 ppm. HRMS (ESI$^+$) calcd for $C_{24}H_{20}NO_3S$ $[M+H]^+$ 402.1158, found 402.1162. IR (KBr): 3063, 3026, 2921, 1688, 1486, 1363, 1279, 1172, 1075, 944, 757, 696 cm^{-1}.

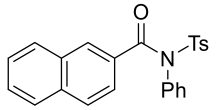

***N*-Phenyl-*N*-tosyl-2-naphthamide (3b)**

According to the general procedure for amide synthesis, **3b** was obtained as a white solid (petroleum ether/EtOAc = 8/1 to 4/1). ^1H NMR (400 MHz, $CDCl_3$): δ = 2.46 (s, 3H), 7.20 – 7.26 (m, 5H), 7.33 (d, *J* = 8.0 Hz, 2H), 7.41 – 7.52 (m, 3H), 7.60 (d, *J* = 8.8 Hz, 1H), 7.71 (d, *J* = 8.8 Hz, 2H), 7.85 (d, *J* = 8.4 Hz, 2H), 8.04 (s, 1H) ppm. ^{13}C NMR (100 MHz, $CDCl_3$) δ 21.9, 125.2, 126.8, 127.8, 127.9, 128.4, 129.1, 129.2, 129.3, 129.4, 129.7, 130.5, 131.0, 131.4, 132.2, 134.6, 135.3, 137.6, 145.0, 170.1 ppm. HRMS (ESI$^+$) calcd for $C_{24}H_{20}NO_3S$ $[M+H]^+$ 402.1158, found 402.1159. IR (KBr): 3113, 2930, 2852, 1697, 1608, 1585, 1531, 1520, 1347, 1252, 1106, 814, 771, 742 cm^{-1}.

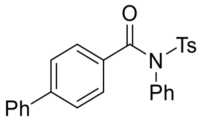

***N*-Phenyl-*N*-tosyl-(1,1'-biphenyl)-4-carboxamide (3c)**

According to the general procedure for amide synthesis, **3c** was obtained as a white solid (petroleum ether/EtOAc = 8/1 to 4/1). ^{1}H NMR (400 MHz, $CDCl_3$): δ = 2.46 (s, 3H), 7.19 – 7.23 (m, 2H), 7.29 – 7.36 (m, 6H), 7.37 – 7.41 (m, 4H), 7.46 – 7.49 (m, 2H), 7.52 – 7.54 (m, 2H), 7.84 (d, *J* = 8.4 Hz, 2H) ppm. ^{13}C NMR (100 MHz, $CDCl_3$): δ = 21.9, 126.7, 127.2, 128.3, 129.0, 129.2, 129.3, 129.4, 129.6, 130.3, 130.5, 132.3, 135.3, 137.6, 139.6, 144.5, 144.9, 169.8 ppm. HRMS (ESI$^+$) calcd for $C_{26}H_{22}NO_3S$ $[M+H]^+$ 428.1315, found 428.1324. IR (KBr): 3074, 2920, 2853, 1690, 1593, 1485, 1366, 1256, 1171, 1090, 745, 699 cm^{-1}.

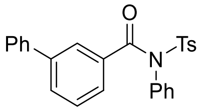

***N*-Phenyl-*N*-tosyl-(1,1'-biphenyl)-3-carboxamide (3d)**

According to the general procedure for amide synthesis, **3d** was obtained as a white solid (petroleum ether/EtOAc = 8/1 to 4/1). ^{1}H NMR (400 MHz, $CDCl_3$): δ = 2.45 (s, 3H), 7.18 – 7.23 (m, 2H), 7.23 – 7.26 (m, 1H), 7.29 – 7.43 (m, 11H), 7.50 (ddd, *J* = 7.6, 2.0, 1.2 Hz, 1H), 7.66 (t, *J* = 1.6 Hz, 1H), 7.85 (d, *J* = 8.4 Hz, 2H) ppm. ^{13}C NMR (100 MHz, $CDCl_3$): δ = 21.9, 127.1, 127.9, 128.39, 128.42, 128.6, 128.9, 129.28, 129.33, 129.4, 129.6, 130.5, 130.6, 134.2, 135.3, 137.6, 139.8, 141.1, 145.0, 169.9 ppm. HRMS (ESI$^+$) calcd for $C_{26}H_{22}NO_3S$ $[M+H]^+$ 428.1315, found 428.1314. IR (KBr): 3072, 2921, 2865, 1699, 1594, 1494, 1376, 1162, 1086, 947, 886, 701 cm^{-1}.

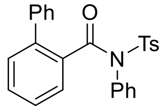

***N*-Phenyl-*N*-tosyl-(1,1'-biphenyl)-2-carboxamide (3e)**

According to the general procedure for amide synthesis, **3e** was obtained as a white solid (petroleum ether/EtOAc = 8/1 to 4/1). ^{1}H NMR (400 MHz, $CDCl_3$): δ = 2.49 (s, 3H), 6.39 (d, *J* = 7.6 Hz, 2H), 7.03 (t, *J* = 7.6 Hz, 2H), 7.10 – 7.16 (m, 2H), 7.16 – 7.23 (m, 3H), 7.23 – 7.29 (m, 1H), 7.29 – 7.37 (m, 5H), 7.38 – 7.43 (m, 1H), 7.83 (d, *J* = 8.4 Hz, 2H) ppm. ^{13}C NMR (100 MHz, $CDCl_3$): δ = 21.9, 126.9, 128.1, 128.3, 128.6, 128.9, 129.0, 129.4, 129.5, 129.7, 130.4, 130.6, 134.6, 135.2,

136.0, 138.5, 139.0, 144.9, 171.0 ppm. HRMS (ESI$^+$) calcd for $C_{26}H_{22}NO_3S$ $[M+H]^+$ 428.1315, found 428.1325. IR (KBr): 3066, 2923, 2850, 1700, 1353, 1280, 1173, 1085, 958, 745, 692 cm^{-1}.

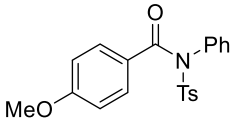

***N*-Phenyl-*N*-tosyl-4-methoxyl-1-benzamide (3f)**

According to the general procedure for amide synthesis, **3f** was obtained as a white solid (petroleum ether/EtOAc = 8/1 to 4/1). ^1H NMR (400 MHz, $CDCl_3$): δ = 2.44 (s, 3H), 3.72 (s, 3H), 6.65 (d, J = 8.8 Hz, 2H), 7.16 – 7.19 (m, 2H), 7.28 – 7.30 (m, 5H), 7.47 (d, J = 8.8 Hz, 2H), 7.78 (d, J = 8.4 Hz, 2H) ppm. ^{13}C NMR (100 MHz, $CDCl_3$): δ = 21.8, 55.4, 113.4, 125.5, 129.0, 129.2, 129.3, 129.6, 130.3, 132.2, 135.4, 138.0, 144.7, 162.5, 169.5 ppm. HRMS (ESI$^+$) calcd for $C_{21}H_{20}NO_4S$ $[M+H]^+$ 382.1108, found 382.1109. The NMR spectra are in accordance with literature.[10]

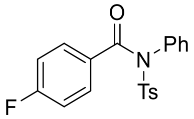

4-Fluoro-*N*-phenyl-*N*-tosylbenzamide (3g)

According to the general procedure for amide synthesis, **3g** was obtained as a white solid (petroleum ether/Ether = 4/1). ^1H NMR (400 MHz, $CDCl_3$): δ = 2.45 (s, 3H), 6.82 – 6.89 (m, 2H), 7.12 – 7.17 (m, 2H), 7.27 – 7.35 (m, 5H), 7.45 – 7.50 (m, 2H), 7.78 – 7.83 (m, 2H) ppm. ^{13}C NMR (100 MHz, $CDCl_3$): δ = 21.9, 115.43 (d, J = 22.1 Hz), 129.3, 129.38, 129.39, 129.6, 129.77 (d, J = 3.1 Hz), 130.4, 132.26 (d, J = 9.3 Hz), 135.1, 137.5, 145.1, 164.58 (d, J = 254.1 Hz), 168.9 ppm. ^{19}F NMR (376 MHz, $CDCl_3$): δ = -105.96 ppm. HRMS (ESI$^+$) calcd for $C_{20}H_{17}FNO_3S$ $[M+H]^+$ 370.0908, found 370.0913. The NMR spectra are in accordance with literature.[10]

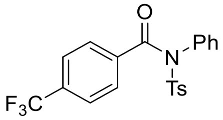

***N*-Phenyl-*N*-tosyl-4-(trifluoromethyl)benzamide (3h)**

According to the general procedure for amide synthesis, **3h** was obtained as a white solid (petroleum ether/ether = 8/1 to 4/1). ^1H NMR (400 MHz, $CDCl_3$): δ = 2.46 (s, 3H), 7.11 – 7.16 (m, 2H), 7.27 – 7.36 (m, 5H), 7.43 (d, J = 8.3 Hz, 2H), 7.52 (d, J = 8.2 Hz, 2H), 7.84 (d, J = 8.4 Hz, 2H)

ppm. ^{13}C NMR (100 MHz, $CDCl_3$): δ = 21.9, 123.4 (q, J = 271.0 Hz), 125.2 (q, J = 3.7 Hz), 129.51, 129.53, 129.6, 129.7, 130.5, 133.1 (q, J = 32.8 Hz), 134.9, 136.9, 137.2 (q, J = 1.0 Hz), 145.4, 168.7 ppm. ^{19}F NMR (376 MHz, $CDCl_3$): δ = -63.2 ppm. HRMS (ESI^+) calcd for $C_{21}H_{16}F_3NNaO_3S$ $[M+Na]^+$ 442.0695, found 442.0695. The NMR spectra are in accordance with literature.[10]

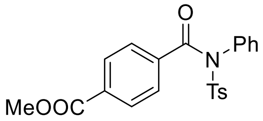

Methyl 4-(phenyl(tosyl)carbamoyl)benzoate (3q)

According to the general procedure for amide synthesis, **3q** was obtained as a white solid (petroleum ether/EtOAc = 3/1 to 1/1). ^1H NMR (400 MHz, $CDCl_3$): δ = 2.46 (s, 3H), 3.85 (s, 3H), 7.12 – 7.15 (m, 2H), 7.24 – 7.30 (m, 3H), 7.33 (d, J = 8.0 Hz, 2H), 7.45 – 7.48 (m, 2H), 7.80 – 7.87 (m, 4H) ppm. ^{13}C NMR (100 MHz, $CDCl_3$): δ = 21.9, 52.5, 129.3, 129.4, 129.48, 129.50, 129.6, 130.5, 132.6, 135.0, 137.0, 137.9, 145.3, 166.1, 169.2 ppm. HRMS (ESI^+) calcd for $C_{22}H_{20}NO_5S$ $[M+H]^+$ 410.1057, found 410.1057. The NMR spectra are in accordance with literature.[10]

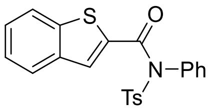

***N*-Phenyl-*N*-tosylbenzo[*b*]thiophene-2-carboxamide (3r)**

According to the general procedure for amide synthesis, **3r** was obtained as a white solid (petroleum ether/EtOAc = 8/1 to 4/1). ^1H NMR (400 MHz, $CDCl_3$): δ = 2.46 (s, 3H), 7.19 (s, 1H), 7.29 (d, J = 8.0 Hz, 1H), 7.32 – 7.38 (m, 4H), 7.39 (s, 1H), 7.47 (t, J = 7.6 Hz, 2H), 7.52 (m, 1H), 7.60 (d, J = 8.0 Hz, 1H), 7.67 (d, J = 8.0 Hz, 1H), 7.91 (d, J = 8.4 Hz, 2H) ppm. ^{13}C NMR (100 MHz, $CDCl_3$): δ = 21.9, 122.4, 124.9, 125.8, 127.3, 129.5, 129.7, 129.8, 130.4, 131.1, 132.2, 135.5, 136.2, 136.7, 138.1, 142.4, 145.2, 162.9 ppm. HRMS (ESI^+) calcd for $C_{22}H_{18}NO_3S_2$ $[M+H]^+$ 408.0723, found 408.0727. IR (KBr): 3093, 3067, 3033, 2929, 1661, 1508, 1367, 1189, 1178, 753, 701, 679 cm^{-1}.

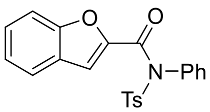

***N*-Phenyl-*N*-tosylbenzofuran-2-carboxamide (3s)**

According to the general procedure for amide synthesis, **3s** was obtained as a white solid (petroleum ether/EtOAc = 8/1 to 4/1). ^1H NMR (400 MHz, $CDCl_3$): δ = 2.45 (s, 3H), 6.38 (s, 1H),

7.15 – 7.20 (m, 1H), 7.31 – 7.37 (m, 6H), 7.41 – 7.55 (m, 4H), 7.95 (d, *J* = 8.4 Hz, 2H) ppm. ^{13}C NMR (100 MHz, $CDCl_3$): δ = 21.9, 112.1, 115.6, 123.0, 123.9, 126.6, 128.1, 129.5, 129.7, 129.8, 130.3, 130.6, 135.4, 136.6, 145.3, 146.2, 155.1, 159.1 ppm. HRMS (ESI$^+$) calcd for $C_{22}H_{18}NO_4S$ $[M+H]^+$ 392.0951, found 392.0956. IR (KBr): 3074, 3015, 2915, 1676, 1552, 1488, 1363, 1181, 1086, 1001, 938, 750, 701, 691 cm^{-1}.

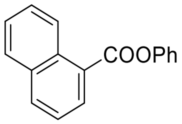

Phenyl 1-naphthoate (4a)

According to the general procedure for ester synthesis, **4a** was obtained as a white solid. ^{1}H NMR (400 MHz, $CDCl_3$): δ = 7.28 – 7.35 (m, 3H), 7.49 (t, *J* = 7.9 Hz, 2H), 7.55 – 7.62 (m, 2H), 7.63 – 7.68 (m, 1H), 7.94 (d, *J* = 8.0 Hz, 1H), 8.12 (d, *J* = 8.2 Hz, 1H), 8.49 (d, *J* = 7.3 Hz, 1H), 9.05 (d, *J* = 8.7 Hz, 1H) ppm. ^{13}C NMR (100 MHz, $CDCl_3$) δ = 122.0, 124.7, 125.9, 126.0, 126.1, 126.5, 128.3, 128.8, 129.7, 131.4, 131.8, 134.0, 134.5, 151.1, 166.0 ppm. HRMS (ESI$^+$) calcd for $C_{17}H_{12}NaO_2$ $[M+Na]^+$ 271.0730, found 271.0734. The NMR spectra are in accordance with literature.[11]

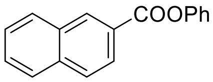

Phenyl 2-naphthoate (4b)

According to the general procedure for ester synthesis, **4b** was obtained as a white solid. ^{1}H NMR (400 MHz, $CDCl_3$): δ = 6.65 (dd, *J* = 8.0 Hz, 0.8 Hz, 1H), 7.18 – 7.20 (m, 2H), 7.24 – 7.29 (m, 1H) 7.40 – 7.44 (m, 5H), 7.59 – 7.61 (m, 2H), 7.89 (d, *J* = 16 Hz, 1H) ppm. ^{13}C NMR (100 MHz, $CDCl_3$): δ = 117.4, 121.8, 125.9, 128.4, 129.1, 129.6, 130.8, 134.3, 146.7, 150.9, 165.5 ppm. HRMS (ESI$^+$) calcd for $C_{17}H_{13}O_2$ $[M]^+$ 248.0832, found 248.0827. The NMR spectra are in accordance with literature.[11]

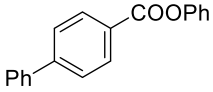

Phenyl (1,1'-biphenyl)-4-carboxylate (4c)

According to the general procedure for ester synthesis, **4c** was obtained as a white solid. ^{1}H NMR (400 MHz, $CDCl_3$): δ = 7.24 – 7.26 (m, 2H), 7.27 – 7.31 (m, 1H), 7.41 – 7.52 (m, 5H), 7.66 – 7.68 (m, 2H), 7.75 (dd, *J* = 8.4, 1.5 Hz, 2H), 8.29 (dd, *J* = 8.4, 1.6 Hz, 2H) ppm. ^{13}C NMR (100 MHz, $CDCl_3$): δ = 121.9, 126.0, 127.4, 127.5, 128.4, 128.5, 129.1, 129.6, 130.8, 140.0, 146.4, 151.1, 165.2 ppm.

HRMS (ESI$^+$) calcd for $C_{19}H_{14}O_2$ $[M+Na]^+$ 297.0886, found 297.0891. The NMR spectra are in accordance with literature.[12]

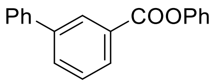

Phenyl (1,1'-biphenyl)-3-carboxylate (4d)

According to the general procedure for ester synthesis, **4d** was obtained as a white solid. ^{1}H NMR (400 MHz, $CDCl_3$): δ = 7.24 (d, *J* = 8.8 Hz, 2H), 7.29 (t, *J* = 7.6 Hz, 1H), 7.38 – 7.51 (m, 5H), 7.60 (t, *J* = 7.6 Hz, 1H), 7.66 (d, *J* = 7.2 Hz, 2H), 7.87 (d, *J* = 7.6 Hz, 1H), 8.19 (d, *J* = 7.6 Hz, 1H), 8.44 (s, 1H) ppm. ^{13}C NMR (100 MHz, $CDCl_3$): δ = 121.8, 126.1, 127.3, 128.0, 129.0, 129.07, 129.09, 129.2, 129.7, 130.2, 132.4, 140.1, 141.9, 151.1, 165.3 ppm. HRMS (ESI$^+$) calcd for $C_{19}H_{14}O_2$ $[M+Na]^+$ 297.0886, found 297.0893. The NMR spectra are in accordance with literature.[13]

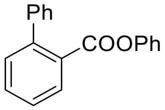

Phenyl (1,1'-biphenyl)-2-carboxylate (4e)

According to the general procedure for ester synthesis, **4e** was obtained as a white solid. ^{1}H NMR (400 MHz, $CDCl_3$): δ =6.85 (d, *J* = 7.6 Hz, 2H), 7.17 (t, *J* = 7.6 Hz, 1H), 7.31 (t, *J* = 8.0 Hz, 2H), 7.38 – 7.52 (m, 7H), 7.61 (t, *J* = 7.6 Hz, 1H), 8.00 (d, *J* = 8.0 Hz, 1H) ppm. ^{13}C NMR (100 MHz, $CDCl_3$): δ = 121.4, 125.9, 127.5, 127.6, 128.4, 128.7, 129.4, 130.4, 130.6, 131.0, 131.9, 141.4, 142.9, 150.7, 167.4 ppm. HRMS (ESI$^+$) calcd for $C_{19}H_{14}O_2$ $[M+Na]^+$ 297.0886, found 297.0890. The NMR spectra are in accordance with literature.[14]

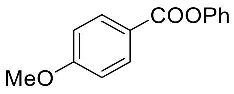

Phenyl 4-methoxylbenzoate (4f)

According to the general procedure for ester synthesis, **4f** was obtained as a white solid. ^{1}H NMR (400 MHz, $CDCl_3$): δ = 3.90 (s, 3H), 6.99 (d, *J* = 9.0 Hz, 2H), 7.19 – 7.23 (m, 2H), 7.22 – 7.32 (m, 1H), 7.40 – 7.46 (m, 2H), 8.17 (d, *J* = 9.0 Hz, 2H) ppm. ^{13}C NMR (100 MHz, $CDCl_3$): δ = 55.6, 113.9, 121.9, 122.0, 125.9, 129.6, 132.4, 151.2, 164.0, 165.1 ppm. HRMS (ESI$^+$) calcd for $C_{14}H_{13}O_3$ $[M+H]^+$ 229.0859, found 229.0860. The NMR spectra are in accordance with literature.[11]

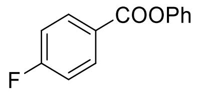

Phenyl 4-fluorobenzoate (4g)

According to the general procedure for ester synthesis, **4g** was obtained as a white solid. ^{1}H NMR (400 MHz, $CDCl_3$): δ = 7.15 – 7.24 (m, 4H), 7.26 – 7.32 (m, 1H), 7.39 – 7.48 (m, 2H), 8.18 – 8.28 (m, 2H) ppm. ^{13}C NMR (100 MHz, $CDCl_3$): δ = 115.92 (d, J = 22.0 Hz), 121.8, 125.89 (d, J = 2.9 Hz), 126.1, 129.7, 132.92 (d, J = 9.5 Hz), 150.9, 164.68 (d, J = 63.2 Hz), 167.5 ppm. ^{19}F NMR (376 MHz, $CDCl_3$): δ = -104.46 ppm. HRMS (ESI$^+$) calcd for $C_{13}H_9FNaO_2$ $[M+Na]^+$ 239.0479, found 239.0484. The NMR spectra are in accordance with literature.[11]

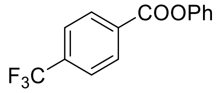

Phenyl 4-(trifluoromethyl)benzoate (4h)

According to the general procedure for ester synthesis, **4h** was obtained as a white solid. ^{1}H NMR (400 MHz, $CDCl_3$): δ = 7.20 – 7.25 (m, 2H), 7.28 – 7.34 (m, 1H), 7.41 – 7.50 (m, 2H), 7.79 (d, J = 8.4 Hz, 2H), 8.33 (d, J = 8.4 Hz, 2H) ppm. ^{13}C NMR (100 MHz, $CDCl_3$): δ = 121.7, 123.70 (q, J = 272.7 Hz), 125.75 (q, J = 3.7 Hz), 126.4, 129.8, 130.7, 132.95 (q, J = 1.2 Hz), 135.14 (q, J = 32.8 Hz), 150.8, 164.1 ppm. ^{19}F NMR (376 MHz, $CDCl_3$): δ = -63.16 ppm. HRMS (ESI$^+$) calcd for $C_{14}H_9F_3NaO_2$ $[M+Na]^+$ 289.0447, found 289.0453. The NMR spectra are in accordance with literature.[14]

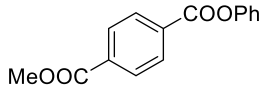

Methyl phenyl terephthalate (4q)

According to the general procedure for ester synthesis, **4q** was obtained as a white solid. ^{1}H NMR (400 MHz, $CDCl_3$): δ = 3.97 (s, 3H), 7.21 – 7.24 (m, 2H), 7.28 – 7.32 (m, 1H), 7.42 – 7.47 (m, 2H), 8.16 – 8.19 (m, 2H), 8.26 – 8.29 (m, 2H) ppm. ^{13}C NMR (100 MHz, $CDCl_3$): δ = 52.7, 121.7, 126.3, 129.7, 129.8, 130.3, 133.4, 134.6, 150.8, 164.5, 166.3 ppm. HRMS (ESI$^+$) calcd for $C_{15}H_{12}NaO_4$ $[M+Na]^+$ 279.0628, found 279.0635. The NMR spectra are in accordance with literature.[15]

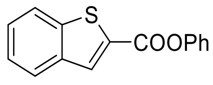

Phenyl benzo[*b*]thiophene-2-carboxylate (4r)

According to the general procedure for ester synthesis, **4r** was obtained as a white solid. ^{1}H NMR (400 MHz, $CDCl_3$): δ = 7.25 – 7.32 (m, 3H), 7.42 – 7.48 (m, 3H), 7.51 (t, J = 7.2 Hz, 1H), 7.90 – 7.95 (m, 2H), 8.26 (s, 1H) ppm. ^{13}C NMR (100 MHz, $CDCl_3$): δ = 121.7, 123.0, 125.2, 125.9, 126.3, 127.5, 129.7, 132.0, 132.8, 138.8, 142.8, 150.7, 161.4 ppm. HRMS (ESI$^+$) calcd for $C_{15}H_{10}NaO_2S$ $[M+Na]^+$ 277.0294, found 277.0291. The NMR spectra are in accordance with literature.[16]

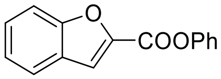

Phenyl benzofuran-2-carboxylate (4s)

According to the general procedure for ester synthesis, **4s** was obtained as a white solid. ^{1}H NMR (400 MHz, $CDCl_3$): δ = 7.26 – 7.32 (m, 3H), 7.34 – 7.38 (m, 1H), 7.43 – 7.47 (m, 2H), 7.48 – 7.53 (m, 1H), 7.65 (d, J = 8.4 Hz, 1H), 7.73 – 7.76 (m, 2H) ppm. ^{13}C NMR (100 MHz, $CDCl_3$): δ = 112.6, 115.6, 121.7, 123.2, 124.1, 126.4, 127.0, 128.2, 129.7, 144.9, 150.3, 156.2, 158.0 ppm. HRMS (ESI$^+$) calcd for $C_{15}H_{10}NaO_3$ $[M+Na]^+$ 261.0522, found 261.0525. The NMR spectra are in accordance with literature.[17]

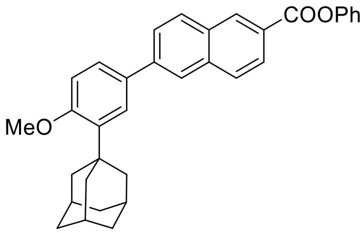

Phenyl 6-(3-(1-adamantyl)-4-methoxyl phenyl)-2-naphthoate (4t)

According to the general procedure for ester synthesis, **4t** was obtained as a white solid. ^{1}H NMR (400 MHz, $CDCl_3$): δ = 1.81 (s, 6H), 2.12 (s, 3H), 2.20 (s, 6 H), 3.92 (s, 3H), 7.01 (d, J = 8.4 Hz, 1H), 7.28 – 7.31 (m, 3H), 7.45 – 7.49 (m, 2H), 7.57 (dd, J = 8.0 Hz, 2.0 Hz, 1H), 7.63 (d, J = 2.4 Hz, 1H), 7.84 (dd, J = 8.4 Hz, 1.6 Hz, 1H), 7.99 (d, J = 8.8 Hz, 1H), 8.05 (d, J = 9.6 Hz, 2H), 8.21 (dd, J = 8.8 Hz, 2.0 Hz, 1H), 8.80 (s, 1H) ppm. ^{13}C NMR (100 MHz, $CDCl_3$): δ = 29.2, 37.2, 37.4, 40.7, 55.3, 112.2, 121.9, 124.9, 125.9, 125.95, 126.03, 126.1, 126.3, 126.8, 128.6, 129.7, 130.0, 131.4, 131.8, 132.5, 136.4, 139.1, 141.9, 151.2, 159.1, 165.6 ppm. HRMS (ESI$^+$) calcd for $C_{34}H_{32}NaO_3$ $[M+Na]^+$ 511.2244, found 511.2243. IR (KBr): 2904, 2850, 1730, 1622, 1474, 1276, 1078, 814, 740, 691 cm^{-1}.

VI. Characterization data of methylated products

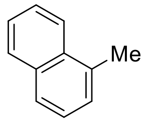

1-Methylnaphthalene (2a)

From nitro group: According to the general methylation method of nitrobenzene, **2a** was obtained as colorless liquid starting from **1a** (23 mg, 80% yield).

From amide: According to the general methylation procedure of amide, **2a** was obtained as colorless liquid starting from **3a** (24 mg, 85% yield).

From ester: According to the general methylation procedure of ester, **2a** was obtained as colorless liquid starting from **4a** (21 mg, 72% yield).

^{1}H NMR (400 MHz, $CDCl_3$): δ = 2.72 (s, 3H), 7.34 (d, J = 6.8 Hz, 1H), 7.36 – 7.42 (m, 1H), 7.48 – 7.57 (m, 2H), 7.73 (d, J = 8.0 Hz, 1H), 7.83 – 7.89 (m, 1H), 8.02 (dd, J = 8.4, 1.2 Hz, 1H) ppm. ^{13}C NMR (100 MHz, $CDCl_3$): δ = 19.5, 124.2, 125.67, 125.70, 125.8, 126.5, 126.7, 128.6, 132.7, 133.7, 134.4 ppm. The NMR spectra are in accordance with literature.[18]

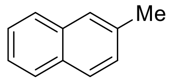

2- Methylnaphthalene (2b)

From nitro group: According to the general methylation method of nitrobenzene, **2b** was obtained as colorless liquid starting from **1b** (19 mg, 68% yield).

From amide: According to the general methylation procedure of amide, **2b** was obtained as colorless liquid starting from **3b** (20 mg, 70% yield).

From ester: According to the general methylation procedure of ester, **2b** was obtained as colorless liquid starting from **4b** (21 mg, 75% yield).

^{1}H NMR (400 MHz, $CDCl_3$): δ = 2.53 (s, 3H), 7.33 (dd, J = 8.4 Hz, 1.6 Hz, 1H), 7.39 – 7.47 (m, 2H), 7.62 (s, 1H), 7.74 – 7.78 (m, 2H), 7.81 (d, J = 7.6 Hz, 1H) ppm. ^{13}C NMR (100 MHz, $CDCl_3$): δ = 21.9, 125.1, 126.0, 127.0, 127.4, 127.7, 127.8, 128.2, 131.8, 133.8, 135.6 ppm. The NMR spectra are in accordance with literature.[18]

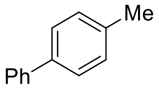

4-Methyl-1,1'-biphenyl (2c)

From nitro group: According to the general methylation method of nitrobenzene, **2c** was obtained as a white solid starting from **1c** (22 mg, 65% yield).

From amide:_According to the general methylation procedure of amide, **2c** was obtained as a white solid starting from **3c** (28 mg, 82% yield).

From ester: According to the general methylation procedure of ester, **2c** was obtained as a white solid starting from **4c** (22 mg, 66% yield).

^{1}H NMR (400 MHz, $CDCl_3$): δ = 2.40 (s, 3H), 7.27 (d, J = 8.4 Hz, 2H), 7.30 – 7.35 (m, 1H), 7.40 – 7.45 (m, 2H), 7.49 – 7.52 (m, 2H), 7.57 – 7.60 (m, 2H) ppm. ^{13}C NMR (100 MHz, $CDCl_3$): δ = 21.2, 127.10, 127.11, 127.13, 128.8, 129.6, 137.2, 138.5, 141.3 ppm. The NMR spectra are in accordance with literature.[18]

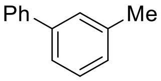

3-Methyl-1,1'-biphenyl (2d)

From nitro group: According to the general methylation method of nitrobenzene, **2d** was obtained as yellowish liquid starting from **1d** (25 mg, 74% yield)

From amide: According to the general methylation procedure of amide, **2d** was obtained as yellowish liquid starting from **3d** (23 mg, 68% yield)

From ester: According to the general methylation procedure of ester, **2d** was obtained as yellowish liquid starting from **4d** (25 mg, 76% yield).

^{1}H NMR (400 MHz, $CDCl_3$): δ = 2.45 (s, 3H), 7.18 – 7.21 (m, 1H), 7.34 – 7.38 (m, 2H), 7.41 – 7.48 (m, 4H), 7.60 – 7.63 (m, 2H) ppm. ^{13}C NMR (100 MHz, $CDCl_3$): δ = 21.7, 124.4, 127.29, 127.31, 128.11, 128.12, 128.79, 128.82, 138.4, 141.4, 141.5 ppm. The NMR spectra are in accordance with literature.[18]

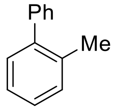

2-Methyl-1,1'-biphenyl (2e)

From nitro group: According to the general methylation method of nitrobenzene, **2e** was obtained as yellowish liquid starting from **1e** (18 mg, 53% yield)

From amide: According to the general methylation procedure of amide, **2e** was obtained as yellowish liquid starting from **3e** (20 mg, 60% yield)

From ester: According to the general methylation procedure of ester, **2e** was obtained as yellowish liquid starting from **4e** (17 mg, 52% yield).

^{1}H NMR (400 MHz, $CDCl_3$): δ = 2.28 (s, 3H), 7.22 – 7.25 (m, 2H), 7.26 – 7.28 (m, 1H), 7.30 – 7.37 (m, 3H), 7.39 – 7.45 (m, 2H), 7.45 – 7.62 (m, 1H) ppm. ^{13}C NMR (100 MHz, $CDCl_3$): δ = 20.6, 125.9, 126.9, 127.3, 127.4, 128.2, 128.9, 129.3, 129.9, 130.4, 135.5 ppm. The NMR spectra are in accordance with literature.[18]

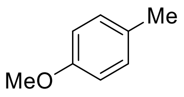

4-Methylanisole (2f)

From nitrobenzene: The methylation of nitrobenzene **1f** was conducted at 130 °C. Diphenylacetylene (35.6 mg, 0.2 mmol) was subjected as internal standard after the completion of reaction. The yield of **2f** was determined by GC analysis using calibration curves based on data from the authentic sample of **2f** and diphenylacetylene (84% yield).

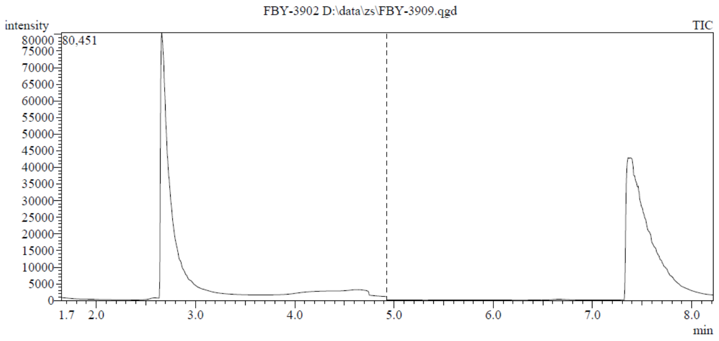

	Compound	RTime	m/z	Area	Height	Conc.
1	p-methylanisole	2.662	122.00	280492	42329	0.838
2	diphenylacetylene	7.381	177.90	471329	31828	1.000

Calibration Curve

ID#:1 m/z:122.00 Name:p-methylanisole
f(x)=0.709868*x+0.000000
rr1=0.999478 rr2=0.998957
MeanRF:0.68 RFSD:0.06 RFRSD:8.97
CurveType:Least square
ZeroThrough:Yes
WeightedRegression:No
Internal Standard

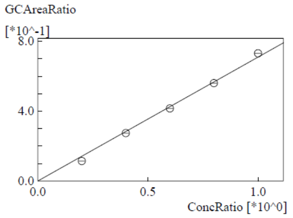

#	ConcRatio	GCAreaRatio	GCArea of IntStandard
1	1.000	0.73	384885.00
2	0.800	0.56	280771.00
3	0.600	0.42	196416.00
4	0.400	0.27	113751.00
5	0.200	0.11	47479.00

From amide: According to the general methylation method of amide, the decarbonylative methylation of amide **3f** was conducted with 2 equiv Et_3N added. Diphenylacetylene (35.6 mg, 0.2 mmol) was subjected as internal standard after the completion of reaction. The yields of **2f** was determined by GC analysis using calibration curves based on data from the authentic sample of **2f** and diphenylacetylene (47% yield).

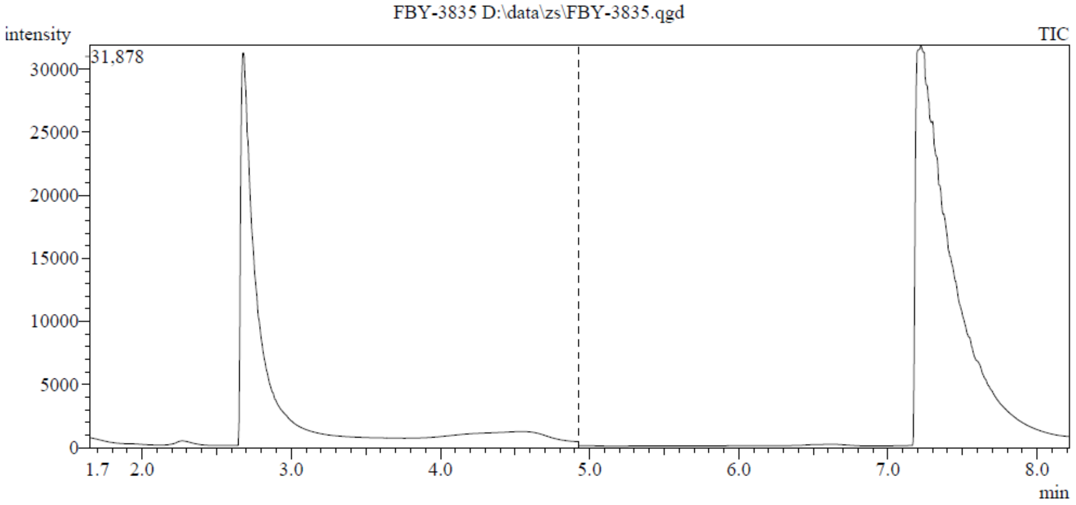

	Compound	RTime	m/z	Area	Height	Conc.
1	p-methylanisole	2.681	122.00	119880	16536	0.472
2	diphenylacetylene	7.222	177.90	357542	23431	1.000

Calibration Curve

ID#:1 m/z:122.00 Name:p-methylanisole
f(x)=0.709868*x+0.000000
rr1=0.999478 rr2=0.998957
MeanRF:0.68 RFSD:0.06 RFRSD:8.97
CurveType:Least square
ZeroThrough:Yes
WeightedRegression:No
Internal Standard

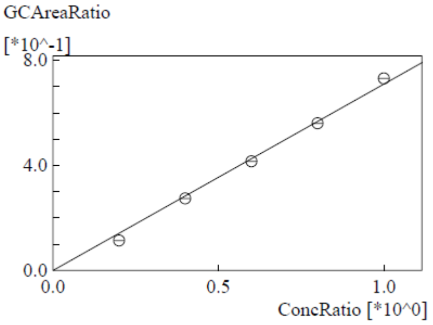

#	ConcRatio	GCAreaRatio	GCArea of IntStandard
1	1.000	0.73	384885.00
2	0.800	0.56	280771.00
3	0.600	0.42	196416.00
4	0.400	0.27	113751.00
5	0.200	0.11	47479.00

From ester: According to the general methylation procedure of ester starting from **4f**, diphenylacetylene (35.6 mg, 0.2 mmol) was subjected as internal standard after the completion of reaction. The yield of **2f** were determined by GC analysis using calibration curves based on data from the authentic sample of **2f** and diphenylacetylene (13% yield).

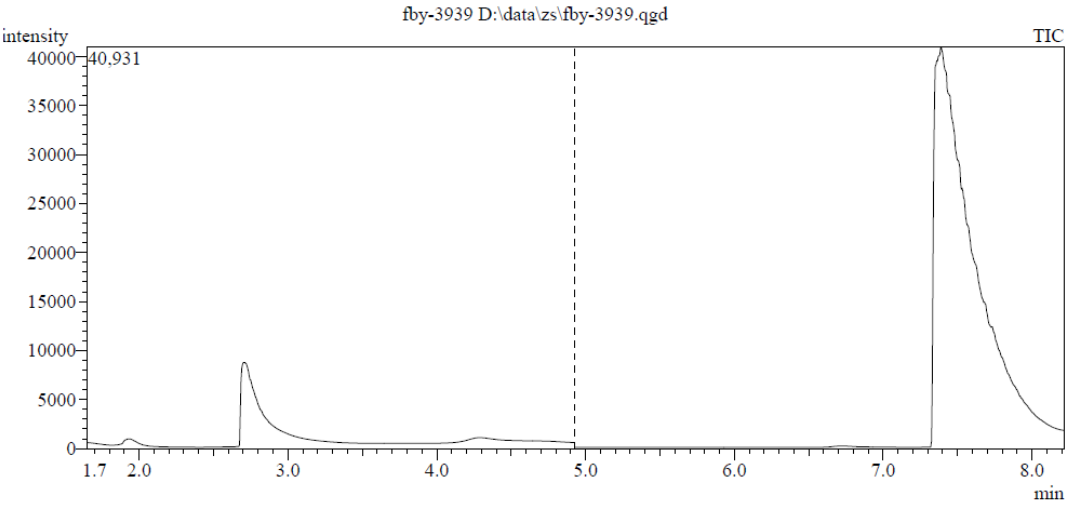

	Compound	RTime	m/z	Area	Height	Conc.
1	p-methylanisole	2.709	122.00	47258	4846	0.128
2	diphenylacetylene	7.390	177.90	521541	30652	1.000

Calibration Curve

ID#:1 m/z:122.00 Name:p-methylanisole
f(x)=0.709868*x+0.000000
rr1=0.999478 rr2=0.998957
MeanRF:0.68 RFSD:0.06 RFRSD:8.97
CurveType:Least square
ZeroThrough:Yes
WeightedRegression:No
Internal Standard

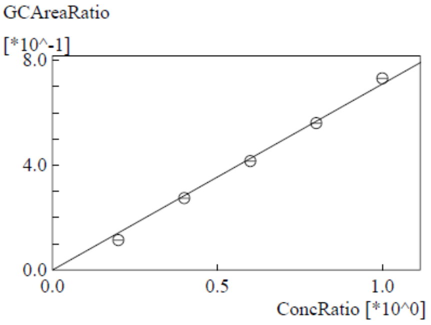

#	ConcRatio	GCAreaRatio	GCArea of IntStandard
1	1.000	0.73	384885.00
2	0.800	0.56	280771.00
3	0.600	0.42	196416.00
4	0.400	0.27	113751.00
5	0.200	0.11	47479.00

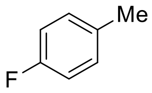

1-Fluoro-4-methylbenzene (2g)

From nitro group: According to the general methylation method of nitrobenzene starting from **1g**, mesitylene (27.8 μL, 0.2 mmol) was subjected as internal standard after the completion of reaction. The yield of **2g** was determined by GC analysis using calibration curves based on data from the authentic sample of **2g** and mesitylene (46% yield).

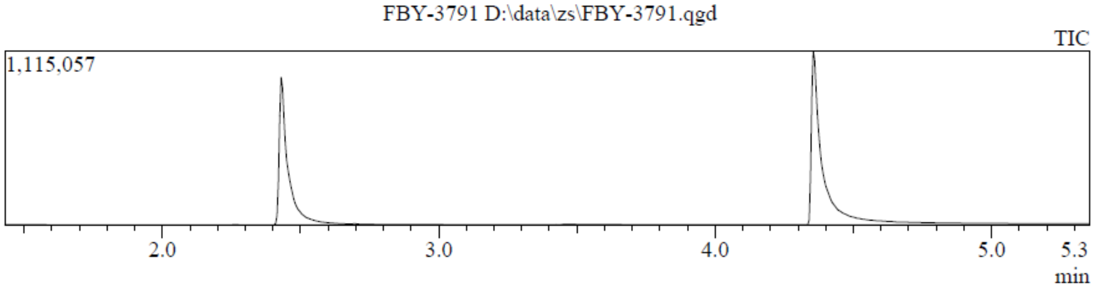

IDName	RTime	m/z	Area	Height	Conc.
1 Benzene, 1-fluoro-4-methyl-	2.432	109.00	1275774	565452	0.464
2 mes	4.360	120.00	795469	320903	1.000

Calibration Curve

ID#:1 m/z:109.00 Name:Benzene, 1-fluoro-4-methyl-
f(x)=3.457415*x+0.000000
rr1=0.998830 rr2=0.997661
MeanRF:3.66 RFSD:0.30 RFRSD:8.21
CurveType:Least square
ZeroThrough:Yes
WeightedRegression:No
Internal Standard

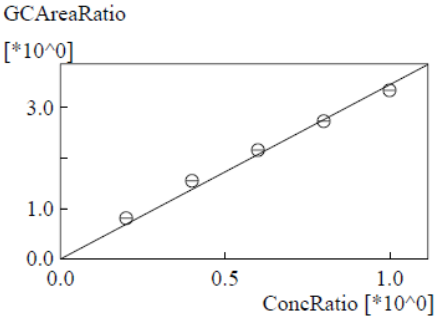

#	ConcRatio	GCAreaRatio	GCArea of IntStandard
1	0.200	0.81	233602.00
2	0.400	1.55	519851.00
3	0.600	2.16	939737.00
4	0.800	2.73	1332779.00
5	1.000	3.34	1708343.00

From amide: According to the general methylation procedure of amide starting from **3g**, mesitylene (27.8 µL, 0.2 mmol) was subjected as internal standard after the completion of reaction. The yield of **2g** was determined by GC analysis using calibration curves based on data from the authentic sample of **2g** and mesitylene (47% yield).

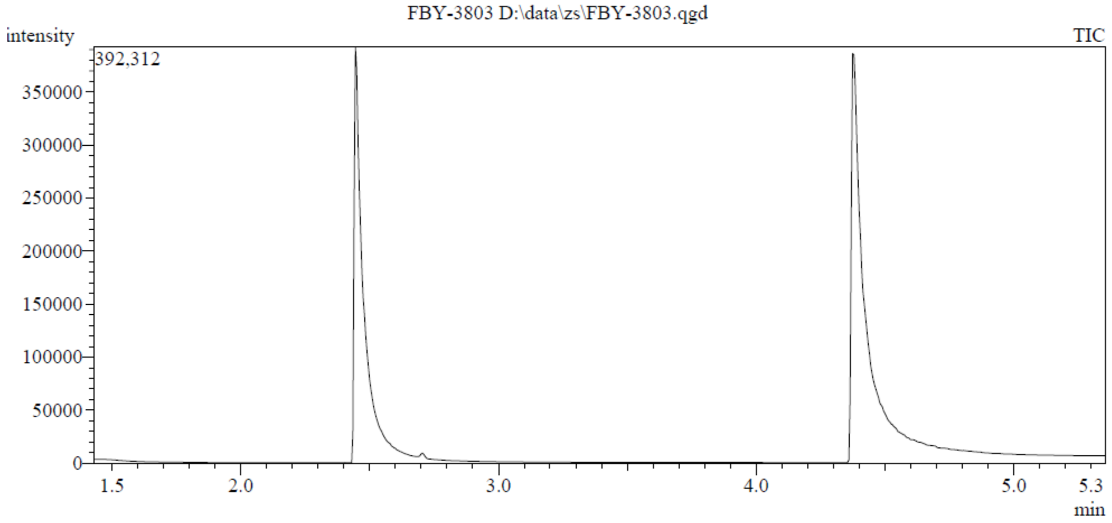

	Compound	RTime	m/z	Area	Height	Conc.
1	Benzene, 1-fluoro-4-methyl-	2.447	109.00	621320	229856	0.470
2	mes	4.382	120.00	382036	111524	1.000

Calibration Curve

ID#:1 m/z:109.00 Name:Benzene, 1-fluoro-4-methyl-
f(x)=3.457415*x+0.000000
rr1=0.998830 rr2=0.997661
MeanRF:3.66 RFSD:0.30 RFRSD:8.21
CurveType:Least square
ZeroThrough:Yes
WeightedRegression:No
Internal Standard

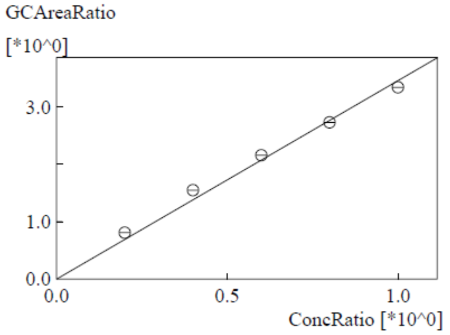

#	ConcRatio	GCAreaRatio	GCArea of IntStandard
1	0.200	0.81	233602.00
2	0.400	1.55	519851.00
3	0.600	2.16	939737.00
4	0.800	2.73	1332779.00
5	1.000	3.34	1708343.00

From ester: According to the general methylation procedure of ester starting from **4g**, mesitylene (27.8 µL, 0.2 mmol) was subjected as internal standard after the completion of reaction. The yield of **2g** was determined by GC analysis using calibration curves based on data from the authentic sample of **2g** and mesitylene (68% yield).

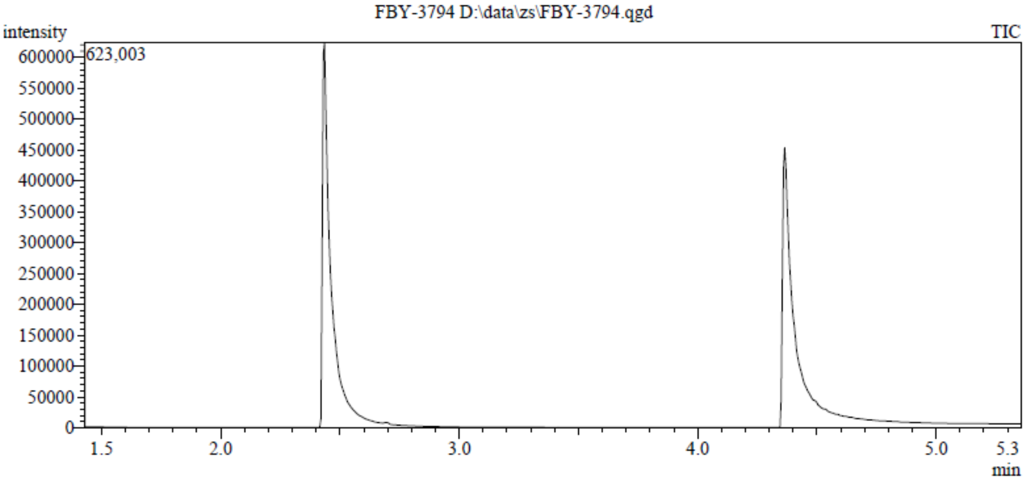

	Compound	RTime	m/z	Area	Height	Conc.
1	Benzene, 1-fluoro-4-methyl-	2.434	109.00	956819	382234	0.684
2	mes	4.368	120.00	404443	126630	1.000

Calibration Curve

ID#:1 m/z:109.00 Name:Benzene, 1-fluoro-4-methyl-
f(x)=3.457415*x+0.000000
rr1=0.998830 rr2=0.997661
MeanRF:3.66 RFSD:0.30 RFRSD:8.21
CurveType:Least square
ZeroThrough:Yes
WeightedRegression:No
Internal Standard

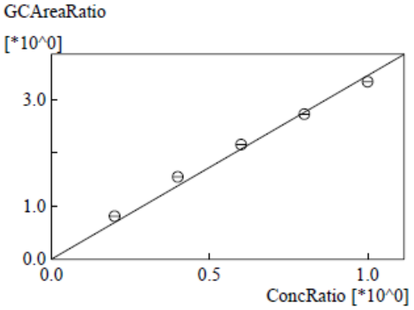

#	ConcRatio	GCAreaRatio	GCArea of IntStandard
1	0.200	0.81	233602.00
2	0.400	1.55	519851.00
3	0.600	2.16	939737.00
4	0.800	2.73	1332779.00
5	1.000	3.34	1708343.00

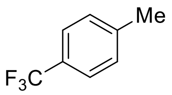

4-Methylbenzotrifluoride (2h)

From nitro group: According to the general methylation method of nitrobenzene starting from **1h**, mesitylene (27.8 μL, 0.2 mmol) was subjected as internal standard after the completion of reaction. The yield of **2h** was determined by GC analysis using calibration curves based on data from the authentic sample of **2h** and mesitylene (62% yield).

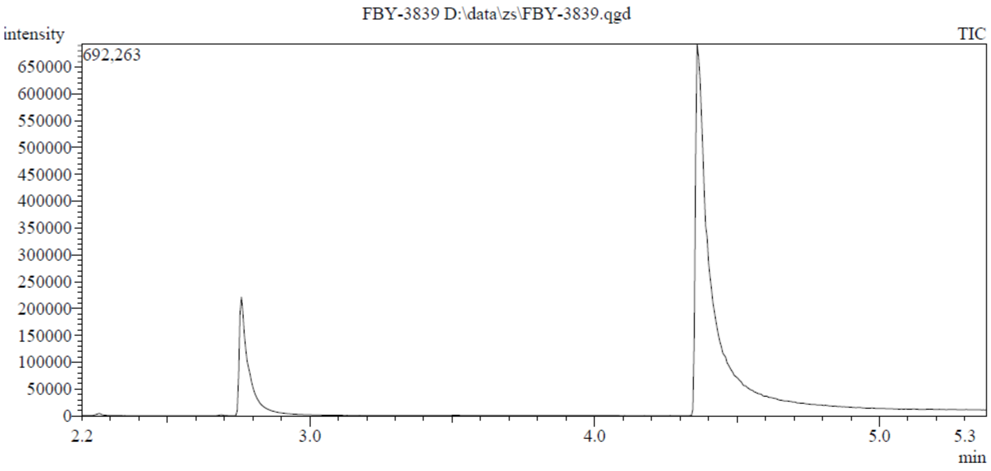

	Compound	RTime	m/z	Area	Height	Conc.
1	4-(trifluoromethyl)-toluene	2.761	159.90	298889	135344	0.623
2	Mes	4.364	120.00	679720	193130	1.000

Calibration Curve

ID#:1 m/z:159.90 Name:4-(trifluoromethyl)-toluene
f(x)=0.705366*x+0.000000
rr1=0.986979 rr2=0.974128
MeanRF:0.68 RFSD:0.07 RFRSD:9.64
CurveType:Least square
ZeroThrough:Yes
WeightedRegression:No
Internal Standard

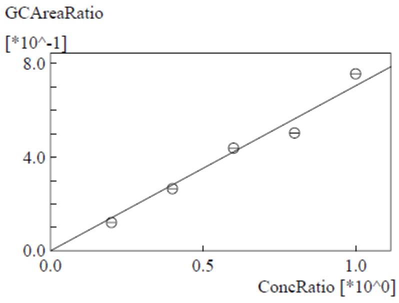

#	ConcRatio	GCAreaRatio	GCArea of IntStandard
1	1.000	0.76	888122.00
2	0.800	0.50	636673.00
3	0.600	0.44	467292.00
4	0.400	0.27	306275.00
5	0.200	0.12	123823.00

From amide: According to the general methylation method of amide, the decarbonylative methylation of amide **3h** was conducted with 2 equiv Et_3N added. Mesitylene (27.8 μL, 0.2 mmol) was subjected as internal standard after the completion of reaction. The yield of **2h** was determined by GC analysis using calibration curves based on data from the authentic sample of **2h** and mesitylene (55% yield).

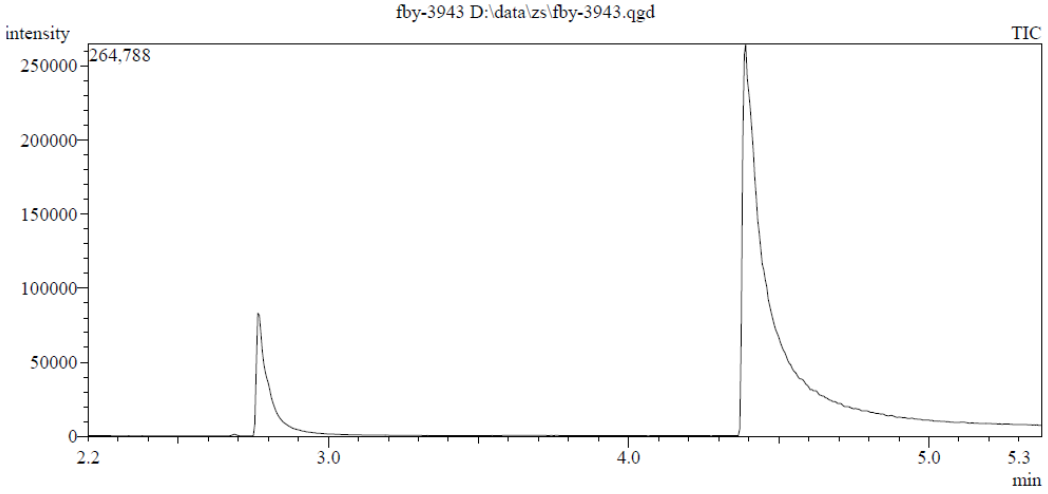

	Compound	RTime	m/z	Area	Height	Conc.
1	4-(trifluoromethyl)-toluene	2.768	159.90	135913	52471	0.546
2	Mes	4.390	120.00	352973	78791	1.000

Calibration Curve

ID#:1 m/z:159.90 Name:4-(trifluoromethyl)-toluene
f(x)=0.705366*x+0.000000
rr1=0.986979 rr2=0.974128
MeanRF:0.68 RFSD:0.07 RFRSD:9.64
CurveType:Least square
ZeroThrough:Yes
WeightedRegression:No
Internal Standard

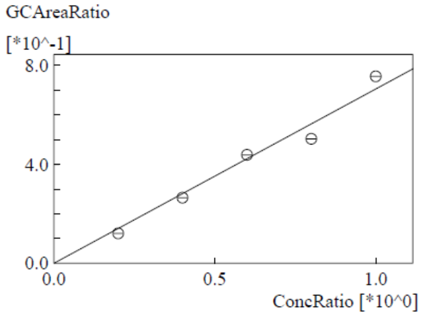

#	ConcRatio	GCAreaRatio	GCArea of IntStandard
1	1.000	0.76	888122.00
2	0.800	0.50	636673.00
3	0.600	0.44	467292.00
4	0.400	0.27	306275.00
5	0.200	0.12	123823.00

From ester: According to the general methylation procedure of ester starting from **4h**, mesitylene (27.8 μL, 0.2 mmol) was subjected as internal standard after the completion of reaction. The yield of **2h** was determined by GC analysis using calibration curves based on data from the authentic sample of **2h** and mesitylene (39% yield).

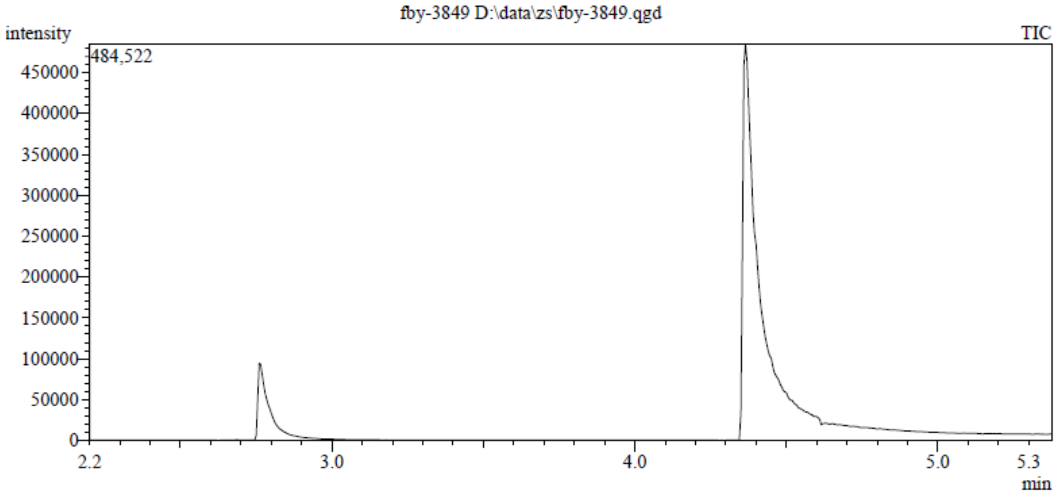

	Compound	RTime	m/z	Area	Height	Conc.
1	4-(trifluoromethyl)-toluene	2.763	159.90	140068	56099	0.393
2	Mes	4.367	120.00	505485	135348	1.000

Calibration Curve

ID#:1 m/z:159.90 Name:4-(trifluoromethyl)-toluene
f(x)=0.705366*x+0.000000
rr1=0.986979 rr2=0.974128
MeanRF:0.68 RFSD:0.07 RFRSD:9.64
CurveType:Least square
ZeroThrough:Yes
WeightedRegression:No
Internal Standard

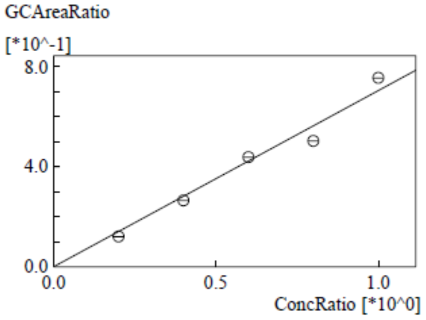

#	ConcRatio	GCAreaRatio	GCArea of IntStandard
1	1.000	0.76	888122.00
2	0.800	0.50	636673.00
3	0.600	0.44	467292.00
4	0.400	0.27	306275.00
5	0.200	0.12	123823.00

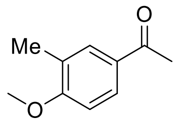

1-(4-Methoxy-3-methylphenyl)ethan-1-one (2i)

From nitro group: According to the general methylation method of nitrobenzene, **2i** was obtained as colorless liquid starting from **1i** (26 mg, 78% yield). ^{1}H NMR (400 MHz, $CDCl_3$): δ = 2.24 (s, 3H), 2.54 (s, 3H), 3.89 (s, 3H), 6.84 (d, J = 8.4 Hz, 1H), 7.62 – 7.77 (m, 1H), 7.80 – 7.83 (m, 1H) ppm. ^{13}C NMR (100 MHz, $CDCl_3$): δ = 16.4, 26.5, 55.6, 109.3, 126.9, 128.6, 129.9, 131.0, 161.9, 197.3 ppm. The NMR spectra are in accordance with literature.[19]

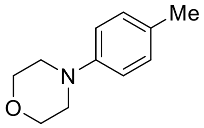

4-(*p*-Tolyl)morpholine (2j)

From nitro group: According to the general methylation method of nitrobenzene, **2j** was obtained as a white solid starting from **1j** (14 mg, 40% yield). ^{1}H NMR (400 MHz, $CDCl_3$): δ = 2.28 (s, 3H), 3.10 – 3.14 (m, 4H), 3.85 – 3.88 (m, 4H), 6.84 (d, J = 8.4 Hz, 2H), 7.10 (d, J = 8.4 Hz, 2H) ppm. ^{13}C NMR (100 MHz, $CDCl_3$): δ = 20.6, 50.1, 67.1, 116.2, 129.7, 129.8, 149.3 ppm. The NMR spectra are in accordance with literature.[20]

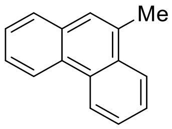

9-Methylphenanthrene (2k)

From nitro group: According to the general methylation method of nitrobenzene, **2k** was obtained as a white solid starting from **1k** (32 mg, 82% yield). ^{1}H NMR (400 MHz, $CDCl_3$): δ = 2.76 (s, 3H), 7.55 – 7.64 (m, 3H), 7.64 – 7.71 (m, 2H), 7.83 (dd, J = 6.4, 1.6 Hz, 1H), 8.06 – 8.11 (m, 1H), 8.67 (d, J = 7.2 Hz, 1H), 8.71 – 8.78 (m, 1H) ppm. ^{13}C NMR (100 MHz, $CDCl_3$): δ = 20.2, 122.6, 123.1, 124.8, 125.9, 126.3, 126.65, 126.72, 126.9, 128.0, 129.8, 130.5, 132.1, 132.2, 132.6 ppm. The NMR spectra are in accordance with literature.[21]

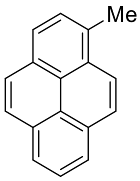

1-Methylpyrene (2l)

From nitro group: According to the general methylation method of nitrobenzene, **2l** was obtained as a white solid starting from **1l** (33 mg, 76% yield). ^{1}H NMR (400 MHz, $CDCl_3$): δ = 3.00 (s, 3H), 7.85 – 7.90 (m, 1H), 7.98 – 8.07 (m, 3H), 8.08 – 8.14 (m, 2H), 8.16 – 8.22 (m, 2H), 8.25 (d, J = 9.2 Hz, 1H) ppm. ^{13}C NMR (100 MHz, $CDCl_3$): δ = 20.0, 123.8, 124.8, 124.87, 124.92, 124.93, 124.95, 125.9, 126.5, 127.2, 127.7, 128.0, 129.3, 129.8, 131.1, 131.5, 132.4 ppm. The NMR spectra are in accordance with literature.[21]

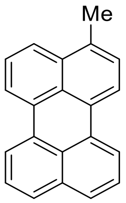

3-Methylperylene (2m)

From nitro group: According to the general methylation method of nitrobenzene, **2m** was obtained as a pale yellow solid starting from **1m** (27 mg, 51% yield). ^{1}H NMR (400 MHz, $CDCl_3$): δ = 2.66 (s, 3H), 7.34 (d, J = 8.4 Hz, 1H), 7.44 – 7.56 (m, 3H), 7.67 (t, J = 8.0 Hz, 2H), 7.83 (d, J = 8.4 Hz, 1H), 8.10 (d, J = 7.6 Hz, 1H), 8.13 – 8.24 (m, 3H) ppm. ^{13}C NMR (100 MHz, $CDCl_3$): δ = 20.0, 119.8, 120.1, 120.27, 120.29, 124.2, 126.4, 126.65, 126.71, 127.4, 127.6, 127.9, 128.6, 128.9, 129.6, 131.5, 131.6, 131.7, 133.8, 134.4, 134.8 ppm. IR (KBr): 3044, 2943, 2898, 2856, 1500, 1387, 1186, 817, 762 cm^{-1}.

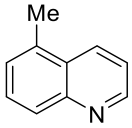

5-Methylquinoline (2n)

From nitro group: According to the general methylation method of nitrobenzene, **2n** was obtained as yellow liquid starting from **1n** (24 mg, 84% yield). ^{1}H NMR (400 MHz, $CDCl_3$): δ = 2.68 (s, 3H), 7.37 (d, J = 7.2 Hz, 1H), 7.40 – 7.45 (m, 1H), 7.58 – 7.62 (m, 1H), 7.96 (d, J = 8.4 Hz, 1H), 8.31 – 8.33 (m, 1H), 8.91 (d, J = 4.0 Hz, 1H) ppm. ^{13}C NMR (100 MHz, $CDCl_3$): δ = 18.7, 120.8, 127.1,

127.7, 127.8, 129.3, 132.6, 134.7, 148.6, 150.0 ppm. The NMR spectra are in accordance with literature.[22]

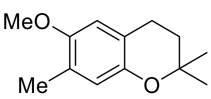

6-Methoxy-2,2,7-trimethylchromane (2o)

From nitro group: According to the general methylation method of nitrobenzene, **2o** was obtained as a white solid starting from **1o** (19 mg, 45% yield). ^{1}H NMR (400 MHz, $CDCl_3$): δ = 1.31 (s, 6H), 1.77 (t, *J* = 6.8 Hz, 2H), 2.16 (s, 3H), 2.75 (t, *J* = 6.8 Hz, 2H), 3.74 (s, 3H), 6.46 (d, *J* = 3.6 Hz, 1H), 6.56 – 6.59 (m, 1H) ppm. ^{13}C NMR (400 MHz, $CDCl_3$): δ = 16.4, 23.2, 26.9, 27.1, 33.0, 55.8, 73.6, 111.1, 114.8, 120.8, 127.3, 146.3, 152.2 ppm. HRMS (ESI$^+$) calcd for $C_{13}H_{19}NO_2$ $[M+H]^+$ 207.1380, found 207.1378. IR (KBr): 2954, 2922, 2850, 1479, 1457, 1423, 1250, 1203, 1049, 1020, 798, 711 cm^{-1}.

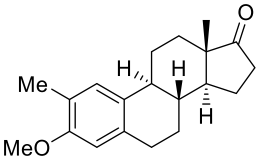

2-Methylestrone-3-methyl ether (2p)

From nitro group: According to the general methylation method of nitrobenzene, **2p** was obtained as yellow liquid starting from **1p** (37 mg, 62%). ^{1}H NMR (400 MHz, $CDCl_3$): δ = 0.91 (s, 3H), 1.38 – 1.46 (m, 1H), 1.47 – 1.52 (m, 2H), 1.52 – 1.56 (m, 1H), 1.59 (d, *J* = 9.4 Hz, 1H), 1.61 – 1.66 (m, 1H), 1.92 – 2.18 (m, 4H), 2.19 (s, 3H), 2.21 – 2.29 (m, 1H), 2.38 – 2.46 (m, 1H), 2.46 – 2.56 (m, 1H), 2.83 – 2.95 (m, 2H), 3.80 (d, *J* = 1.2 Hz, 3H), 6.57 (s, 1H), 7.07 (s, 1H) ppm. ^{13}C NMR (400 MHz, $CDCl_3$): δ = 14.0, 16.2, 21.7, 26.1, 26.8, 29.7, 31.7, 36.0, 38.6, 44.1, 48.2, 50.5, 55.4, 110.5, 124.0, 127.8, 131.3, 134.8, 155.9, 221.2 ppm. HRMS (ESI$^+$) calcd for $C_{20}H_{27}O_2$ $[M+H]^+$ 299.2006, found 299.2014. IR (KBr): 2994, 2874, 1739, 1612, 1508, 1256, 1214, 1098, 1053, 1024, 890, 830 cm^{-1}.

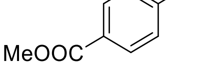

Methyl 4-methylbenzoate (2q)

From amide: According to the general methylation procedure of amide, **2q** was obtained as a white solid starting from **3q** (23 mg, 78% yield).

From ester: According to the general methylation procedure of ester, **2q** was obtained as a white solid starting from **4q** (23 mg, 80% yield).

^{1}H NMR (400 MHz, $CDCl_3$): δ = 2.40 (s, 3H), 3.90 (s, 3H), 7.23 (d, J = 8.0 Hz, 2H), 7.93 (d, J = 8.4 Hz, 2H) ppm. ^{13}C NMR (100 MHz, $CDCl_3$): δ = 21.8, 52.1, 127.5, 129.2, 129.7, 143.7, 167.3 ppm. The NMR spectra are in accordance with literature.[23]

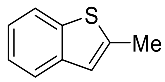

2-Methylbenzo[*b*]thiophene (2r)

From amide: According to the general methylation procedure of amide, **2r** was obtained as a white solid starting from **3r** (23 mg, 76% yield).

From ester: According to the general methylation procedure of ester, **2r** was obtained as a white solid starting from **4r** (21 mg, 70% yield).

^{1}H NMR (400 MHz, $CDCl_3$): δ = 2.60 (d, J = 1.2 Hz, 3H), 6.97 – 7.00 (m, 1H), 7.26 (td, J = 7.6, 1.4 Hz, 1H), 7.29 – 7.34 (m, 1H), 7.66 (d, J = 7.2 Hz, 1H), 7.76 (dd, J = 8.0, 1.2 Hz, 1H) ppm. ^{13}C NMR (100 MHz, $CDCl_3$): δ = 16.3, 121.7, 122.1, 122.6, 123.5, 124.2, 139.8, 140.6, 141.0 ppm. The NMR spectra are in accordance with literature.[24]

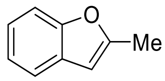

2-Methylbenzofuran (2s)

From amide: According to the general methylation procedure of amide, **2s** was obtained as colorless liquid starting from **3s** (14 mg, 54% yield).

From ester: According to the general methylation procedure of ester, **2s** was obtained as colorless liquid starting from **4s** (22 mg, 66% yield).

^{1}H NMR (400 MHz, $CDCl_3$): δ = 2.46 (d, J = 1.2 Hz, 3H), 6.37 (penta, J = 1.0 Hz, 1H), 7.13 – 7.23 (m, 2H), 7.39 – 7.42 (m, 1H), 7.45 – 7.49 (m, 1H) ppm. ^{13}C NMR (100 MHz, $CDCl_3$): δ = 14.2, 102.7, 110.7, 120.2, 122.5, 123.2, 129.3, 154.8, 155.5 ppm. The NMR spectra are in accordance with literature.[24]

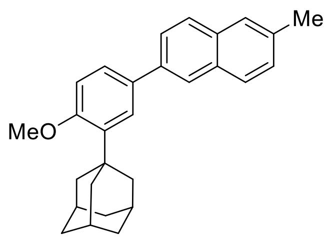

2-Methyl-6-(3-(1-adamantyl)-4-methoxylphenyl)-naphthalene (2t)

From ester: According to the general methylation procedure of ester, **2t** was obtained as a white solid starting from **4t** (53 mg, 69% yield). ^{1}H NMR (400 MHz, $CDCl_3$): δ = 1.83 (s, 6H), 2.13 (s, 3H), 2.21 (s, 6H), 2.54 (s, 3H), 3.91 (s, 3H), 7.00 (d, J = 8.4 Hz, 1H), 7.34 (dd, J = 8.4, 1.3 Hz, 1H), 7.54 (dd, J = 8.4, 2.3 Hz, 1H), 7.60 – 7.65 (m, 2H), 7.71 (dd, J = 8.5, 1.6 Hz, 1H), 7.79 – 7.84 (m, 2H), 7.96 (s, 1H) ppm. ^{13}C NMR (100 MHz, $CDCl_3$): δ = 21.9, 29.3, 37.3, 37.3, 40.7, 55.3, 112.2, 124.9, 125.6, 125.8, 126.0, 126.7, 127.7, 128.0, 128.6, 132.1, 132.6, 133.4, 135.3, 138.2, 138.9, 158.5 ppm. HRMS (ESI$^+$) calcd for $C_{28}H_{31}O$ $[M+H]^+$ 383.2369, found 383.2377. IR (KBr): 2956, 2905, 2850, 1602, 1498, 1460, 1235, 1140, 1032, 877, 807 cm^{-1}.

VII. Sequential methylation of 1-fluoro-4-nitronaphthalene.

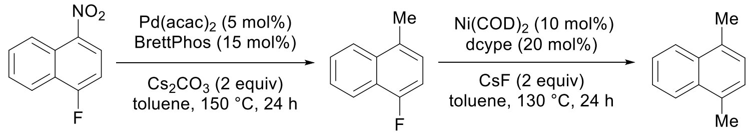

8 **9**, 76% **10**, 55%

1-Fluoro-4-nitronaphthalene (**8**) was prepared according to the literature.[25]

An oven-dried vial equipped with a stirring bar was charged with **8** (76.4 mg, 0.4 mmol), TMB (200 µL, 3.5*N* in THF, 1.75 equiv), $Pd(acac)_2$ (6.2 mg, 5 mol%), BrettPhos (32.2 mg, 15 mol%), Cs_2CO_3 (260 mg, 2 equiv) and toluene (1.2 mL) under N_2 at room temperature. The reaction mixture was placed in a preheated oil bath at 150 °C, and stirred for 24 h. Then the reaction mixture was cooled down to room temperature, diluted with CH_2Cl_2 (20 mL), filtered through celite, and concentrated under reduced pressure. Purification by column chromatography on silica gel (200-300 mesh) afforded the corresponding product **9** as colorless oil (49 mg, 76% yield). ^{1}H NMR (400 MHz, $CDCl_3$): δ = 2.65 (s, 3H), 6.98 – 7.08 (m, 1H), 7.18 – 7.25 (m, 1H), 7.52 – 7.62 (m, 2H), 7.95 – 8.01 (m, 1H), 8.10 – 8.18 (m, 1H) ppm. ^{13}C NMR (100 MHz, $CDCl_3$): δ = 19.1, 108.92 (d, J = 19.5 Hz), 121.15 (d, J = 5.6 Hz), 123.85 (d, J = 16.4 Hz), 124.34 (d, J = 2.7 Hz), 125.9, 125.97 (d, J = 5.6 Hz), 126.74 (d, J = 1.0 Hz), 130.06 (d, J = 4.8 Hz), 133.68 (d, J = 4.4 Hz), 157.64 (d, J = 249.1 Hz) ppm. ^{19}F NMR (376 MHz, $CDCl_3$): δ = -126.50 ppm. HRMS (ESI$^+$) calcd for $C_{11}H_9FNa$ $[M+Na]^+$ 183.0580, found 183.0581. IR (KBr): 2927, 2866, 1601, 1466, 1397, 1225, 1050, 819, 760, 731 cm^{-1}.

An oven-dried vial equipped with a stirring bar was charged with **9** (32 mg, 0.2 mmol), TMB (100 µL, 3.5*N* in THF, 1.75 equiv), $Ni(COD)_2$ (5.5 mg, 10 mol%), dcype (17 mg, 20 mol%), CsF (61mg, 2.0 equiv) and toluene (0.6 mL) under N_2 at room temperature. The reaction mixture was placed in a preheated oil bath at 130 °C, and stirred for 24 h. The reaction mixture was then cooled down to room temperature, diluted with CH_2Cl_2 (10 mL), filtered through celite, and concentrated. Purification by column chromatography on silica gel (200-300 mesh, petroleum ether) afforded the corresponding product **10** colorless oil (17 mg, 55% yield). ^1H NMR (400 MHz, $CDCl_3$): δ = 2.67 (s, 6H), 7.22 (s, 2H), 7.54 (dd, J = 6.4, 3.2 Hz, 2H), 8.02 (dd, J = 6.4, 3.2 Hz, 2H) ppm. ^{13}C NMR (100 MHz, $CDCl_3$): δ = 19.5, 124.8, 125.5, 126.4, 132.5, 132.8 ppm. The NMR spectra are in accordance with literature.[26]

VIII. Synthetic applications of catalytic methylation of unconventional aryl electrophiles

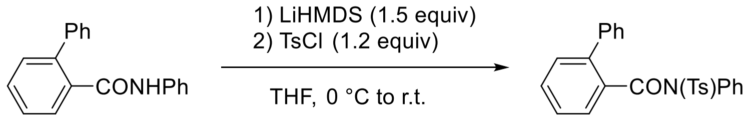

11 **3e**

***N*-phenyl-*N*-tosyl-(1,1'-biphenyl)-2-carboxamide (3e):** *N*-phenyl-(1,1'-biphenyl)-2-carboxamide **11** (137 mg, 0.5 mmol) was dissolved in THF (2.5 mL). LiHMDS (1 mol/L in THF, 0.75 mL, 1.5 equiv) was added slowly at 0 °C under N_2. After stirring at 0 °C for 1 h, TsCl (114 mg, 1.2 equiv) was added slowly. Then the reaction mixture was quenched by water after further stirring at room temperature for 15 h. The mixture was sequentially washed with 1 *N* HCl (2 mL), saturated aqueous $NaHCO_3$ (2 mL), and brine (2 mL). The organic layer was dried over Na_2SO_4, filtered and concentrated under reduced pressure. The resulting crude mixture was purified by column chromatography on silica gel (200-300 mesh) to afford **3e** as a white solid (182 mg, 85% yield).

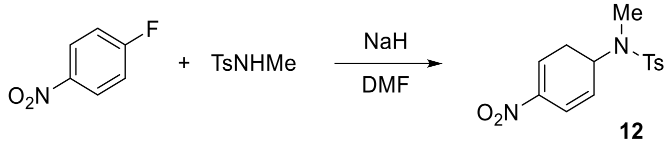

12

***N*,4-dimethyl-*N*-(4-nitrophenyl)benzenesulfonamide** (**12**): An oven-dried vial equipped with a stirring bar was charged with *N*-methyl-*p*-toluenesulfonamide (1.85 g, 10 mmol) and DMF (15 mL) under N_2. NaH (440 mg, 11 mmol) was added in portions at room temperature. The reaction mixture was stirred for 1 h. Then, *p*-fluoronitrobenzene (1.17 mL, 11 mmol) was added and the reaction mixture was stirred for another 1 h. The reaction was then quenched with water (30 mL) and extracted with EtOAc (50 mL×2). Purification by chromatography on silica gel (200-300 mesh,

petroleum ether/ CH_2Cl_2 = 2/1) afforded the corresponding product **12** as a yellowish solid (2.75 g, 90% yield). ^{1}H NMR (400 MHz, $CDCl_3$): δ = 2.42 (s, 3H), 3.22 (s, 3H), 7.24 – 7.28 (m, 2H), 7.31 – 7.35 (m, 2H), 7.39 – 7.44 (m, 2H), 8.14 – 8.20 (m, 2H) ppm. ^{13}C NMR (100 MHz, $CDCl_3$): δ = 21.7, 37.6, 124.4, 125.7, 127.7, 129.9, 132.9, 144.6, 145.7, 147.5 ppm. HRMS (ESI$^+$) calcd for $C_{14}H_{14}N_2NaO_4S$ $[M+Na]^+$ 329.0566, found 329.0566. IR (KBr): 3080, 2900, 1592, 1521, 1492, 1348, 1170, 1054, 872, 721, 665 cm^{-1}.

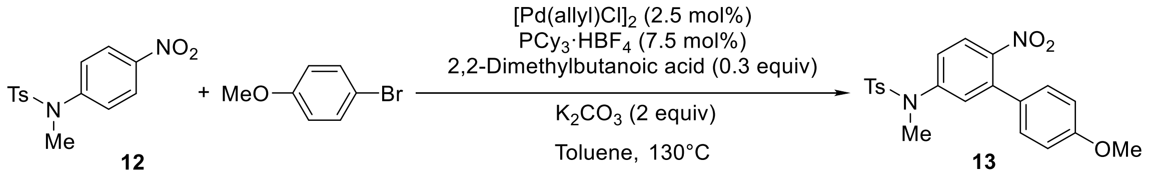

***N*-(4'-methoxy-6-nitro-[1,1'-biphenyl]-3-yl)-*N*,4-dimethylbenzenesulfonamide** (**13**)**:** Compound **13** was synthesized by a modified procedure of literature.[27] An oven-dried vial equipped with a stirring bar was charged with nitroaromatic **12** (4 mmol, 2.0 equiv), 4-bromoanisole (250 µL, 2 mmol, 1.0 equiv), $[Pd(allyl)Cl]_2$ (19 mg, 2.5 mol%), $PCy_3{\cdot}HBF_4$ (55 mg, 7.5 mol%), 2,2-dimethylbutanoic acid (DMBA, 75 µL, 0.3 equiv) and K_2CO_3 (550 mg, 2.0 equiv) under N_2. Toluene (5 mL) was added at room temperature. The reaction mixture was placed in a preheated oil bath at 130 °C, and stirred for 24 h. The reaction mixture was then cooled down to room temperature, diluted with CH_2Cl_2 (50 mL), filtered through celite, and concentrated. Purification by chromatography on silica gel (200-300 mesh, petroleum ether/ CH_2Cl_2/EtOAc = 20/4/1) afforded the corresponding product **13** as a yellowish solid.(594 mg, 72% yield) ^{1}H NMR (400 MHz, $CDCl_3$): δ = 2.43 (s, 3H), 3.20 (s, 3H), 3.84 (s, 3H), 6.91 – 6.96 (m, 2H), 7.15 – 7.22 (m, 4H), 7.29 (d, *J* = 7.9 Hz, 2H), 7.47 (d, *J* = 8.3 Hz, 2H), 7.75 – 7.79 (m, 1H) ppm. ^{13}C NMR (100 MHz, $CDCl_3$): δ = 21.8, 37.7, 55.5, 114.4, 124.4, 125.1, 127.9, 128.9, 128.9, 129.2, 129.8, 133.1, 137.0, 144.5, 145.0, 147.0, 160.0 ppm. HRMS (ESI$^+$) calcd for $C_{21}H_{20}N_2NaO_5S$ $[M+Na]^+$ 435.0985, found 435.0982. IR (KBr): 2926, 2853, 1612, 1515, 1347, 1255, 1169, 901, 746, 663 cm^{-1}.

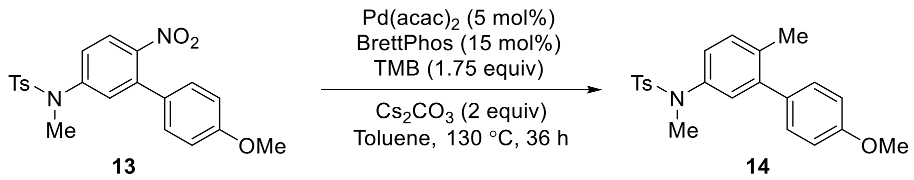

***N*-(4'-methoxy-6-methyl-[1,1'-biphenyl]-3-yl)-*N*,4-dimethylbenzenesulfonamide (14):** Compound **14** was synthesized according to the general methylation procedure of nitro group from **13**. The reaction was conducted at 130 °C for 36 h. The corresponding product **14** was obtained as yellowish oil. (27 mg, 35% yield) ^{1}H NMR (400 MHz, $CDCl_3$) δ = 2.25 (s, 3H), 2.43 (s, 3H), 3.14 (s, 3H), 3.84 (s, 3H), 6.83 (d, *J* = 2.3 Hz, 1H), 6.92 (d, *J* = 8.8 Hz, 2H), 7.02 (dd, *J* = 8.0, 2.4

Hz, 1H), 7.13 (d, *J* = 8.8 Hz, 2H), 7.18 (d, *J* = 8.4 Hz, 1H), 7.25 – 7.28 (m, 2H, overlapped), 7.48 (d, *J* = 8.4 Hz, 2H) ppm. ^{13}C NMR (100 MHz, $CDCl_3$) δ = 20.3, 21.7, 38.4, 55.5, 113.6, 125.7, 127.8, 128.2, 129.4, 130.3, 130.9, 133.5, 133.8, 135.0, 139.4, 142.1, 143.6, 158.8 ppm. HRMS (ESI$^+$) calcd for $C_{22}H_{23}NNaO_3S$ [M+Na]$^+$ 404.1291, found 404.1292. IR (KBr): 2960, 2923, 2848, 1655, 1631, 1469, 1262, 1022, 807, 721, 669 cm^{-1}.

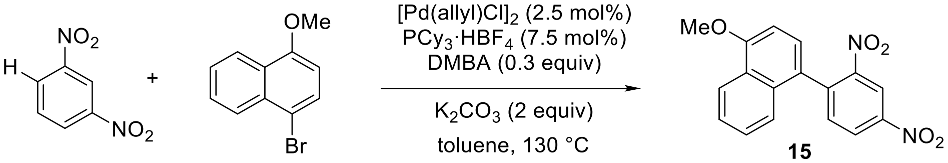

15

1-(2,4-Dinitrophenyl)-4-methoxynaphthalene (15): Compound **15** was synthesized by a modified procedure of literature.[27] An oven-dried vial equipped with a stirring bar was charged with 1,3-dinitrobenzene (504 mg, 4 mmol, 2.0 equiv), 1-bromo-4-methoxynaphthalene (474 mg, 2 mmol, 1.0 equiv), $[Pd(allyl)Cl]_2$ (19 mg, 2.5 mol%), $PCy_3·HBF_4$ (55 mg, 7.5 mol%), 2,2-dimethylbutanoic acid (75 µL, 0.3 equiv) and K_2CO_3 (550 mg, 2.0 equiv) under N_2. Toluene (5 mL) was added at room temperature. The reaction mixture was placed in a preheated oil bath at 130 °C, and stirred for 24 h. The reaction mixture was then cooled down to room temperature, diluted with CH_2Cl_2 (50 mL), filtered through celite, and concentrated. Purification by chromatography on silica gel (200-300 mesh, petroleum ether/ CH_2Cl_2 = 2/1) afforded the corresponding product **15** as a red solid (337 mg, 52% yield). ^{1}H NMR (400 MHz, $CDCl_3$): *δ* = 4.06 (s, 3H), 6.88 (d, *J* = 8.0 Hz, 1H), 7.27 (d, *J* = 8.0 Hz, 1H), 7.35 (d, *J* = 8.0 Hz, 1H), 7.44 – 7.49 (m, 1H), 7.50 – 7.55 (m, 1H), 7.73 (d, *J* = 8.4 Hz, 1H), 8.37 (d, *J* = 9.2 Hz, 1H), 8.52 (dd, *J* = 8.4, 2.4 Hz, 1H), 8.86 (d, *J* = 2.4 Hz, 1H) ppm. ^{13}C NMR (100 MHz, $CDCl_3$): *δ* = 55.8, 103.4, 119.9, 123.0, 124.0, 125.3, 125.8, 126.0, 126.6, 126.8, 127.8, 131.8, 135.0, 141.9, 147.2, 150.3, 156.8. ppm. HRMS (ESI$^+$) calcd for $C_{17}H_{13}N_2O_5$ [M+H]$^+$ 325.0819, found 325.0818. IR (KBr): 3111, 2945, 2841, 1590, 1515, 1341, 1253, 1106, 1084, 815, 772, 740 cm^{-1}.

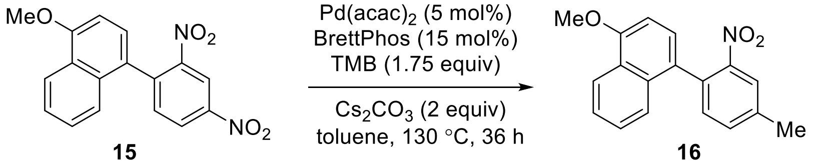

15 16

1-Methoxy-4-(4-methyl-2-nitrophenyl)naphthalene (16): Compound **16** was synthesized according to the general methylation procedure of nitro group from **15**. The reaction was conducted at 130 °C for 36 h. The corresponding product **15** was obtained as a yellow solid (26 mg, 44% yield). ^{1}H NMR (400 MHz, $CDCl_3$) δ = 2.53 (s, 3H), 4.04 (s, 3H), 6.84 (d, *J* = 8.0 Hz, 1H), 7.23

(d, J = 8.0 Hz, 1H), 7.35 (d, J = 8.0 Hz, 1H), 7.39 – 7.44 (m, 2H), 7.44 – 7.51 (m, 2H), 7.83 (s, 1H), 8.33 (d, J = 8.4 Hz, 1H) ppm. ^{13}C NMR (100 MHz, $CDCl_3$) δ = 21.1, 55.7, 103.4, 122.6, 124.6, 124.8, 125.4, 125.7, 126.4, 127.1, 127.7, 132.5, 132.7, 133.3, 133.4, 139.0, 150.2, 155.7 ppm. HRMS (ESI^+) calcd for $C_{18}H_{15}NNaO_3$ $[M+Na]^+$ 316.0944, found 316.0947. IR (KBr): 2924, 2850, 1580, 1517, 1464, 1341, 1254, 1087, 817, 767 cm^{-1}.

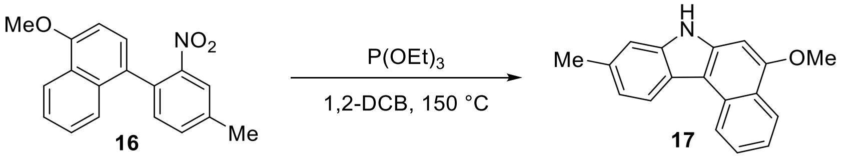

5-Methoxy-9-methyl-7*H*-benzo[*c*]carbazole (17): Compound **17** was synthesized by Cadogen-type reaction. An oven-dried vial equipped with a stirring bar was charged with nitroaromatic **16** (15 mg, 0.05 mmol, 1.0 equiv) under N_2. A mixture of $P(OEt)_3$ and 1,2-dichlorobenzene (v/v = 1/1, 0.3 mL) was added at room temperature. The reaction mixture was placed in a preheated oil bath at 150 °C, and stirred for 12 h. The reaction mixture was then cooled down to room temperature, diluted with CH_2Cl_2 (10 mL), filtered through celite, and concentrated. Purification by chromatography on silica gel (200-300 mesh, petroleum ether/ CH_2Cl_2 = 2/1) afforded the corresponding product **17** as a white solid (13 mg, 98% yield). ^1H NMR (400 MHz, $CDCl_3$) δ = 2.55 (s, 3H), 4.03 (s, 3H), 6.83 (s, 1H), 7.18 (d, J = 8.1 Hz, 1H), 7.23 (s, 1H), 7.43 – 7.50 (m, 1H), 7.66 – 7.74 (m, 1H), 8.05 (br s, 1H), 8.31 (d, J = 8.1 Hz, 1H), 8.39 (d, J = 8.3 Hz, 1H), 8.66 (d, J = 8.3 Hz, 1H) ppm. ^{13}C NMR (100 MHz, $CDCl_3$) δ = 22.0, 55.9, 91.6, 109.4, 111.1, 120.7, 121.8, 122.2, 122.3, 122.5, 123.2, 123.4, 127.5, 130.3, 133.0, 137.6, 138.8, 155.2 ppm. HRMS (ESI^+) calcd for $C_{18}H_{15}NO$ $[M]^+$ 261.1148, found 261.1156. IR (KBr): 3360, 2963, 2921, 2853, 2142, 1586, 1512, 1462, 1389, 1260, 1027, 968, 797, 766 cm^{-1}.

IX. Mechanistic studies

8.1 General procedure for competition experiments

An oven-dried vial equipped with a stirring bar was charged with two substrates (0.2 mmol, 1.0 equiv), TMB (100 μL, 3.5*N* in THF, 3.5 mmol, 1.75 equiv), catalyst, ligand and base (0.4 mmol, 2.0 equiv), placed under a positive pressure of N_2. Solvent (0.6 mL) was added at room temperature. The reaction mixture was placed in a preheated oil bath at 160 °C, and stirred for 4h. The reaction mixture was then cooled down to room temperature. Diphenylacetylene (35.6 mg, 0.2 mmol) and mesitylene (27.8 μL, 0.2 mmol) were subjected to the reaction mixture as the internal standard. The yields of **2f** and **2h** were separately

determined by GC analysis using diphenylacetylene and mesitylene as the internal standard, respectively.

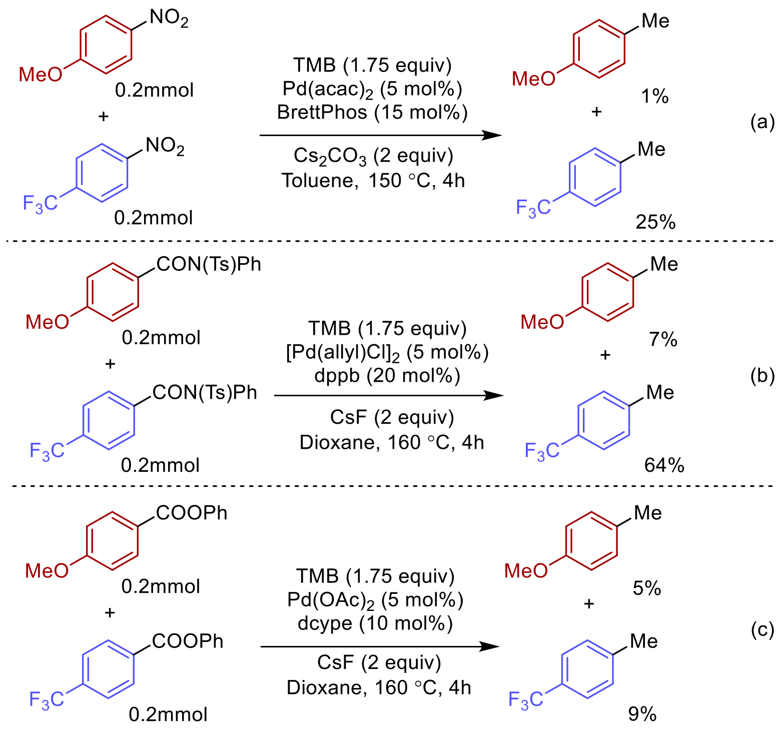

Scheme S4 Competition experiments.

8.2 Competition experiment for denitrative methylation

The yield of **2f** was determined by GC analysis using calibration curves based on data from the authentic sample of **2f** and diphenylacetylene (1% yield).

fby-3936-1 D:\data\zs\fby-3936-1.qgd

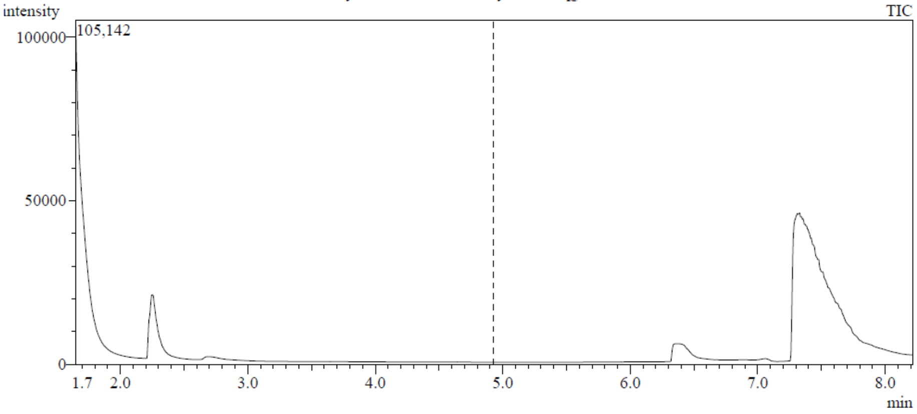

	Compound	RTime	m/z	Area	Height	Conc.
1	p-methylanisole	2.689	122.00	5934	760	0.013
2	diphenylacetylene	7.312	177.90	633594	33388	1.000

Calibration Curve

ID#:1 m/z:122.00 Name:p-methylanisole
f(x)=0.709868*x+0.000000
rr1=0.999478 rr2=0.998957
MeanRF:0.68 RFSD:0.06 RFRSD:8.97
CurveType:Least square
ZeroThrough:Yes
WeightedRegression:No
Internal Standard

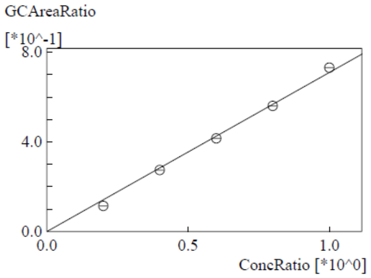

#	ConcRatio	GCAreaRatio	GCArea of IntStandard
1	1.000	0.73	384885.00
2	0.800	0.56	280771.00
3	0.600	0.42	196416.00
4	0.400	0.27	113751.00
5	0.200	0.11	47479.00

The yield of **2h** was determined by GC analysis using calibration curves based on data from the authentic sample of **2h** and mesitylene (25% yield).

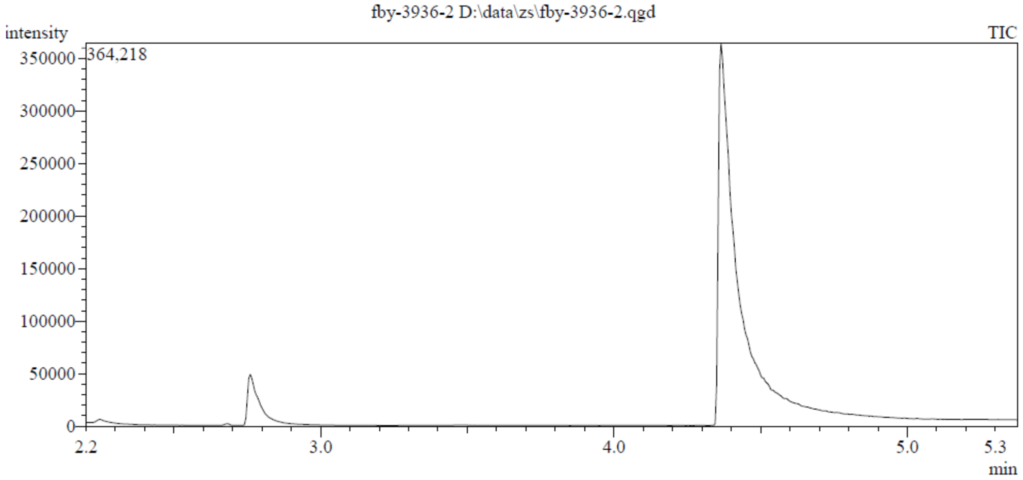

	Compound	RTime	m/z	Area	Height	Conc.
1	4-(trifluoromethyl)-toluene	2.761	159.90	78365	29245	0.252
2	Mes	4.367	120.00	441349	107588	1.000

Calibration Curve

ID#:1 m/z:159.90 Name:4-(trifluoromethyl)-toluene
f(x)=0.705366*x+0.000000
rr1=0.986979 rr2=0.974128
MeanRF:0.68 RFSD:0.07 RFRSD:9.64
CurveType:Least square
ZeroThrough:Yes
WeightedRegression:No
Internal Standard

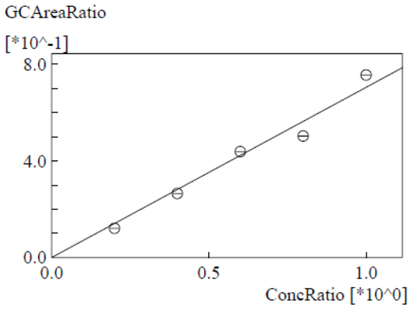

#	ConcRatio	GCAreaRatio	GCArea of IntStandard
1	1.000	0.76	888122.00
2	0.800	0.50	636673.00
3	0.600	0.44	467292.00
4	0.400	0.27	306275.00
5	0.200	0.12	123823.00

8.3 Competition experiment for decarbonylative methylation of amide

The yield of **2f** was determined by GC analysis using calibration curves based on data from the authentic sample of **2f** and diphenylacetylene (7% yield).

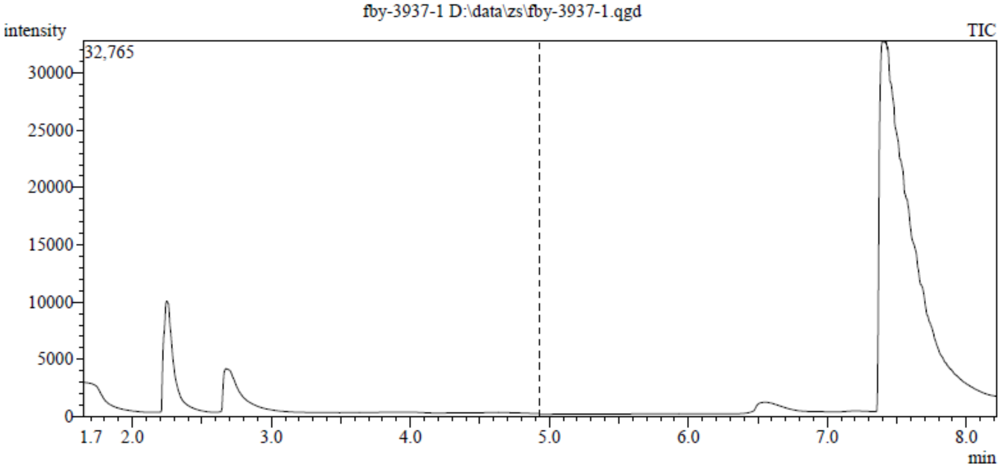

	Compound	RTime	m/z	Area	Height	Conc.
1	p-methylanisole	2.677	122.00	17771	2199	0.074
2	diphenylacetylene	7.402	177.90	339008	24123	1.000

Calibration Curve

ID#:1 m/z:122.00 Name:p-methylanisole
f(x)=0.709868*x+0.000000
rr1=0.999478 rr2=0.998957
MeanRF:0.68 RFSD:0.06 RFRSD:8.97
CurveType:Least square
ZeroThrough:Yes
WeightedRegression:No
Internal Standard

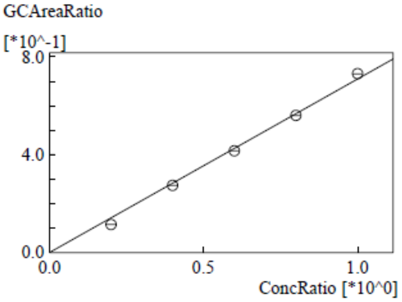

#	ConcRatio	GCAreaRatio	GCArea of IntStandard
1	1.000	0.73	384885.00
2	0.800	0.56	280771.00
3	0.600	0.42	196416.00
4	0.400	0.27	113751.00
5	0.200	0.11	47479.00

The yield of **2h** was determined by GC analysis using calibration curves based on data from the authentic sample of **2h** and mesitylene (64% yield).

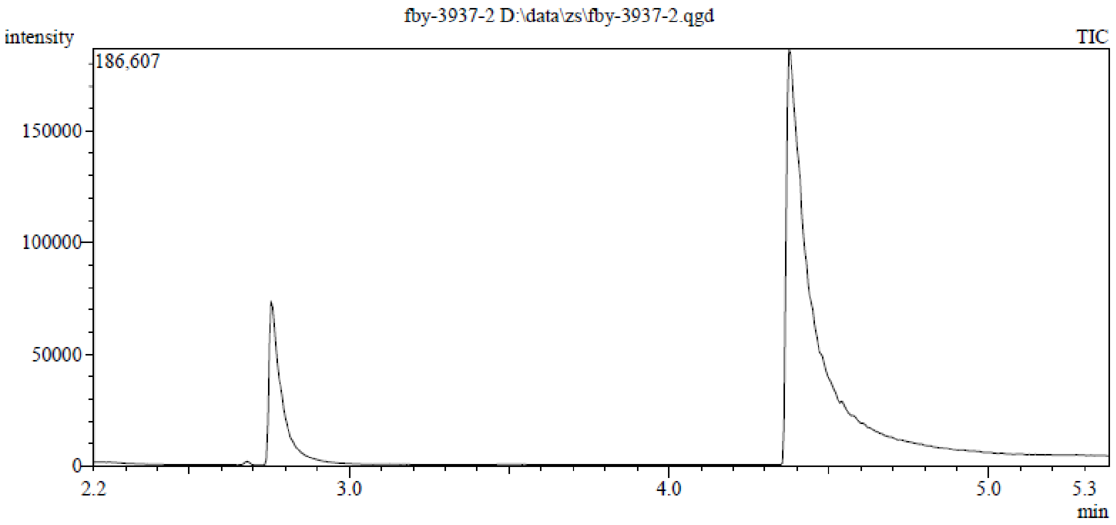

	Compound	RTime	m/z	Area	Height	Conc.
1	4-(trifluoromethyl)-toluene	2.757	159.90	116054	43469	0.640
2	Mes	4.379	120.00	256887	54969	1.000

Calibration Curve

ID#:1 m/z:159.90 Name:4-(trifluoromethyl)-toluene
f(x)=0.705366*x+0.000000
rr1=0.986979 rr2=0.974128
MeanRF:0.68 RFSD:0.07 RFRSD:9.64
CurveType:Least square
ZeroThrough:Yes
WeightedRegression:No
Internal Standard

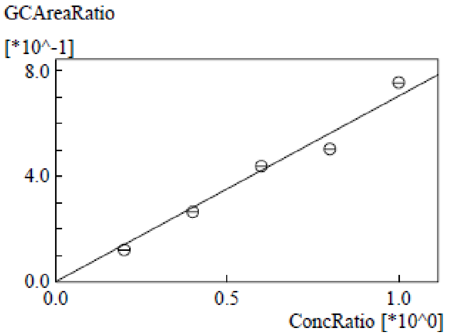

#	ConcRatio	GCAreaRatio	GCArea of IntStandard
1	1.000	0.76	888122.00
2	0.800	0.50	636673.00
3	0.600	0.44	467292.00
4	0.400	0.27	306275.00
5	0.200	0.12	123823.00

8.4 Competition experiment for decarbonylative methylation of ester

The yield of **2f** was determined by GC analysis using calibration curves based on data from the authentic sample of **2f** and diphenylacetylene (5% yield).

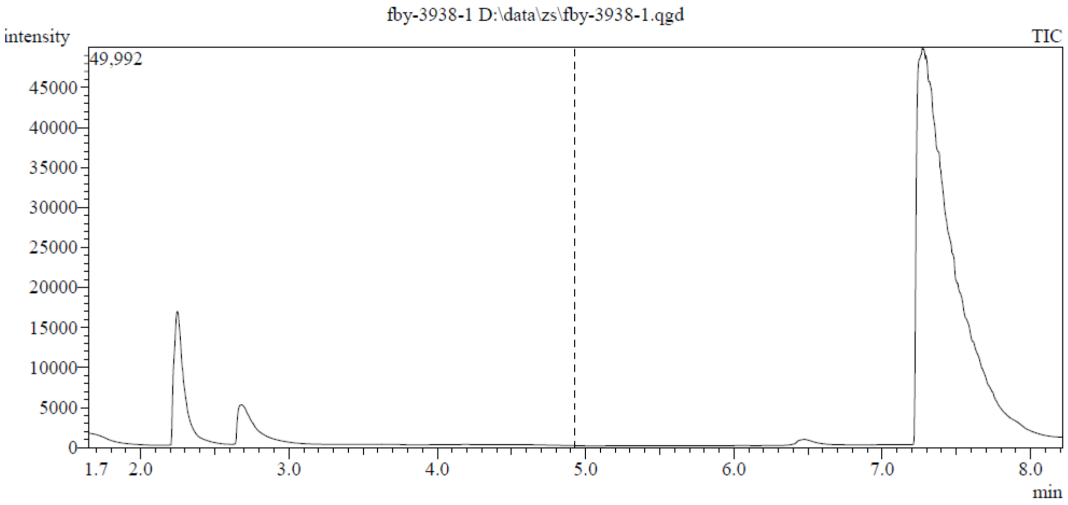

	Compound	RTime	m/z	Area	Height	Conc.
1	p-methylanisole	2.683	122.00	21925	2860	0.053
2	diphenylacetylene	7.274	177.90	581143	36994	1.000

Calibration Curve

ID#:1 m/z:122.00 Name:p-methylanisole
f(x)=0.709868*x+0.000000
rr1=0.999478 rr2=0.998957
MeanRF:0.68 RFSD:0.06 RFRSD:8.97
CurveType:Least square
ZeroThrough:Yes
WeightedRegression:No
Internal Standard

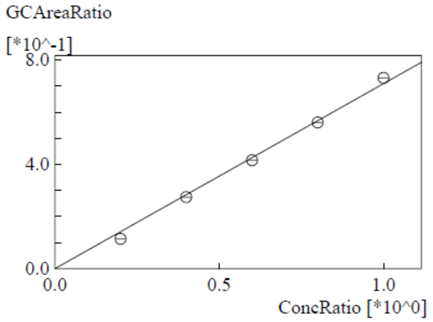

#	ConcRatio	GCAreaRatio	GCArea of IntStandard
1	1.000	0.73	384885.00
2	0.800	0.56	280771.00
3	0.600	0.42	196416.00
4	0.400	0.27	113751.00
5	0.200	0.11	47479.00

The yield of **2h** was determined by GC analysis using calibration curves based on data from the authentic sample of **2h** and mesitylene (9% yield).

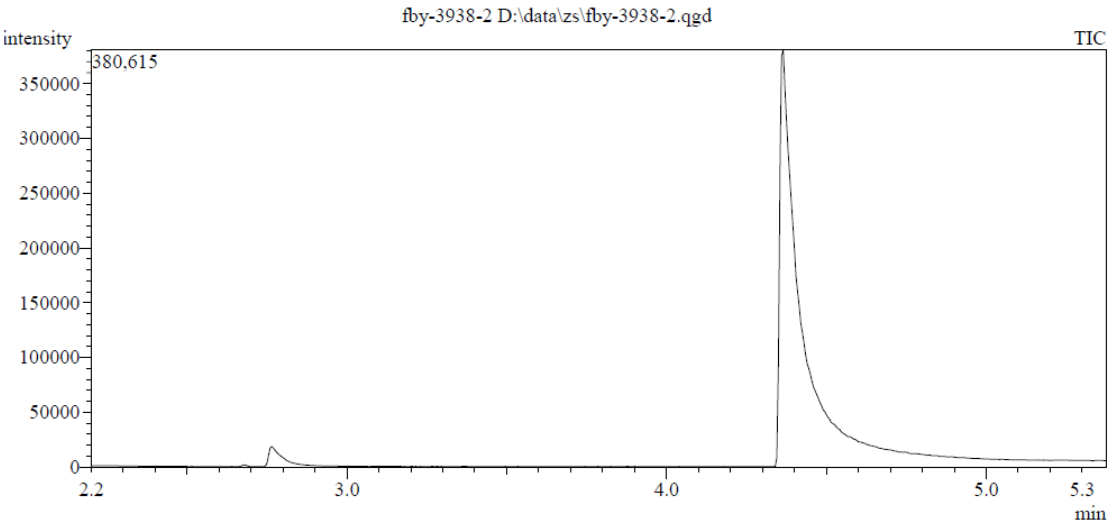

	Compound	RTime	m/z	Area	Height	Conc.
1	4-(trifluoromethyl)-toluene	2.765	159.90	27922	10916	0.088
2	Mes	4.365	120.00	449448	116130	1.000

Calibration Curve

ID#:1 m/z:159.90 Name:4-(trifluoromethyl)-toluene
f(x)=0.705366*x+0.000000
rr1=0.986979 rr2=0.974128
MeanRF:0.68 RFSD:0.07 RFRSD:9.64
CurveType:Least square
ZeroThrough:Yes
WeightedRegression:No
Internal Standard

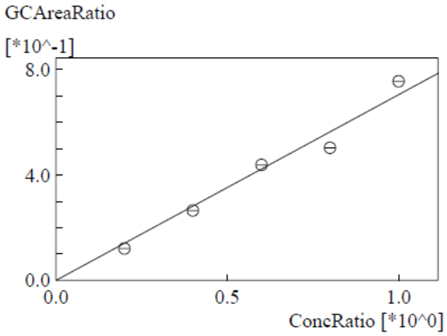

#	ConcRatio	GCAreaRatio	GCArea of IntStandard
1	1.000	0.76	888122.00
2	0.800	0.50	636673.00
3	0.600	0.44	467292.00
4	0.400	0.27	306275.00
5	0.200	0.12	123823.00

X. References

(1) G. Kunstle and H. Siegl, US Pat., 3960909A, 1976.

(2) B. Feng, Y. Yang and J. You, *Chem. Commun.*, 2020, **56**, 790.

(3) S.-L. Zhang, Z. Yang, X. Hu and K. Y. Tam, *Bioorg. Med. Chem. Lett.*, 2018, **28**, 3441.

(4) O. Phuangsawai, P. Beswick, S. Ratanabunyong, L. Tabtimmai, P. Suphakun, P. Obounchoey, P. Srisook, N. Horata, I. Chuckowree, S. Hannongbua, S. E. Ward, Kiattawee Choowongkomon and M. P. Gleeson, *Eur. J. Med. Chem.*, 2016, **124**, 896.

(5) M. J. S. Dewar and E. W. T. Warford, *J. Chem. Soc.*, 1956, 3570.

(6) J. Frommer, B. Karg, K. Weisz and Sabine Müller, *Org. Biomol. Chem.*, 2018, *16*, 7663.

(7) Y.-J. Pu, M. Higashidate, K.-i. Nakayama and J. Kido, *J. Mater. Chem.*, 2008, **18**, 4183.

(8) A. J. Tomson and J. P. Horwitz, *J. Org. Chem.*, 1959, **24**, 2056.

(9) H. Wu, W. Guo, S. Daniel, Y. Li, C. Liu and Z. Zeng, *Chem. –Eur. J.*, 2018, **24**, 3444.

(10) X. Li and G. Zou, *Chem. Commun.*, 2015, **51**, 5089.

(11) J. Chen, Y. Peng, M. Liu, J. Ding, W. Su and H. Wua, *Adv. Synth. Catal.*, 2012, **354**, 2117.

(12) X. Hua, J. Masson-Makdissi, R. J. Sullivan and S. G. Newman, *Org. Lett.*, 2016, **18**, 5312.

(13) L. Guo and M. Rueping, *Chem. –Eur. J.*, 2016, **22**, 16787.

(14) T. Ueda, H. Konishi and K. Manabe, *Org. Lett.*, 2012, **14**, 3100.

(15) C. Qin, H. Wu, J. Chen, M. Liu, J. Cheng, W. Su and J. Ding, *Org. Lett.*, 2008, **10**, 1537.

(16) F. Zeng and H. Alper, *Org. Lett.*, 2011, **13**, 2868.

(17) J. Masson-Makdissi, J. K. Vandavasi and S. G. Newman, *Org. Lett.*, 2018, **20**, 4094.

(18) T. Morioka, A. Nishizawa, K. Nakamura, M. Tobisu and N. Chatani, *Chem. Lett.*, 2015, **44**, 1729.

(19) Q. Yao, W. Liu, P. Liu, L. Ren, X. Fang and C.-J. Li, *Eur. J. Org. Chem.*, 2019, 2721.

(20) S. Zhou, Z. Yang, X. Chen, Y. Li, L. Zhang, H. Fang, W. Wang, X. Zhu and S. Wang, *J. Org. Chem.*, 2015, **80**, 6323.

(21) D. Zhu and L. Shi, *Chem. Commun.*, 2018, **54**, 9313–9316.

(22) Q. Wu, S. Han, X. Ren, H. Lu, J. Li, D. Zou, Y. Wu and Y. Wu, *Org. Lett.*, 2018, **20**, 6345.

(23) Y. Zhu, H. Yan, L. Lu, D. Liu, G. Rong and J. Mao, *J. Org. Chem.*, 2013, **78**, 9898.

(24) A. M. Haydl and J. F. Hartwig, *Org. Lett.*, 2019, **21**, 1337.

(25) P. F. Cirillo, A. Hammach, V. Kamhi, N. Moss, P. S. Riska and C. Pargellis, WO Pat. 2004014870A1, 2004.

(26) L. Martínez-García, R. Lobato, G. Prado, P. Monje, F. J. Sardina and M. R. Paleo, *J. Org. Chem.,* 2019, **84**, 1887.

(27) L. Caron, L.-C. Campeau and K. Fagnou, *Org. Lett.*, 2008, **10**, 4533.

XI. Copies of NMR spectra

^{1}H NMR spectra of **1o**:

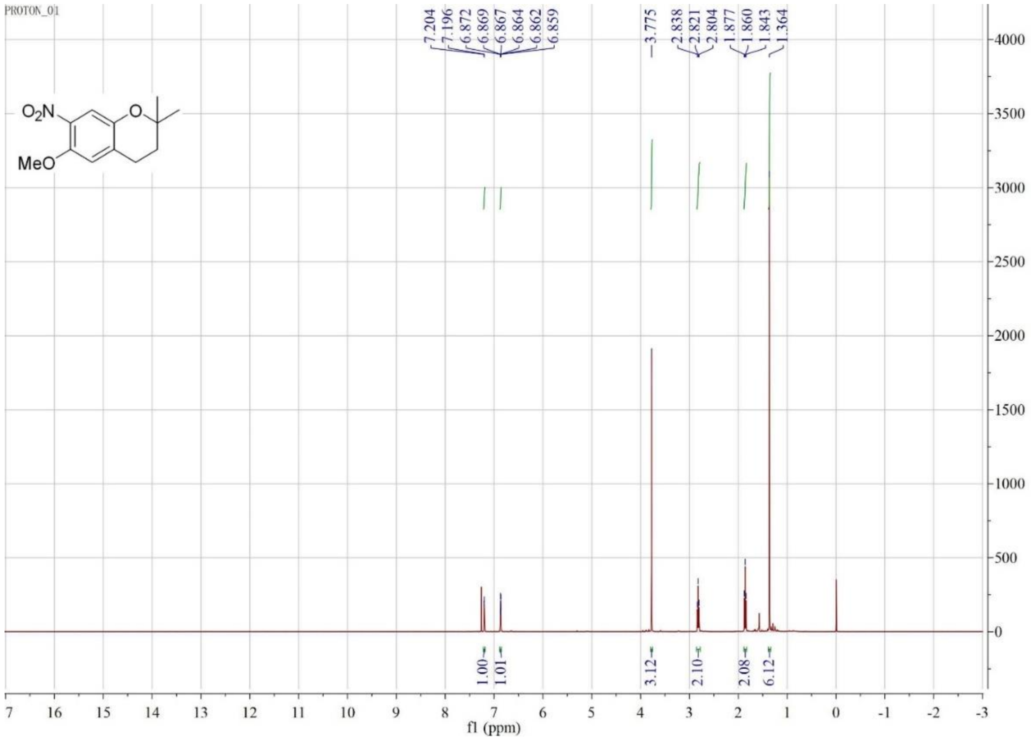

^{13}C NMR spectra of **1o**:

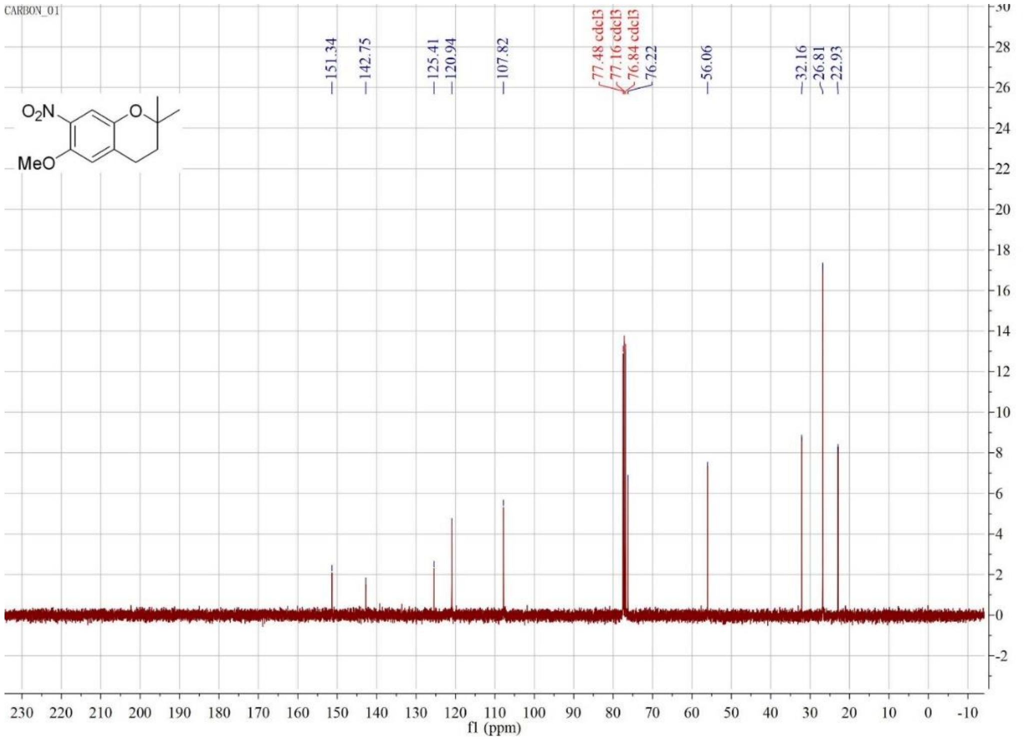

^{1}H NMR spectra of **2a**:

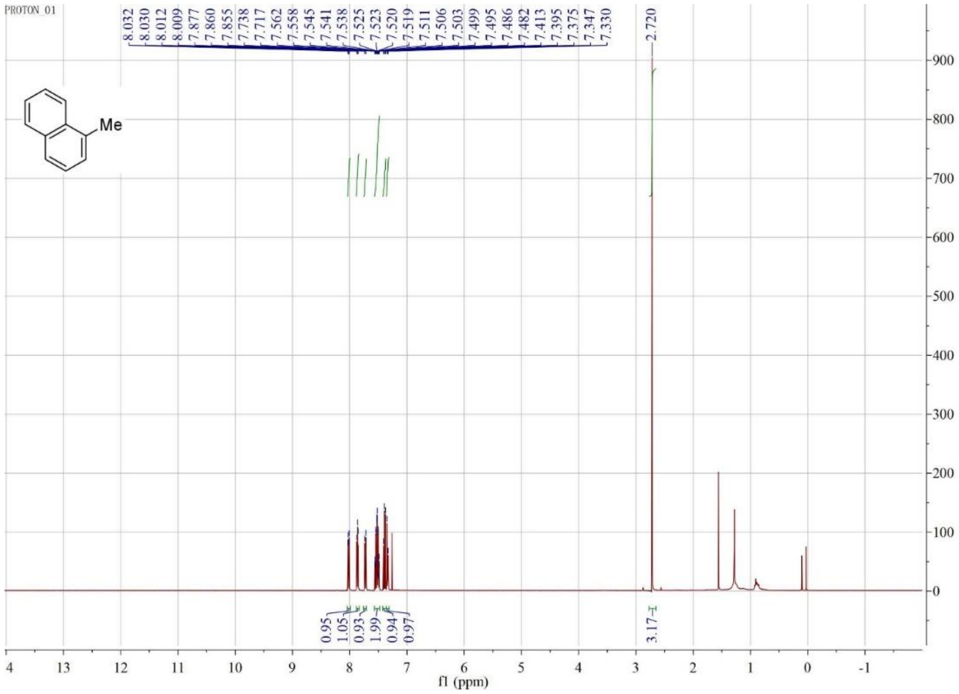

^{13}C NMR spectra of **2a**:

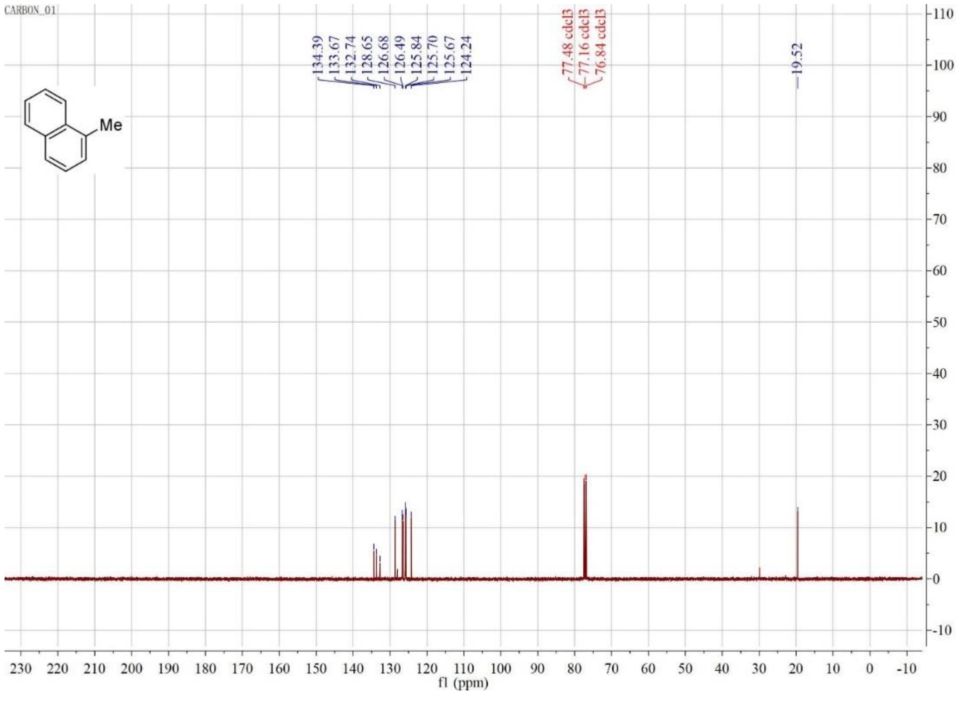

^{1}H NMR spectra of **2b**:

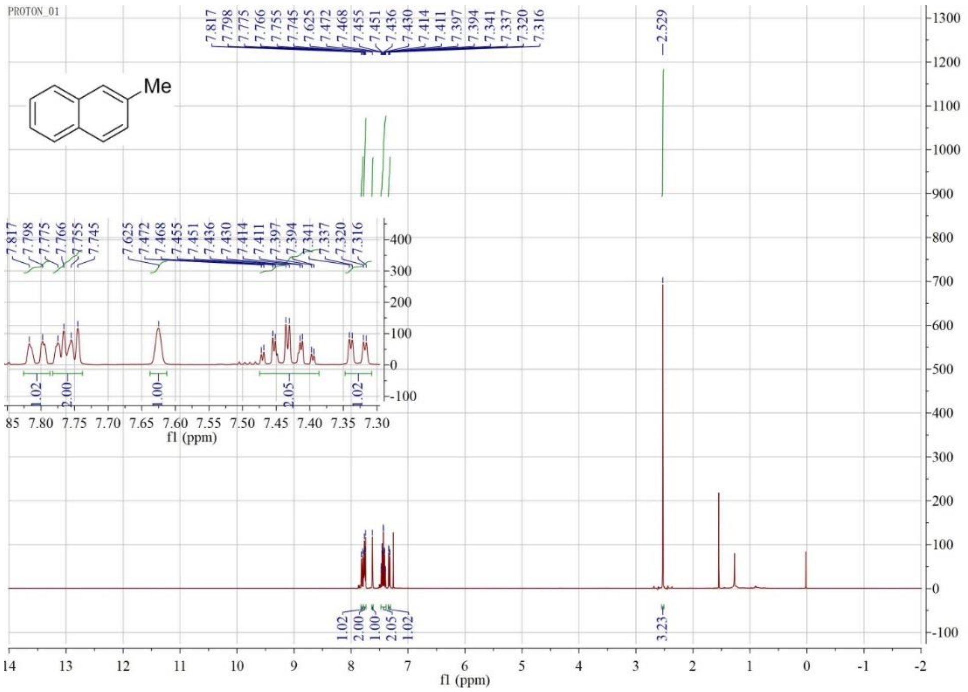

^{13}C NMR spectra of **2b**:

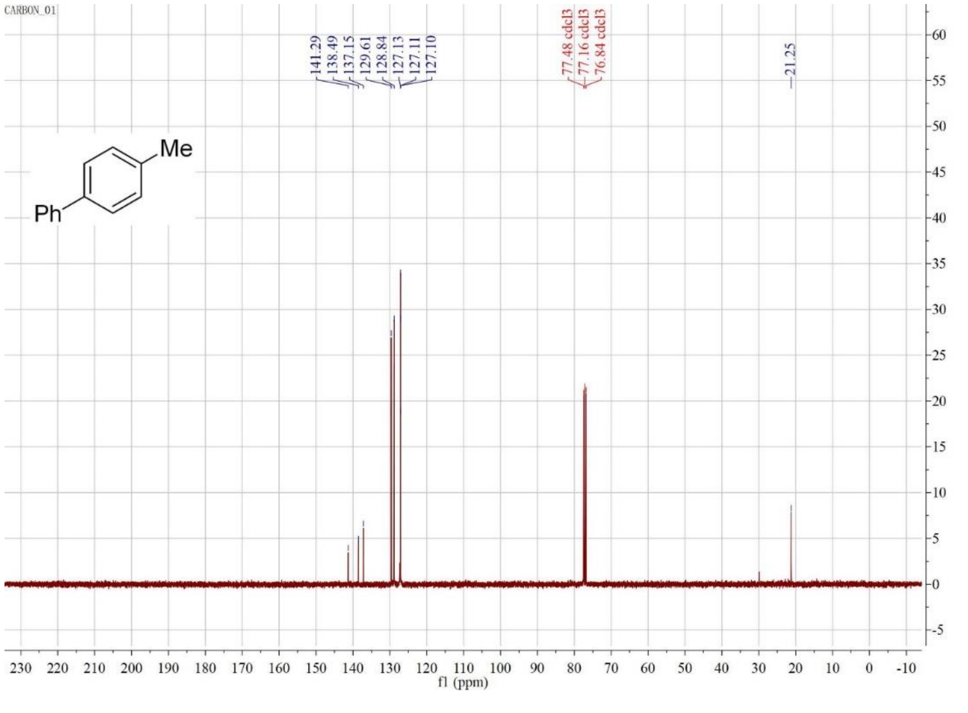

^{1}H NMR spectra of **2c**:

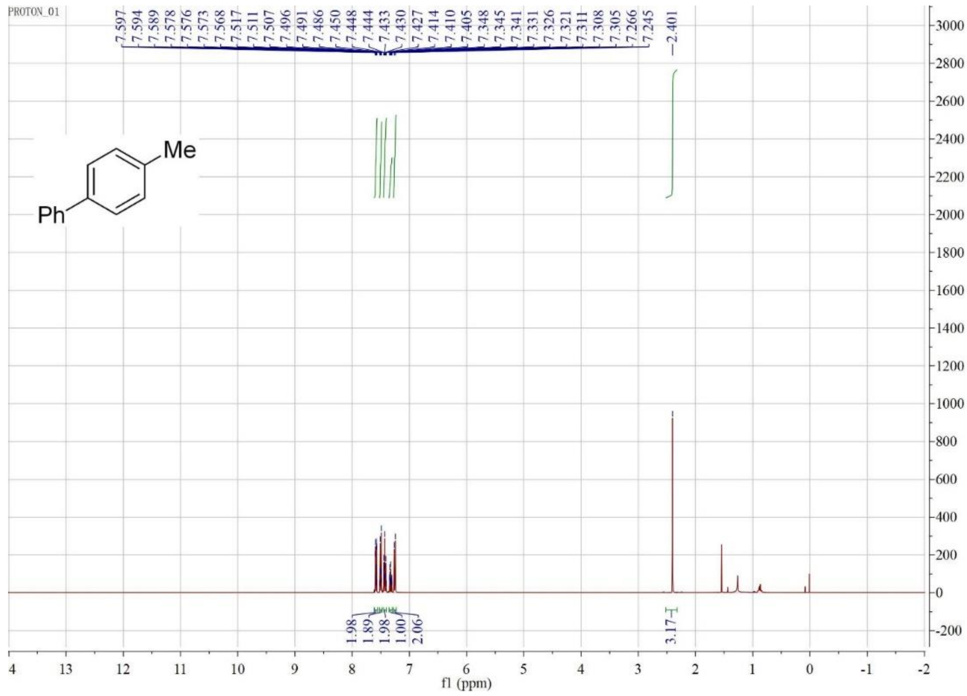

^{13}C NMR spectra of **2c**:

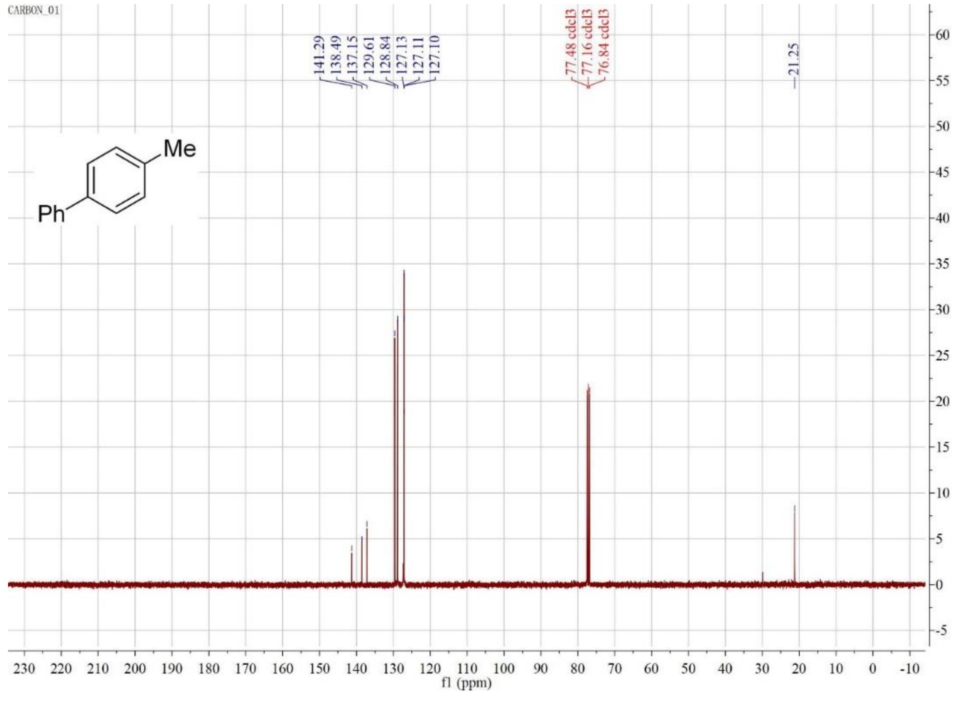

^{1}H NMR spectra of **2d**:

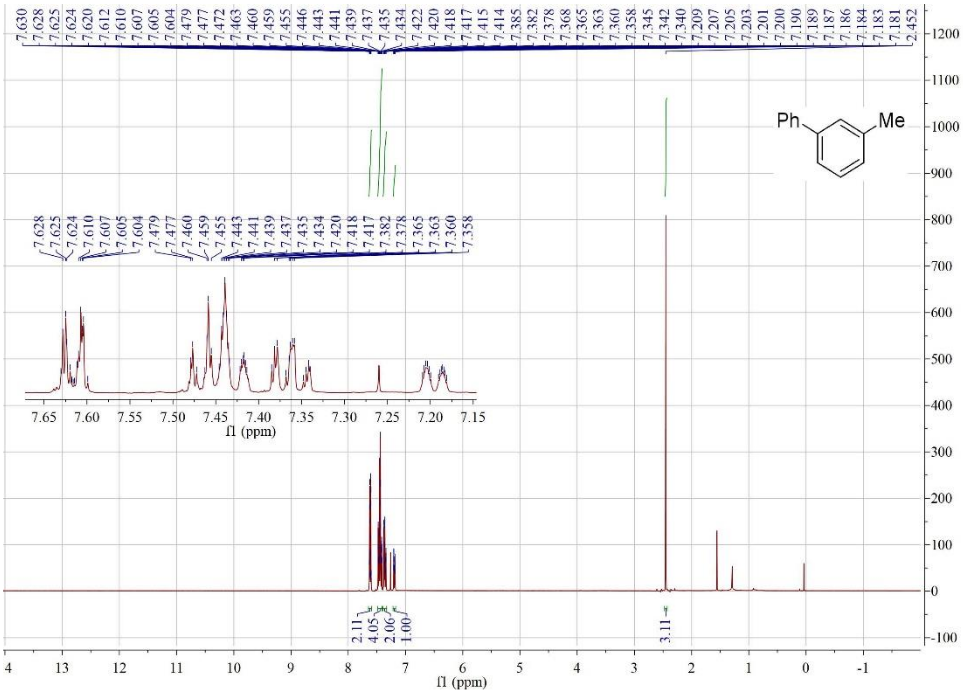

^{13}C NMR spectra of **2d**:

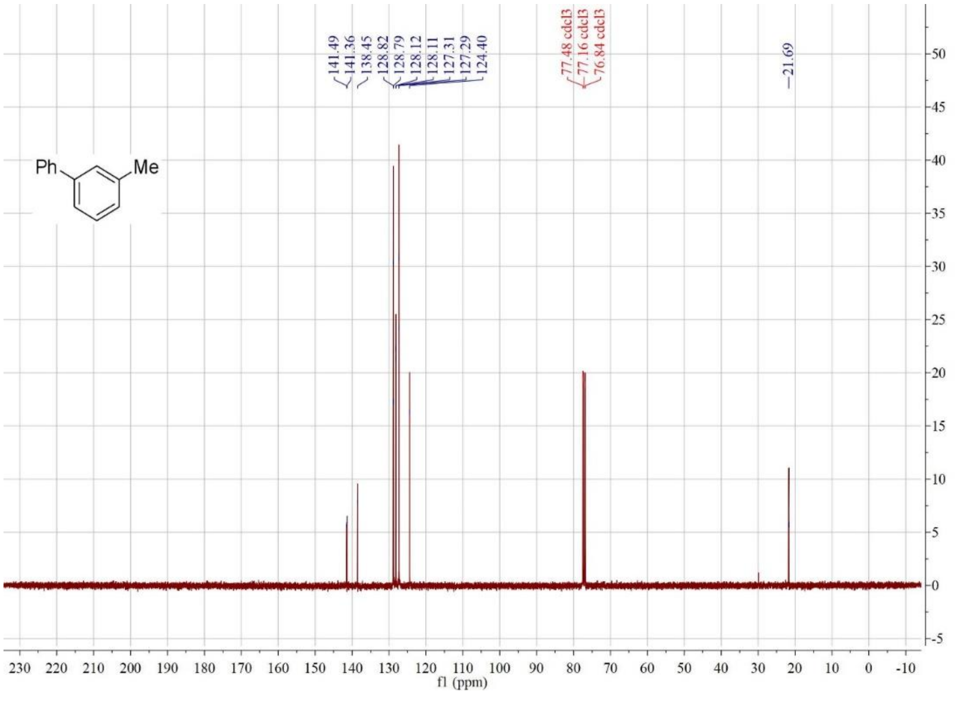

^{1}H NMR spectra of **2e**:

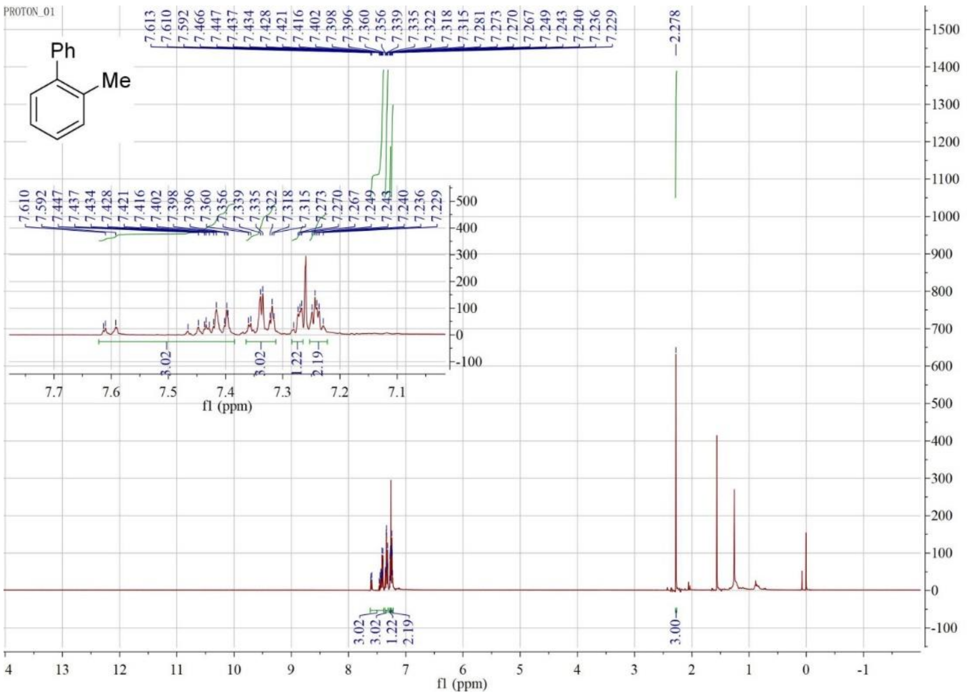

^{13}C NMR spectra of **2e**:

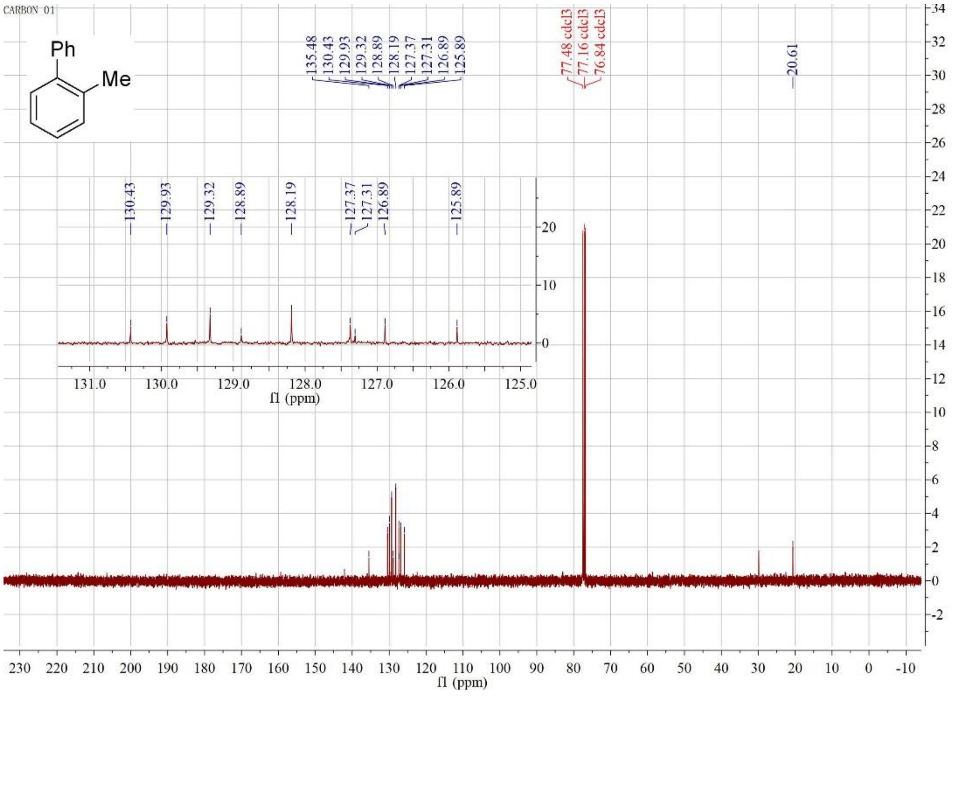

^{1}H NMR spectra of **2i**:

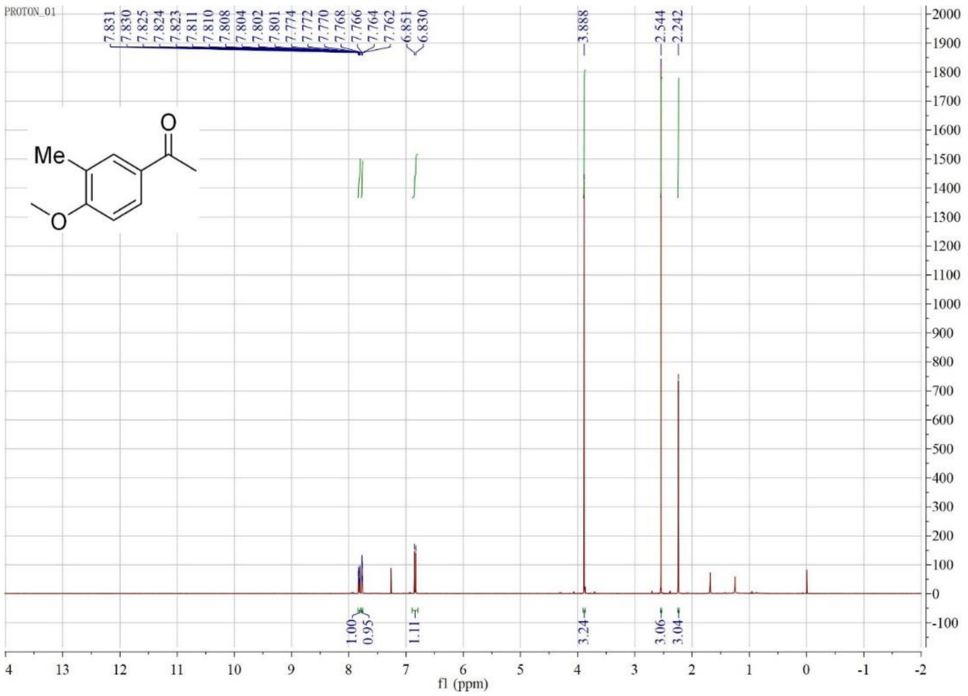

^{13}C NMR spectra of **2i**:

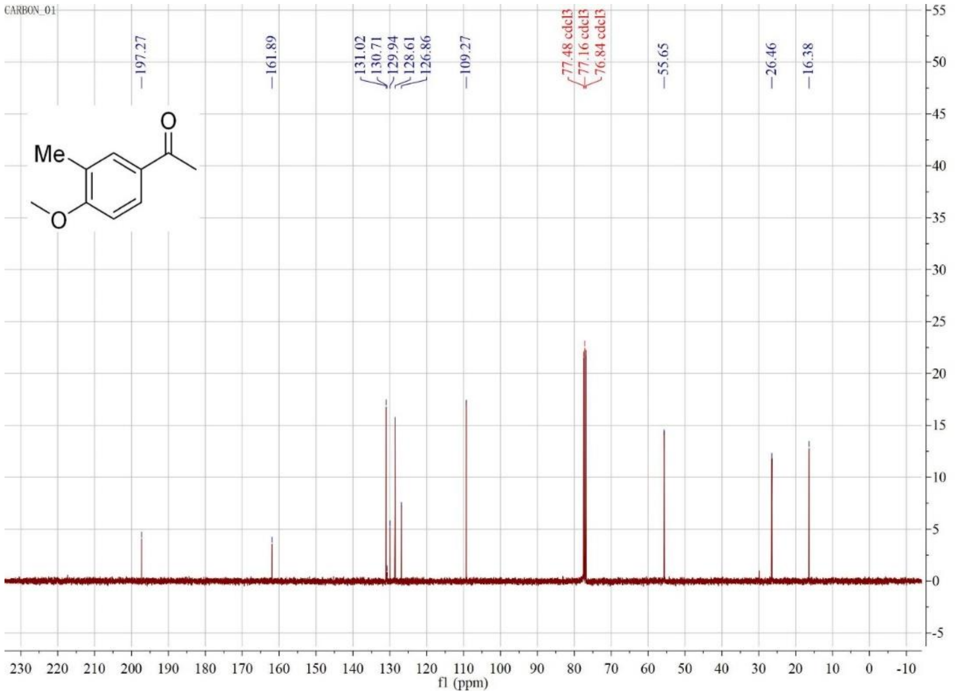

^{1}H NMR spectra of **2j**:

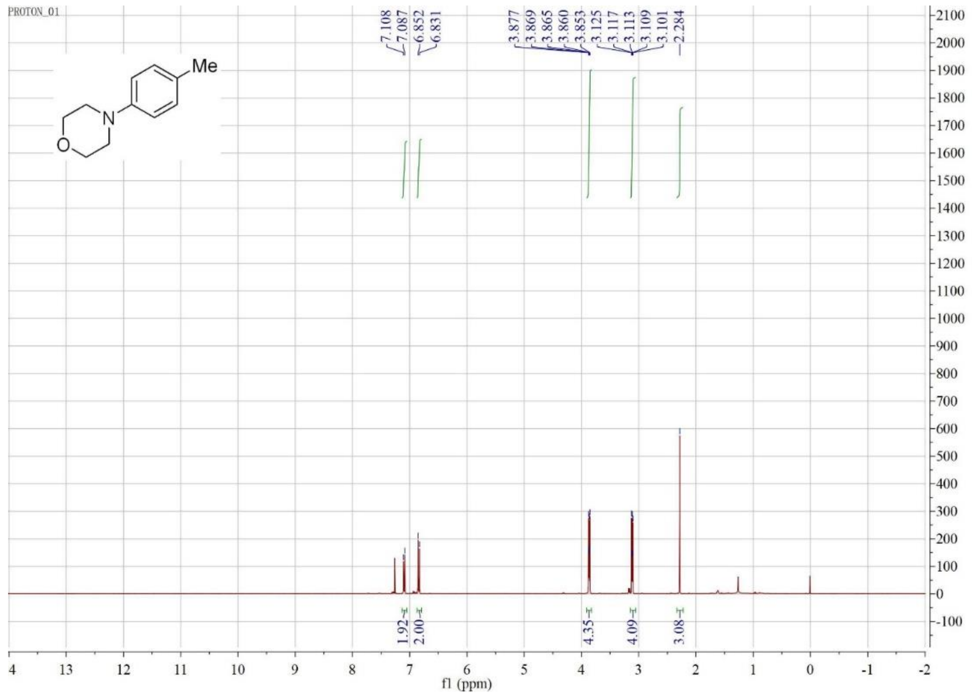

^{13}C NMR spectra of **2j**:

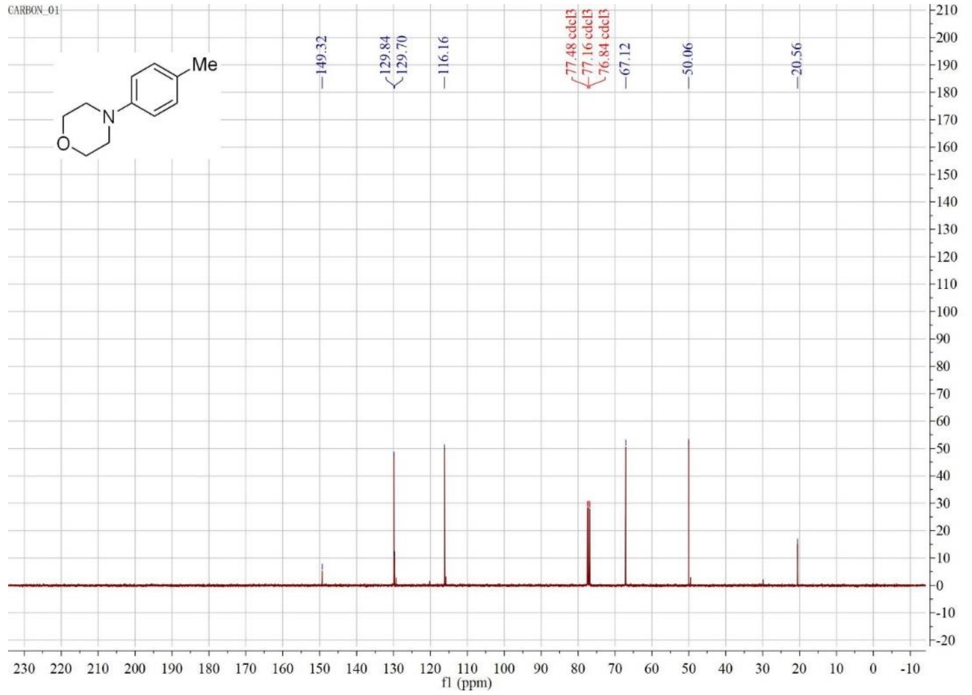

^{1}H NMR spectra of **2k**:

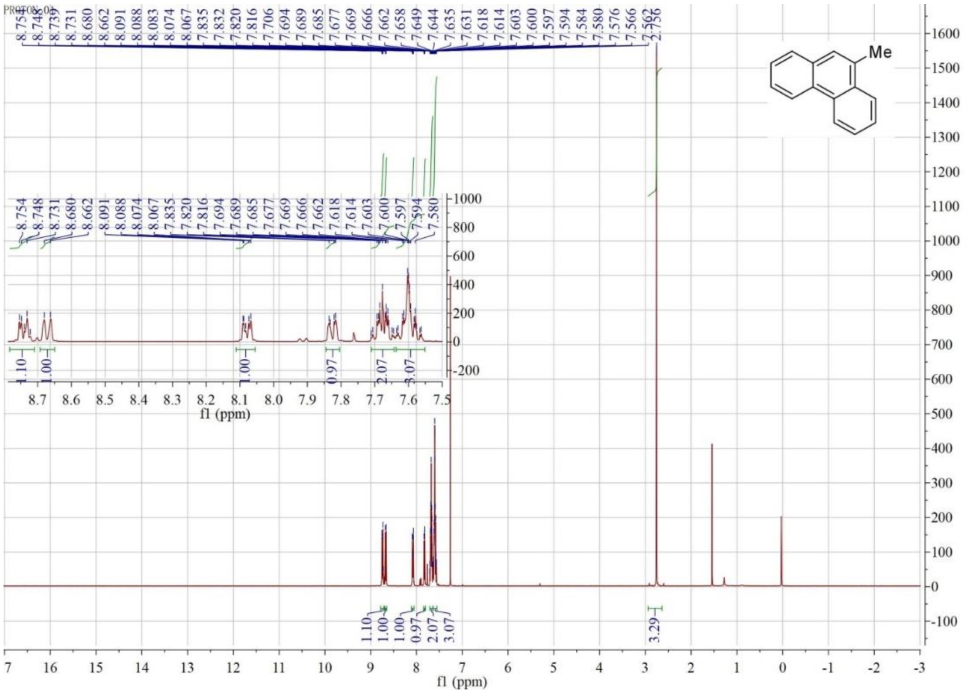

^{13}C NMR spectra of **2k**:

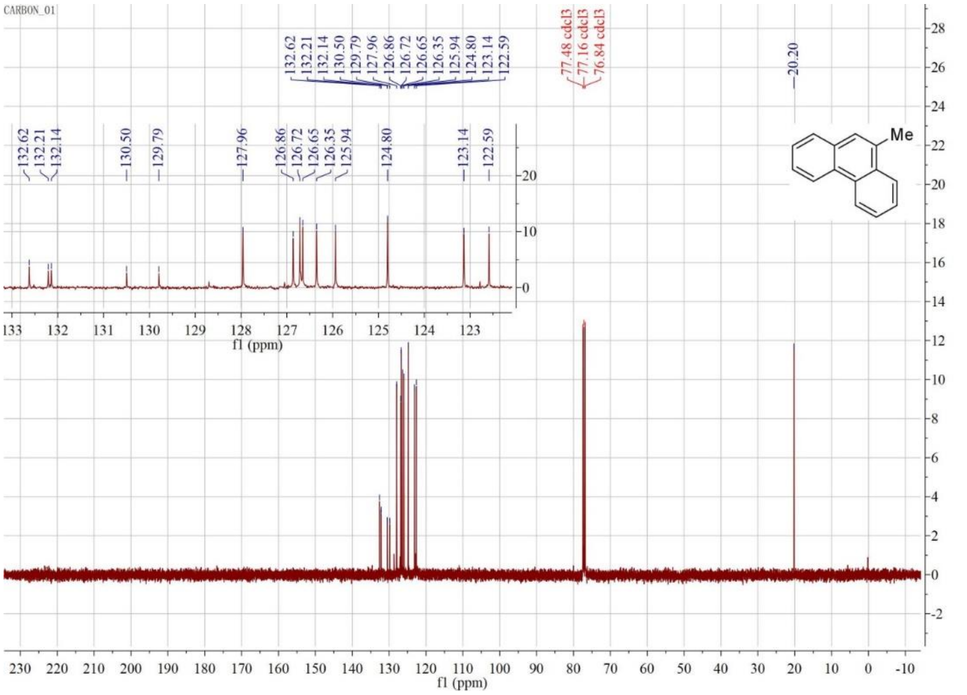

^{1}H NMR spectra of **2l**:

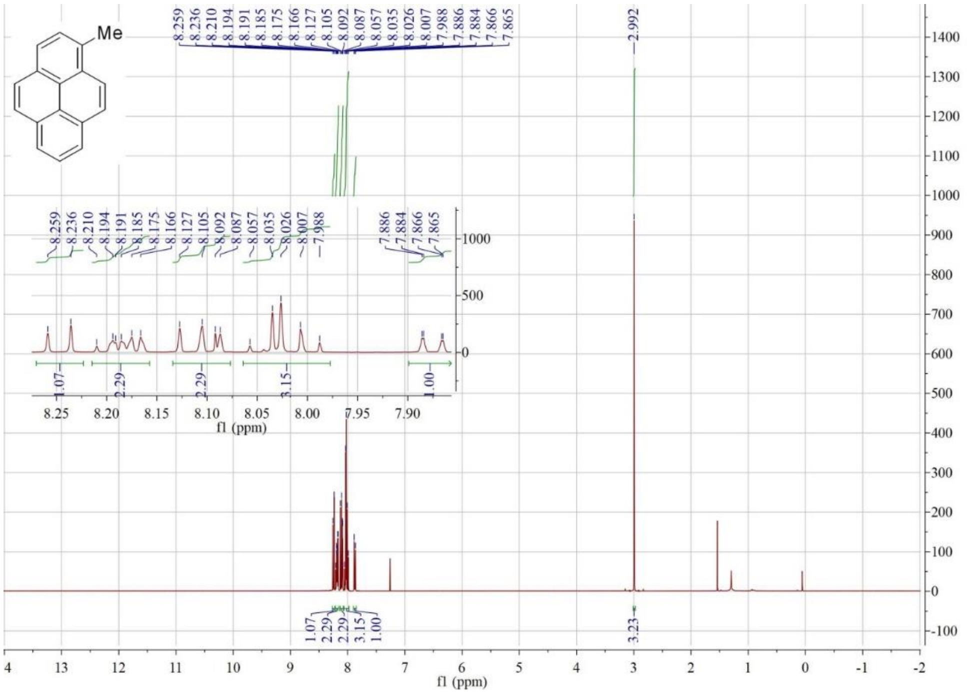

^{13}C NMR spectra of **2l**:

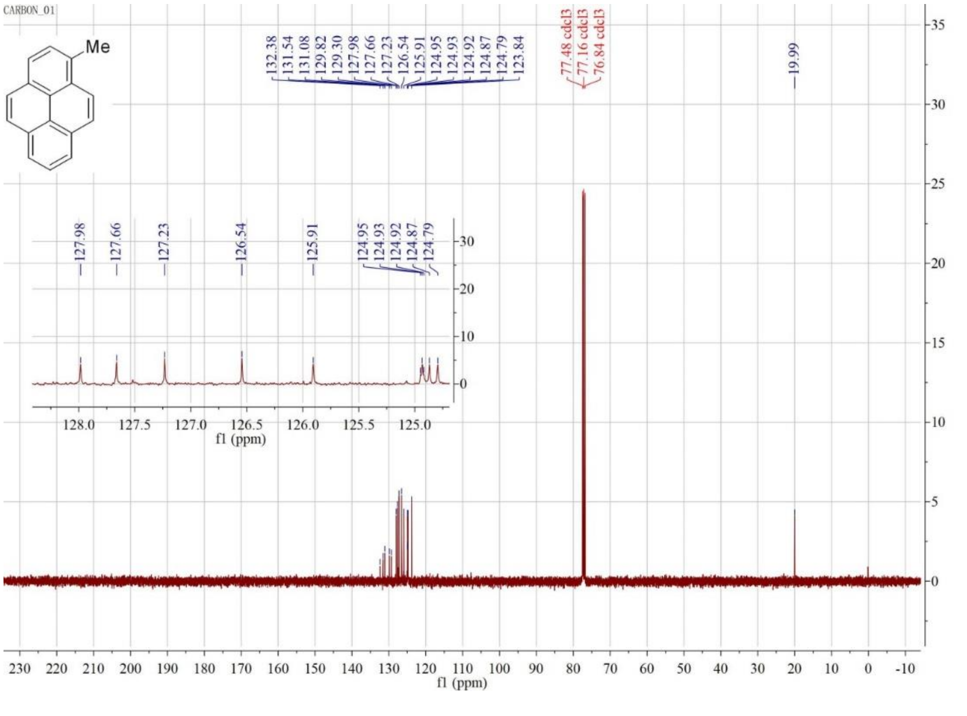

^{1}H NMR spectra of **2m**:

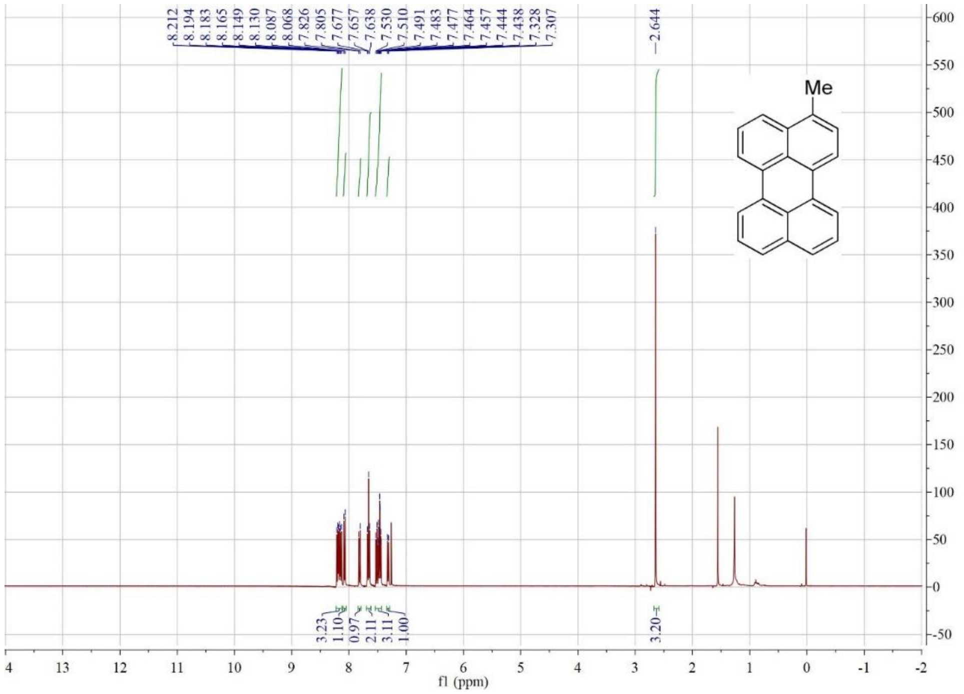

^{13}C NMR spectra of **2m**:

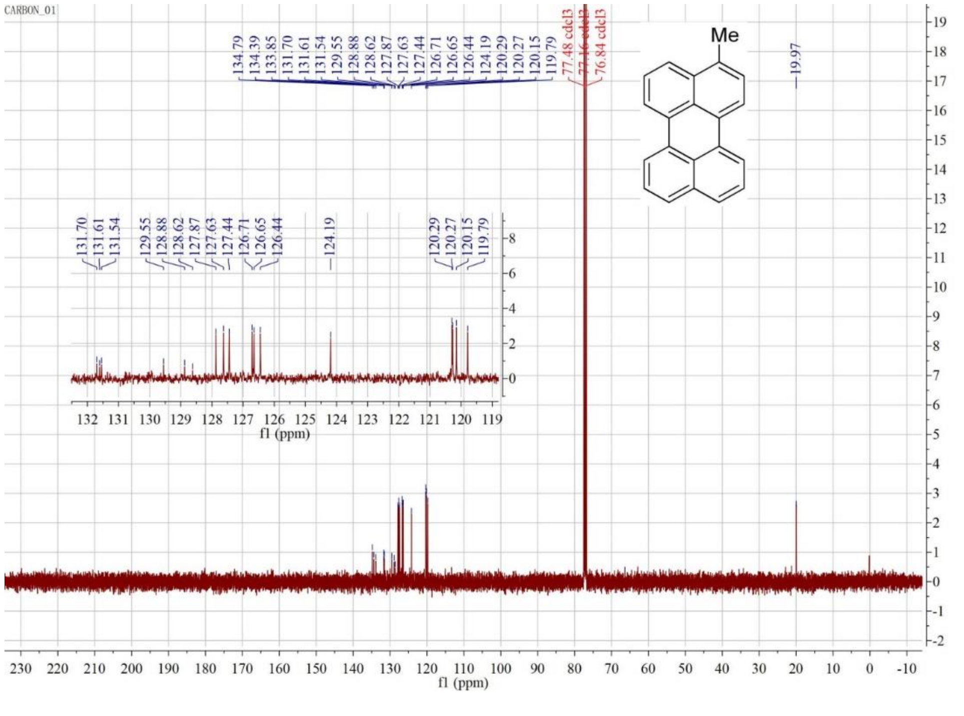

^{1}H NMR spectra of **2n**:

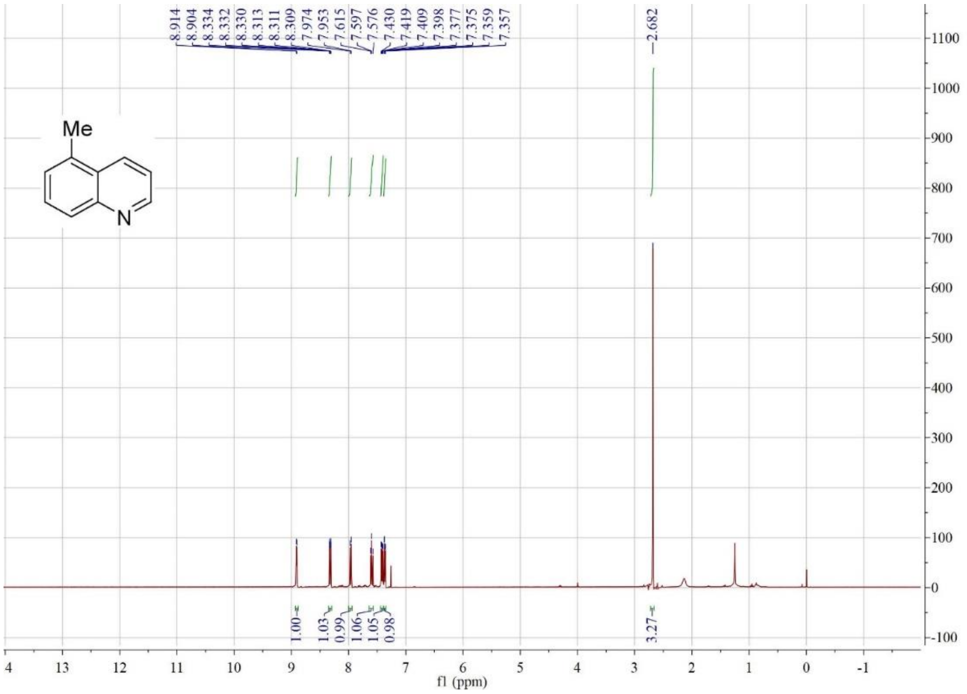

^{13}C NMR spectra of **2n**:

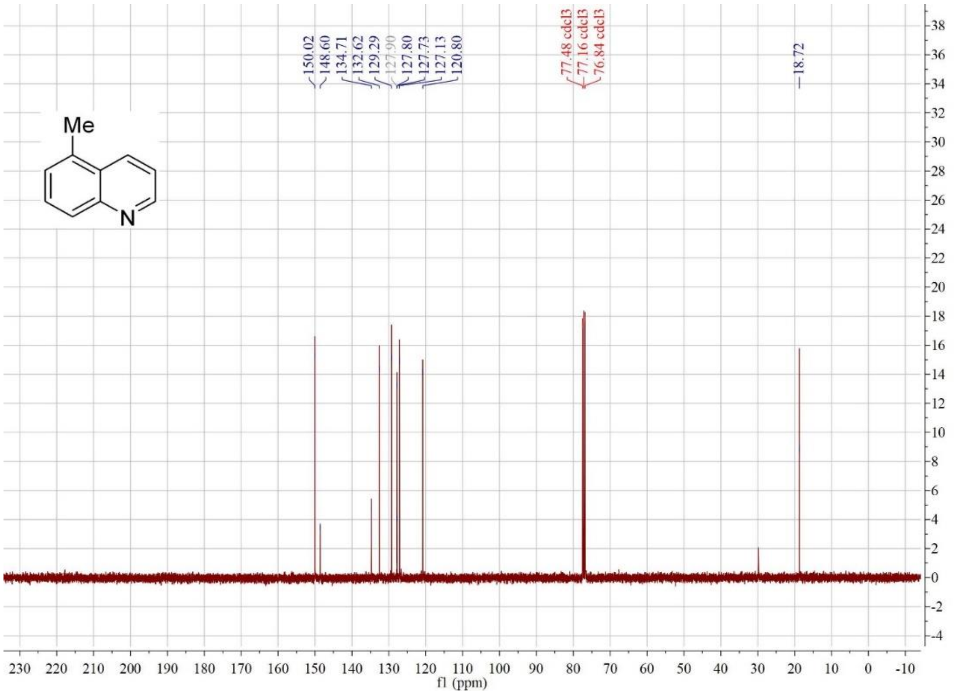

^{1}H NMR spectra of **2o**:

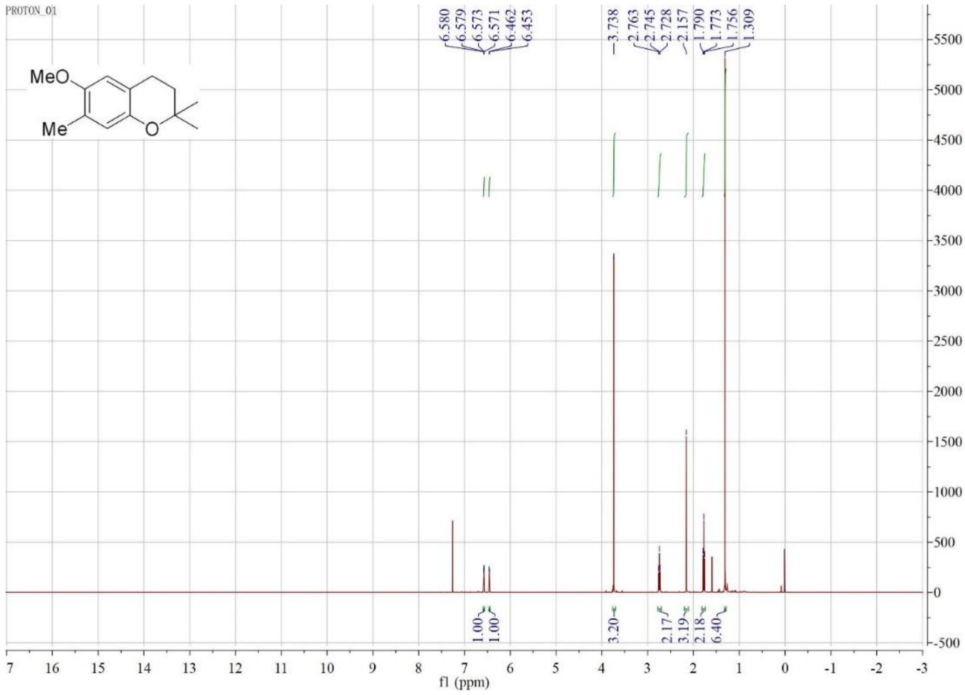

^{13}C NMR spectra of **2o**:

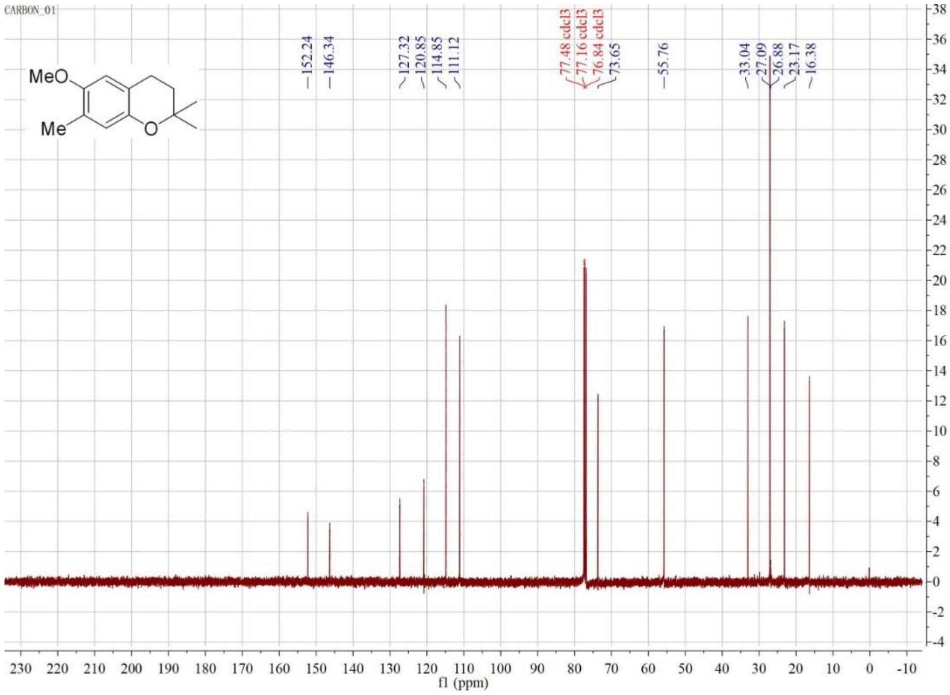

^{1}H NMR spectra of **2p**:

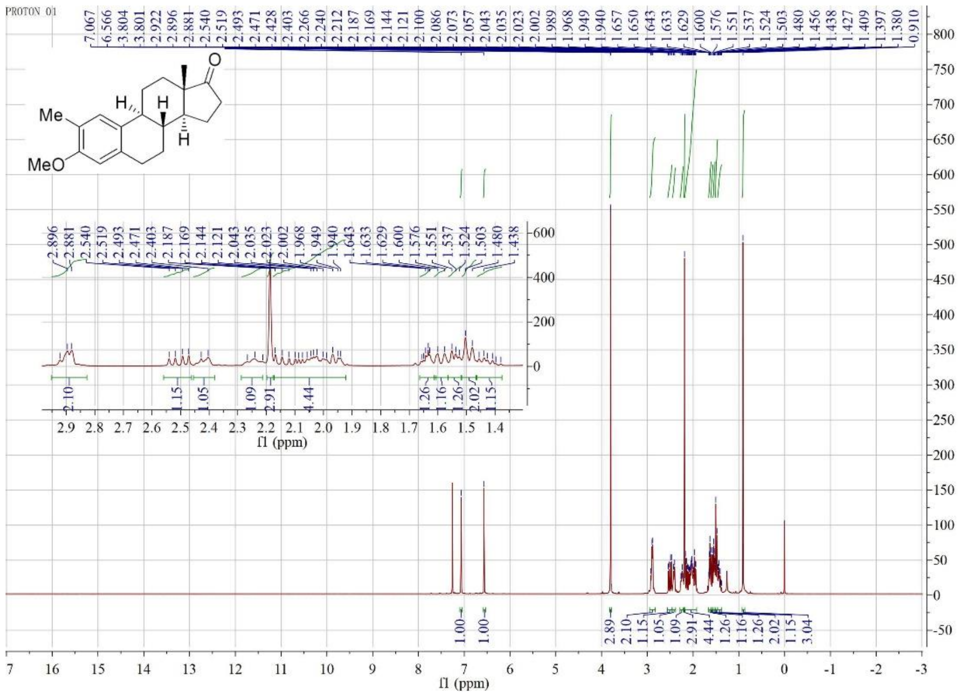

^{13}C NMR spectra of **2p**:

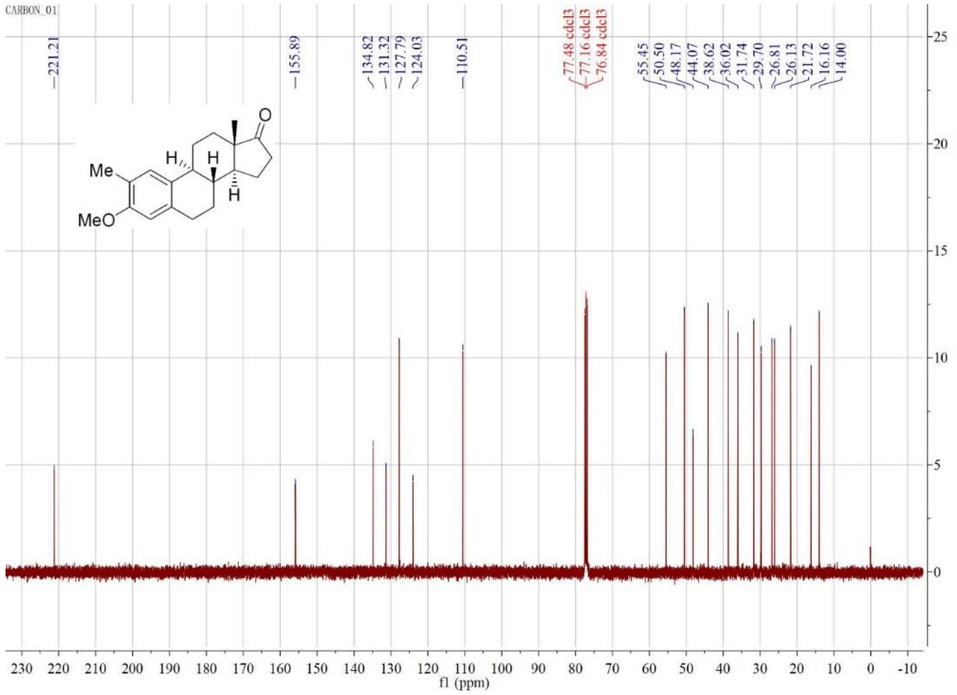

^1H NMR spectra of **2q**:

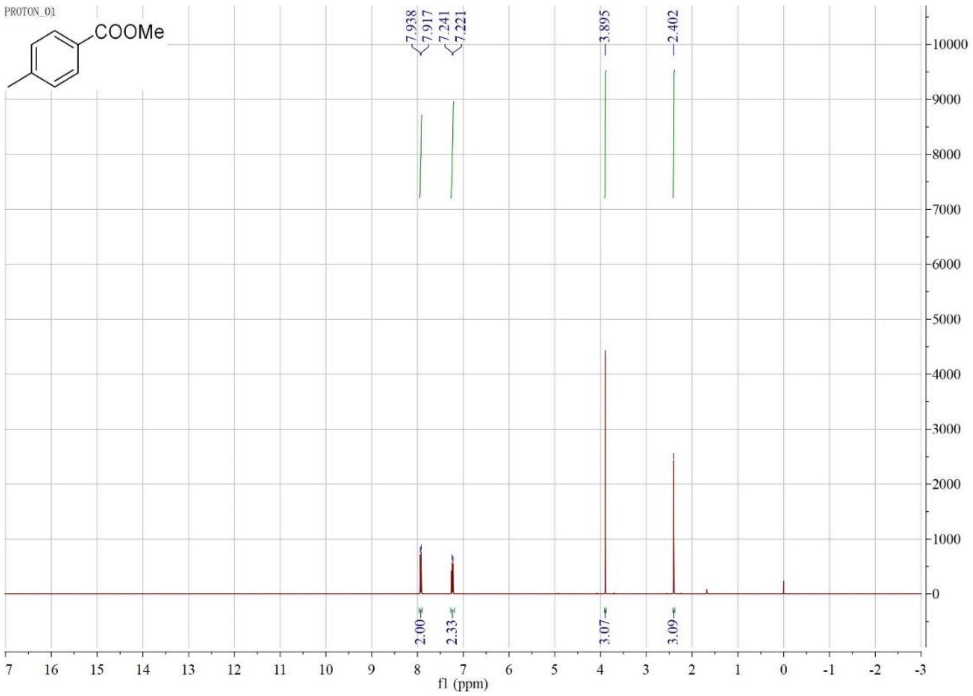

^{13}C NMR spectra of **2q**:

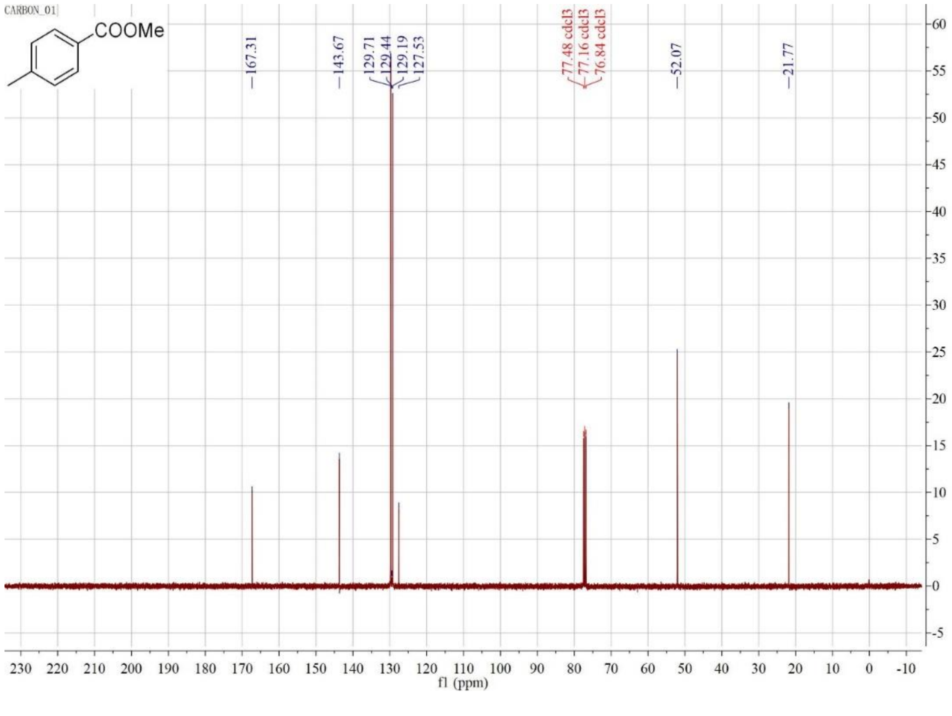

^{1}H NMR spectra of **2r**:

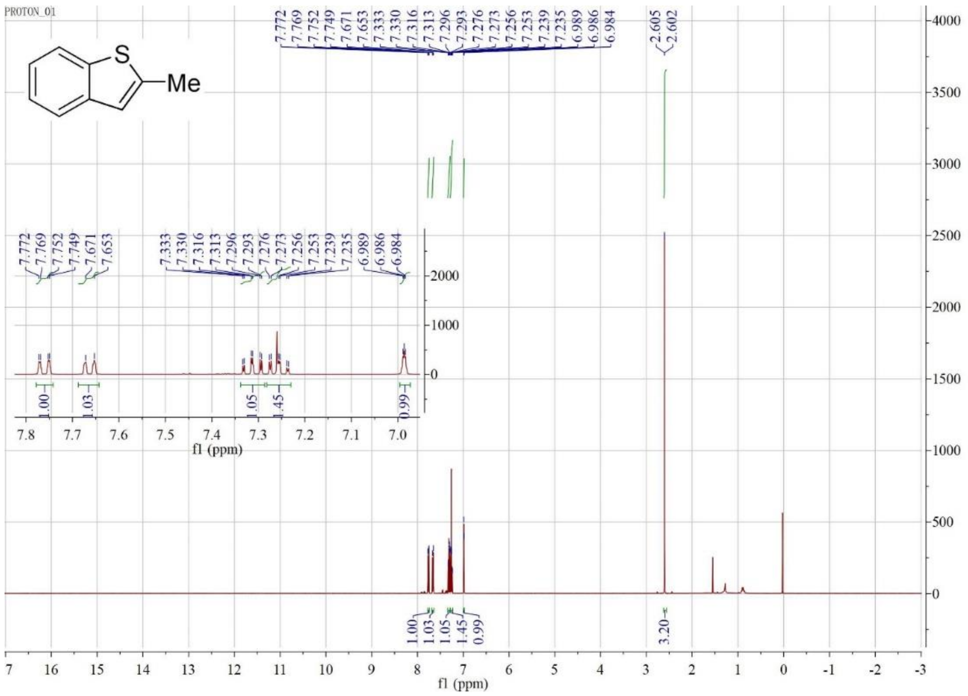

^{13}C NMR spectra of **2r**:

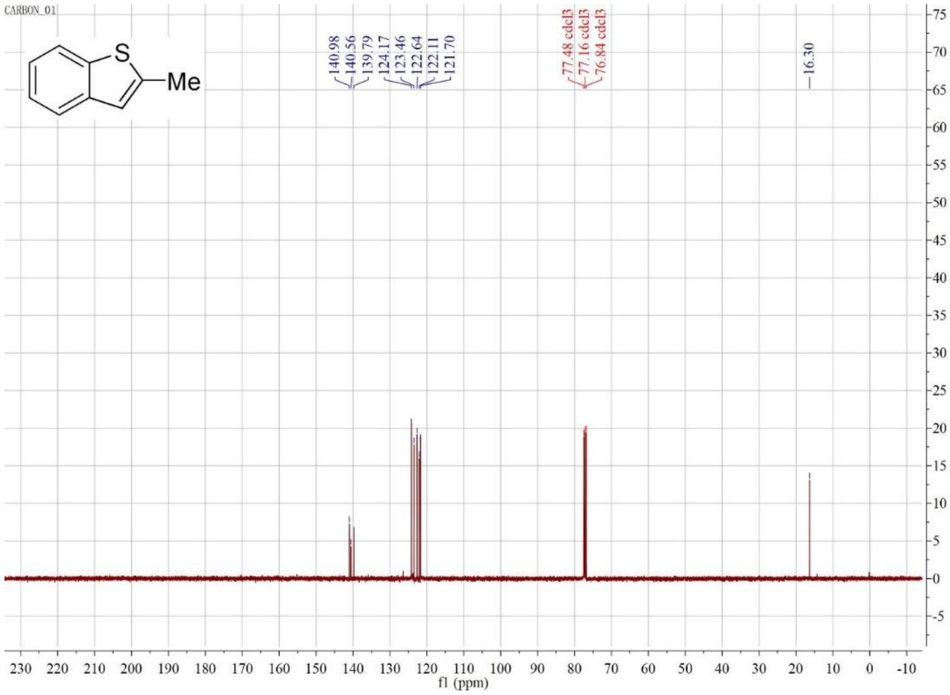

^{1}H NMR spectra of **2s**:

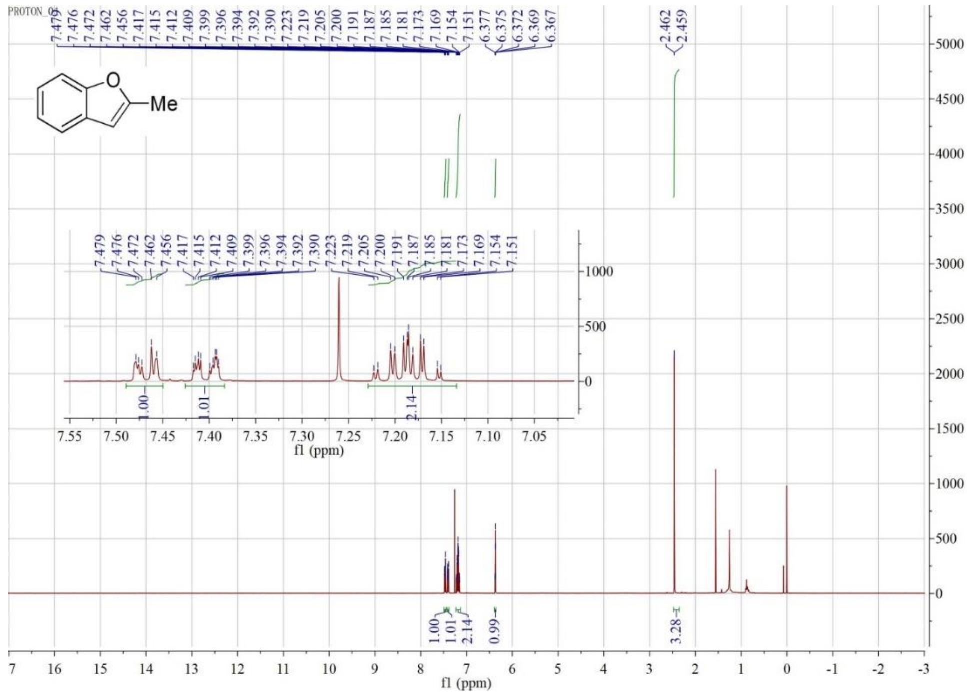

^{13}C NMR spectra of **2s**:

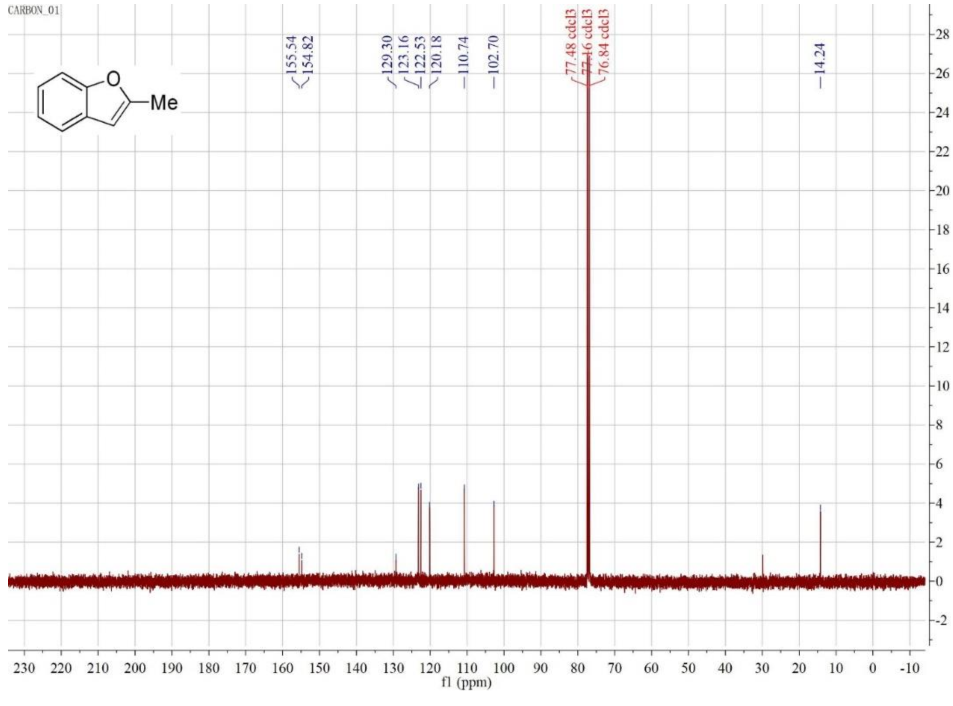

^{1}H NMR spectra of **2t**:

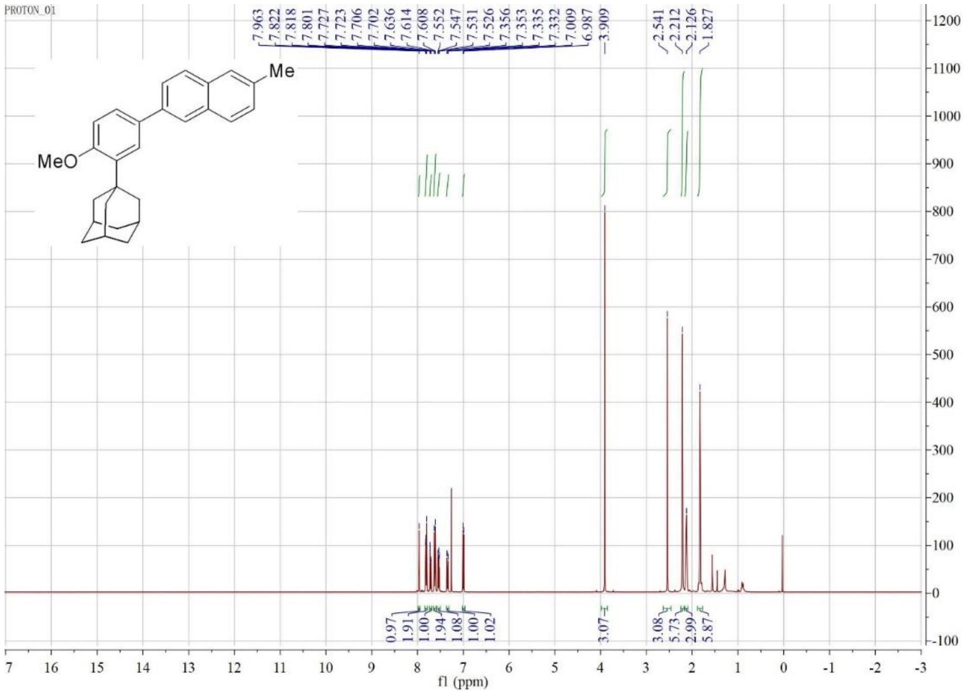

^{13}C NMR spectra of **2t**:

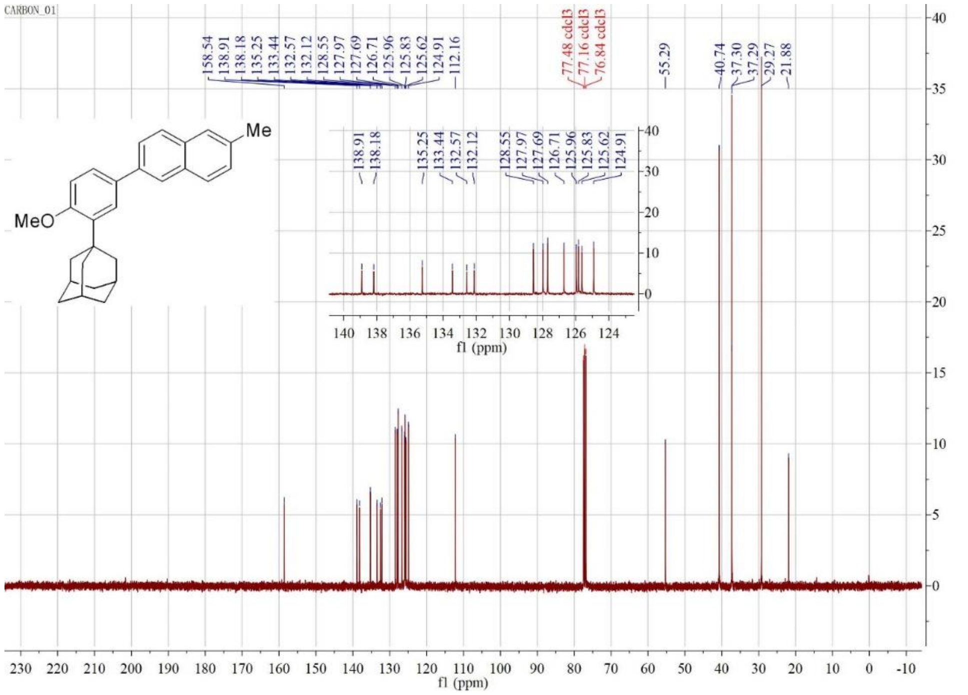

^{1}H NMR spectra of **3a**:

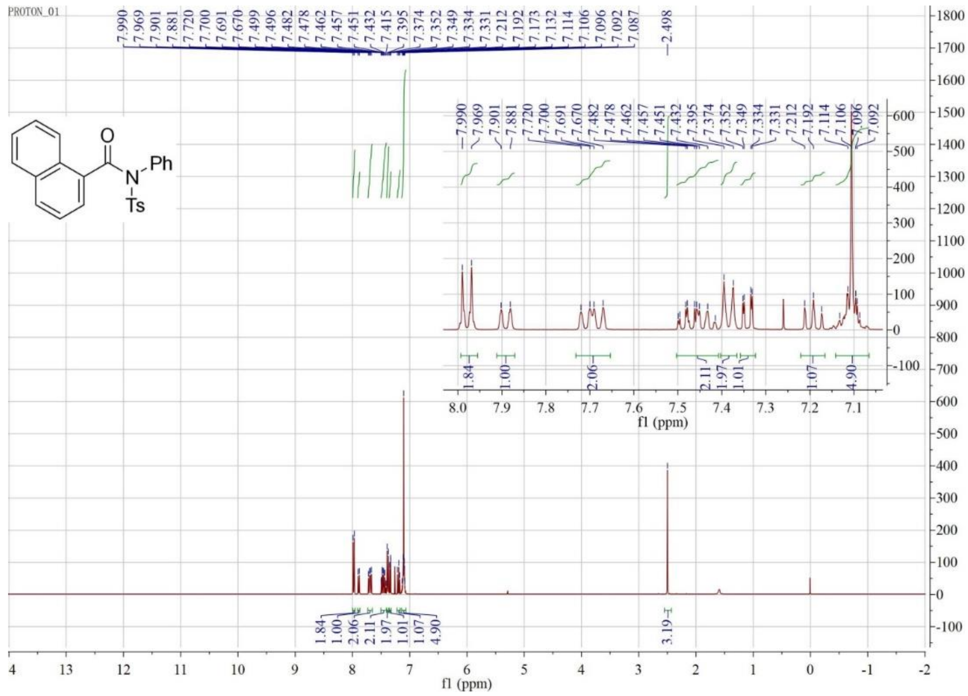

^{13}C NMR spectra of **3a**:

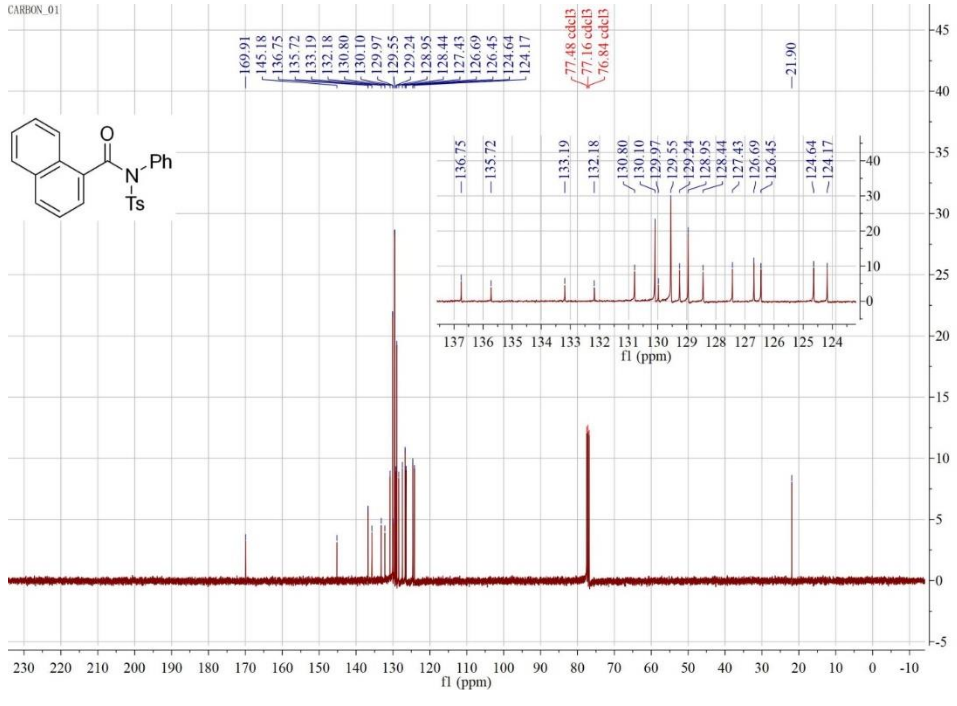

^{1}H NMR spectra of **3b**:

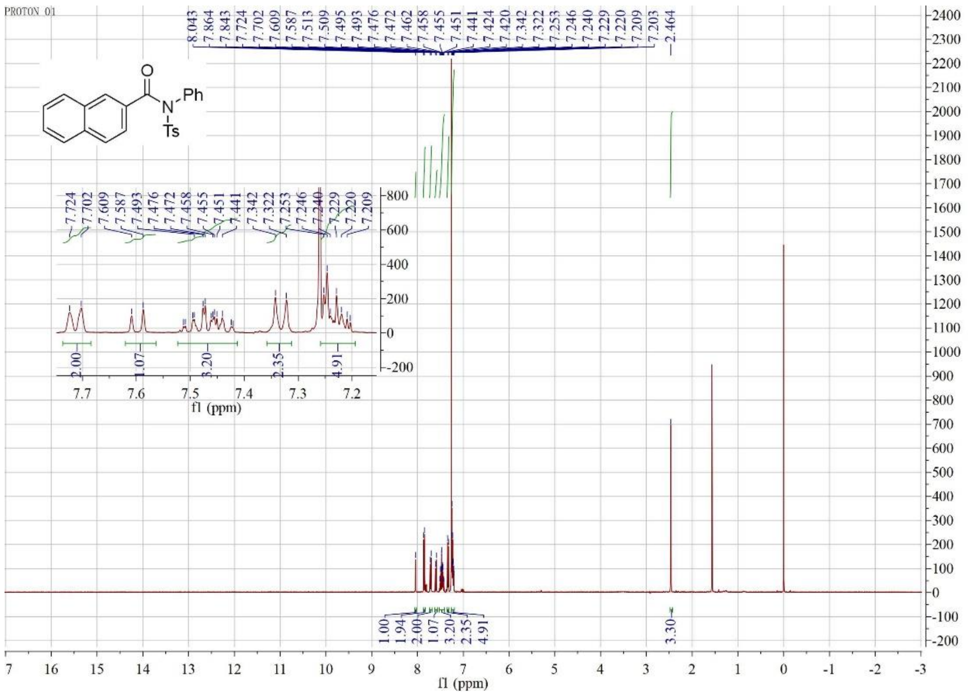

^{13}C NMR spectra of **3b**:

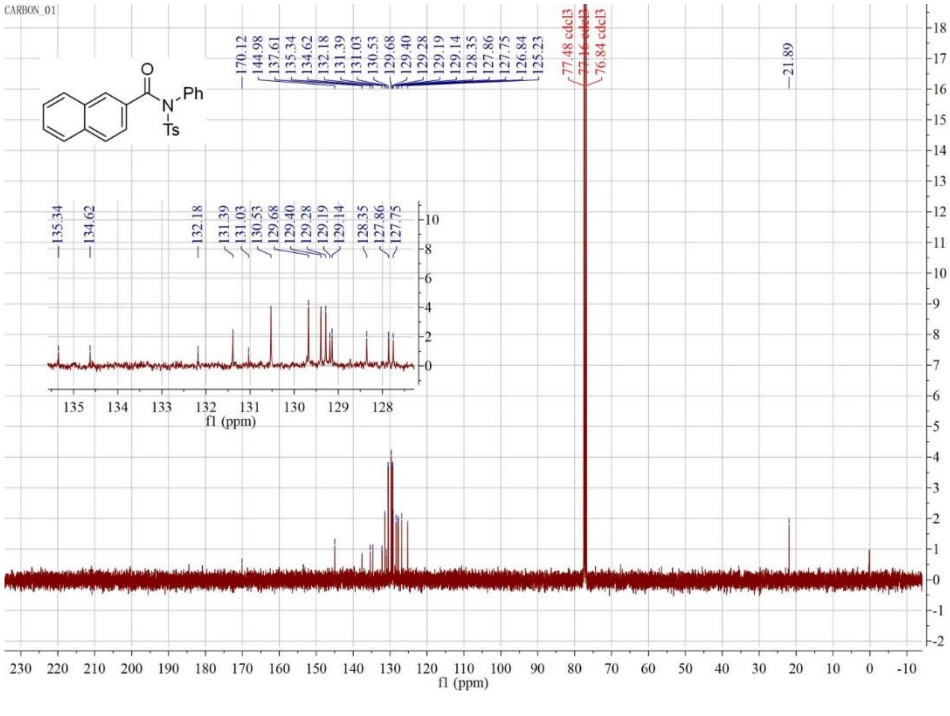

^{1}H NMR spectra of **3c**:

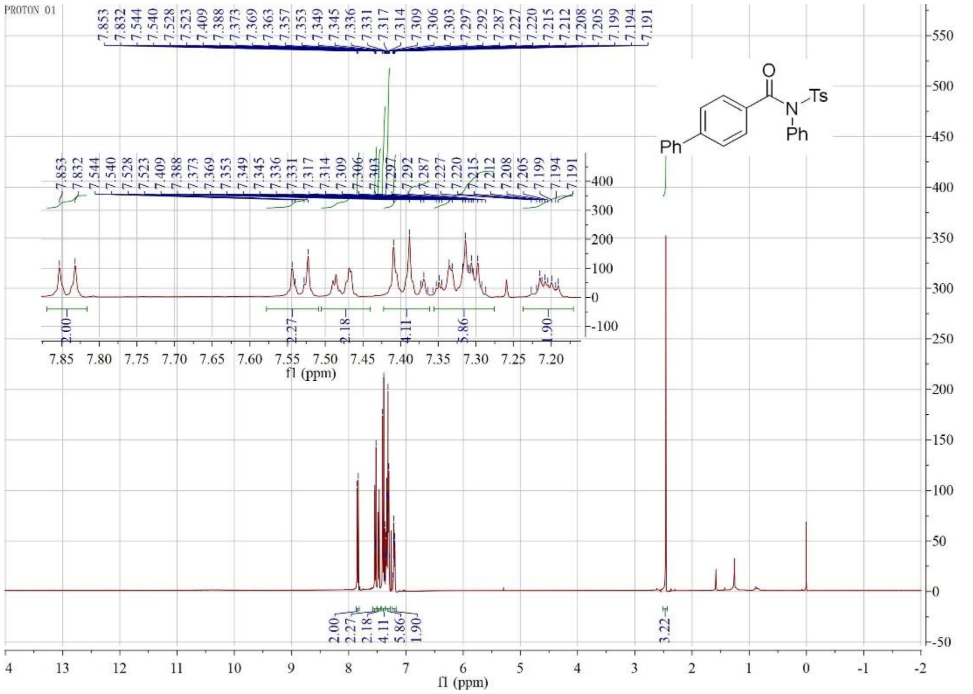

^{13}C NMR spectra of **3c**:

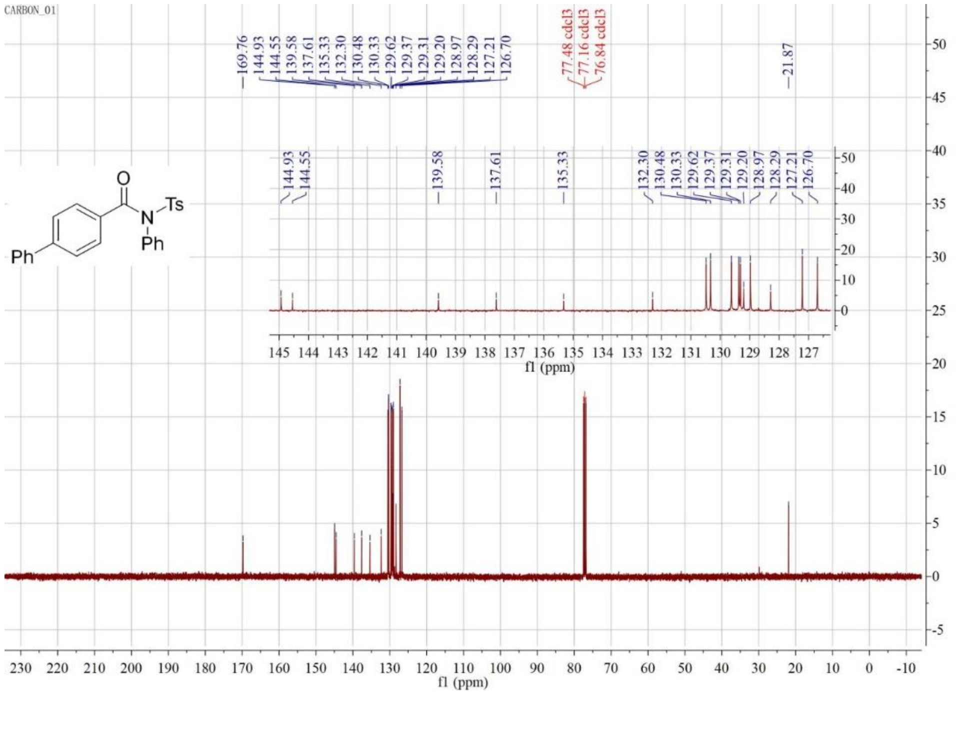

^{1}H NMR spectra of **3d**:

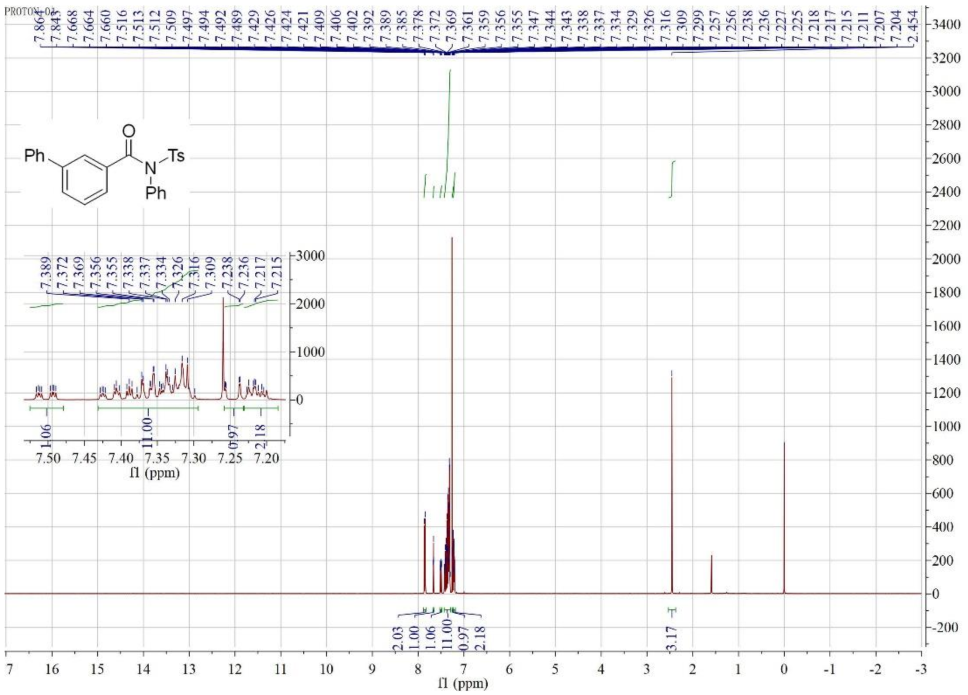

^{13}C NMR spectra of **3d**:

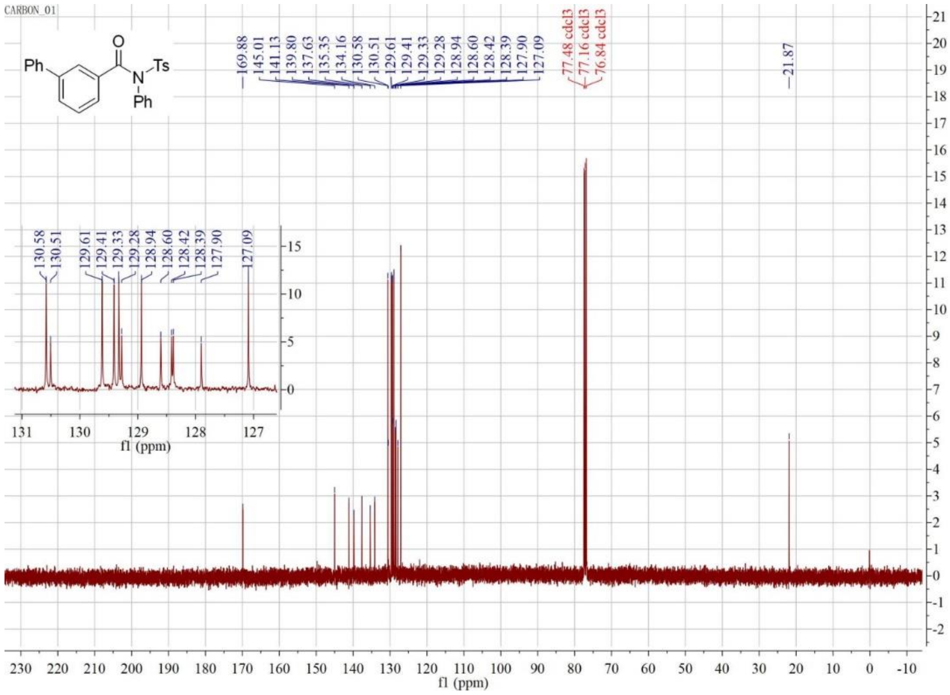

^{1}H NMR spectra of **3e**:

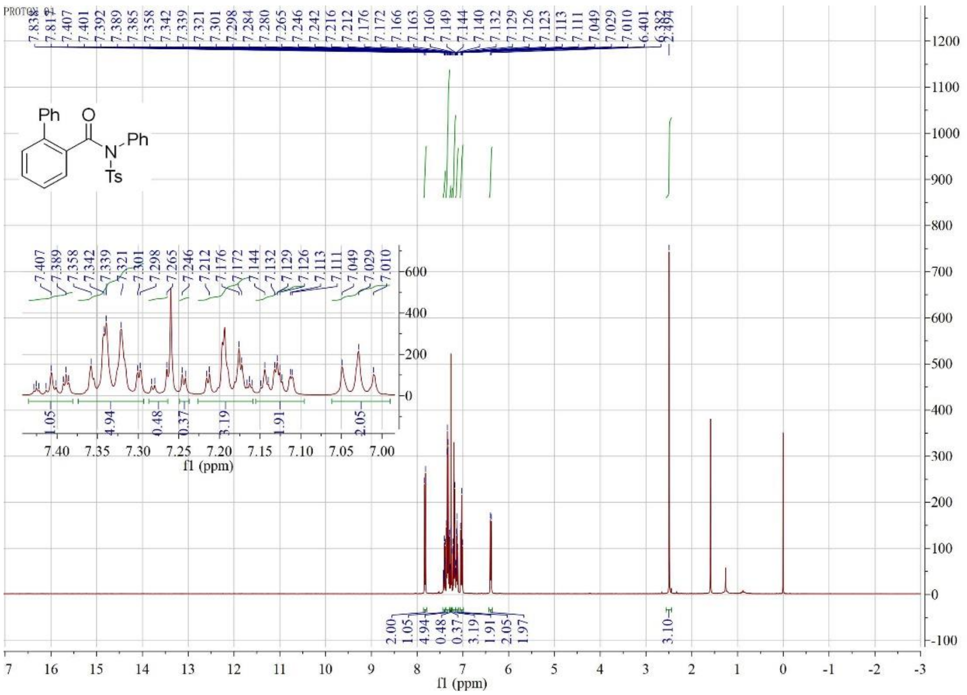

^{13}C NMR spectra of **3e**:

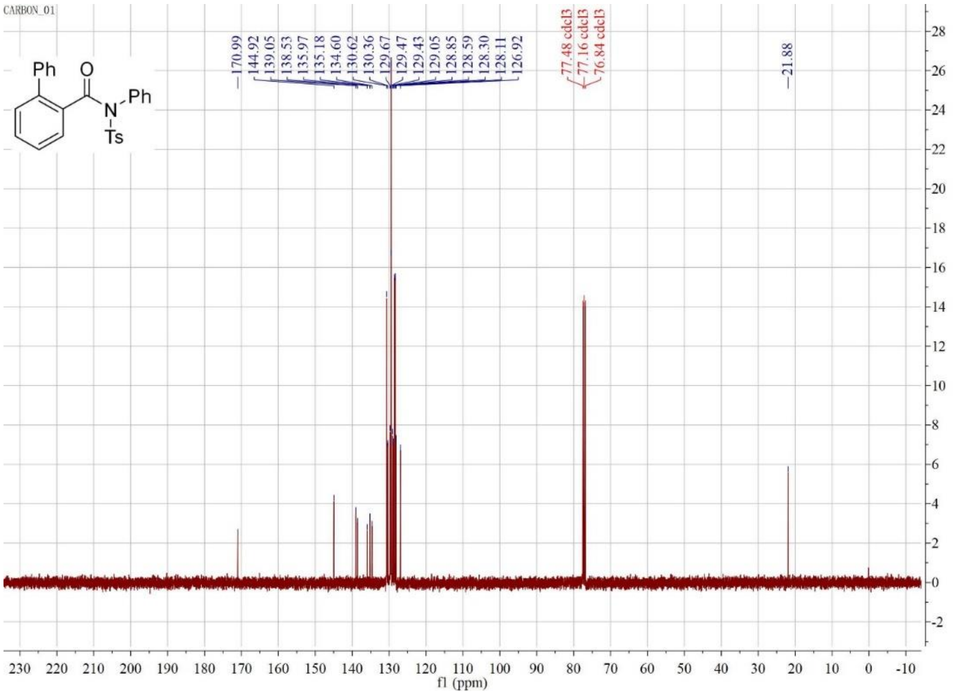

^{1}H NMR spectra of **3f**:

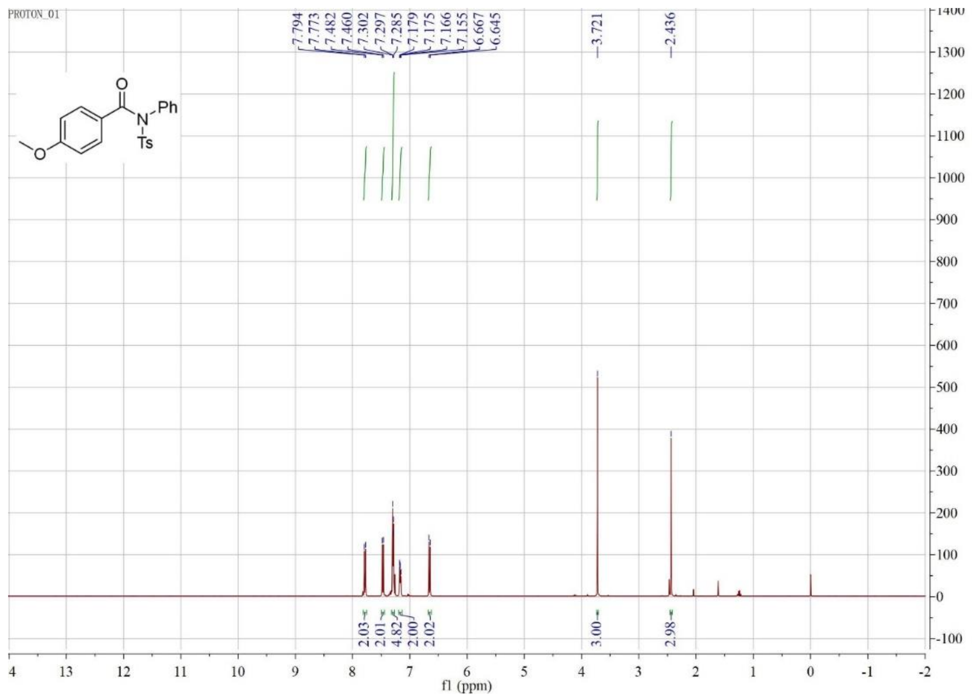

^{13}C NMR spectra of **3f**:

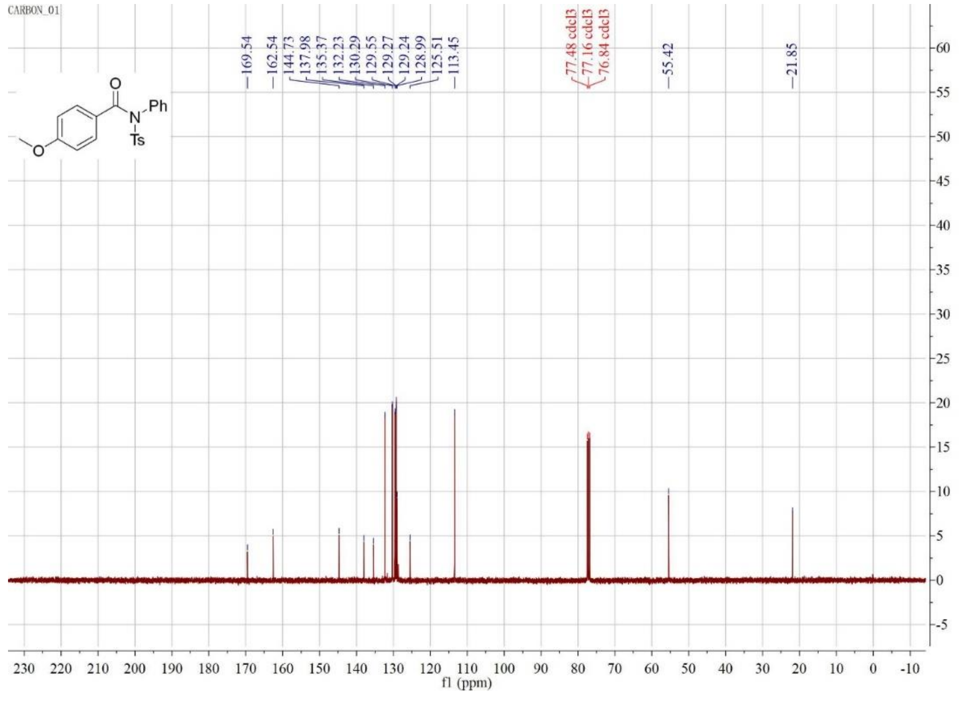

^{1}H NMR spectra of **3g**:

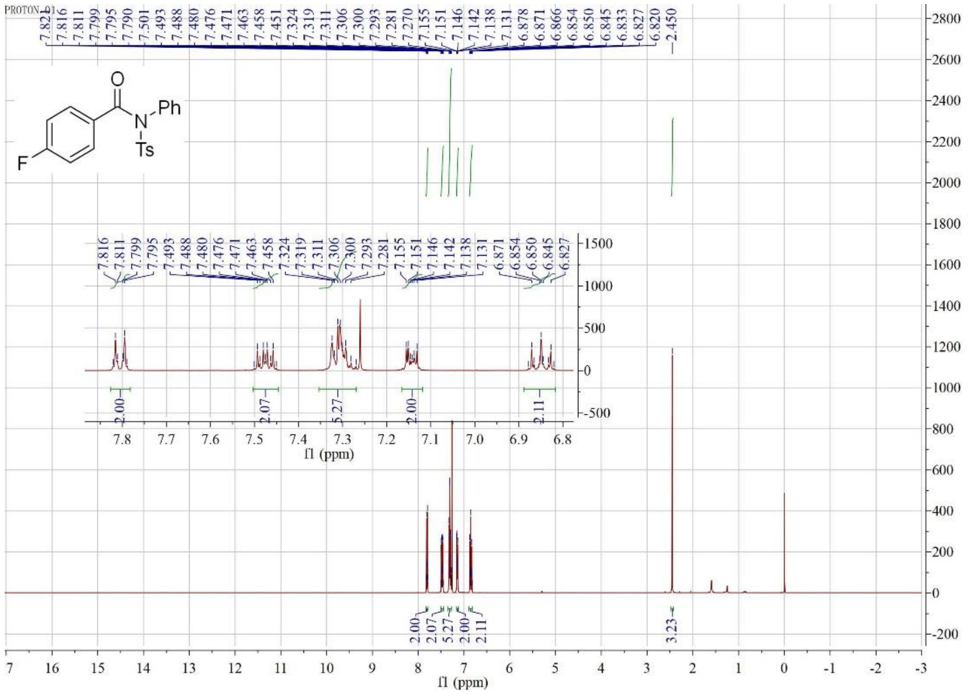

^{13}C NMR spectra of **3g**:

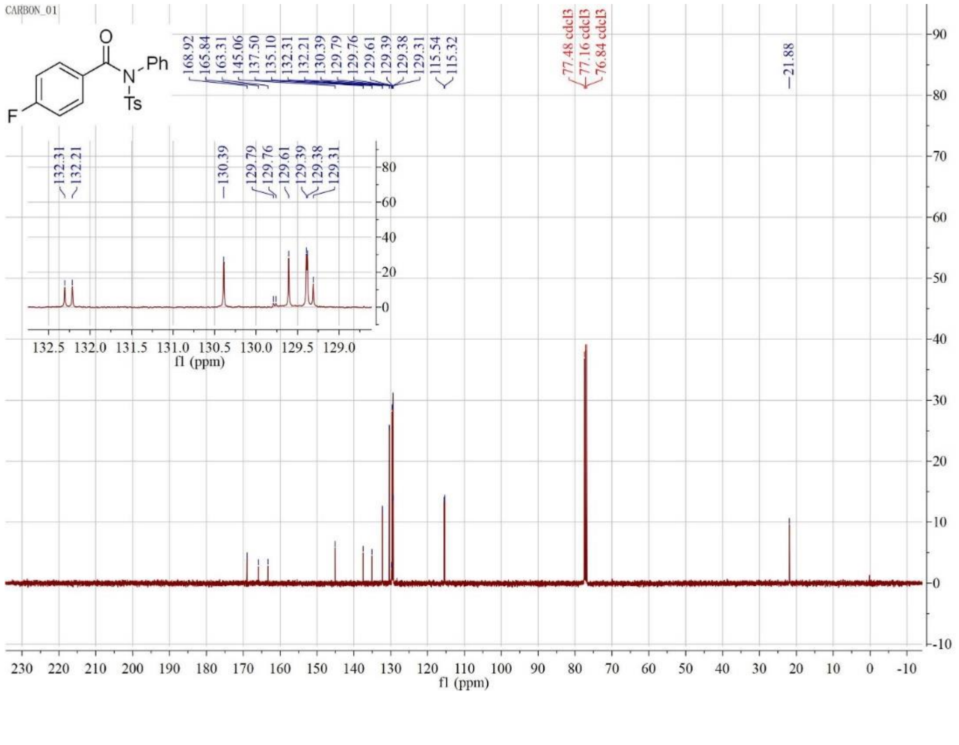

^{19}F NMR spectra of **3g**:

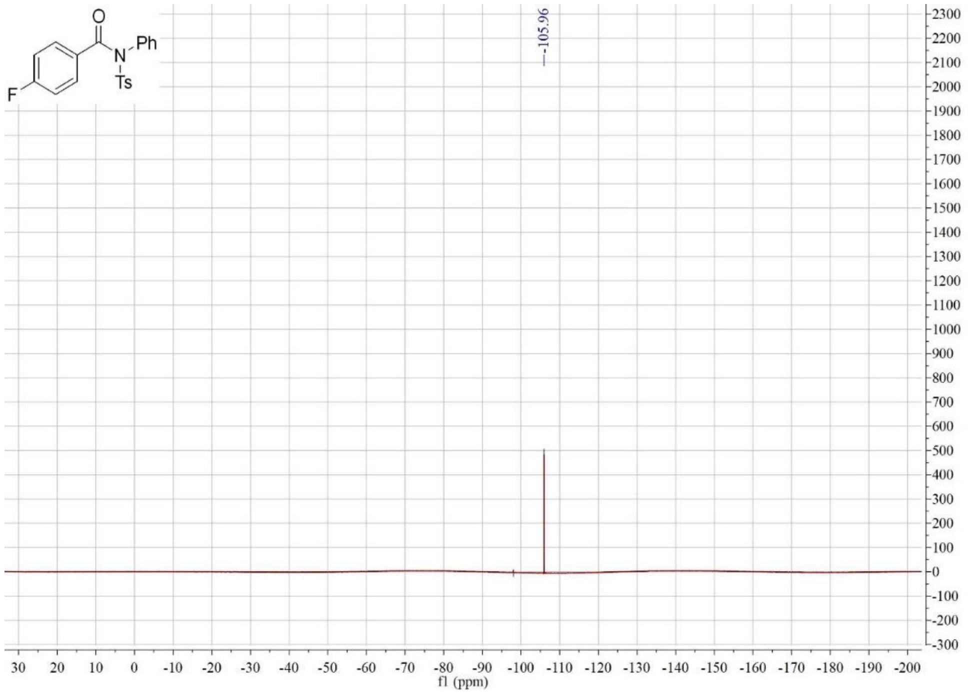

^{1}H NMR spectra of **3h**:

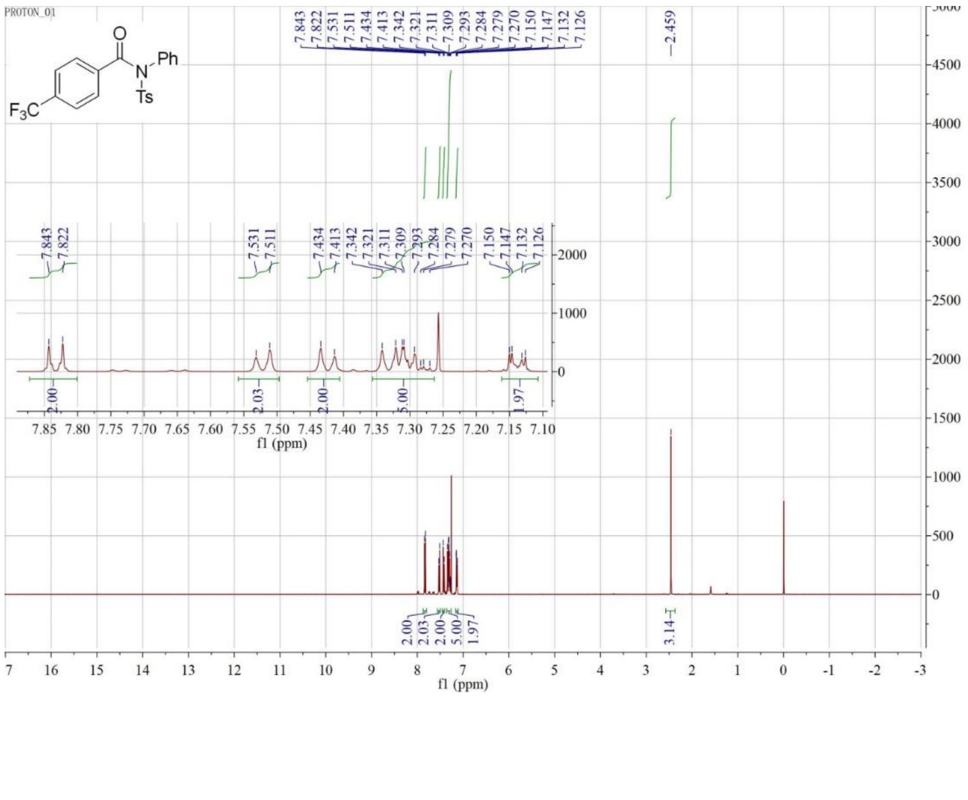

^{13}C NMR spectra of **3h**:

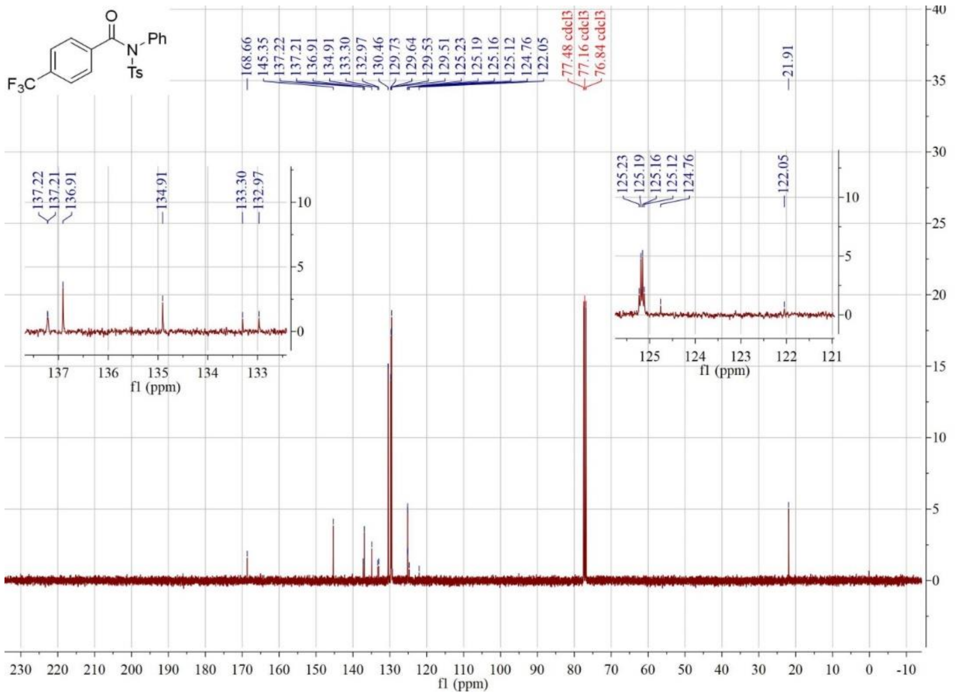

^{19}F NMR spectra of **3h**:

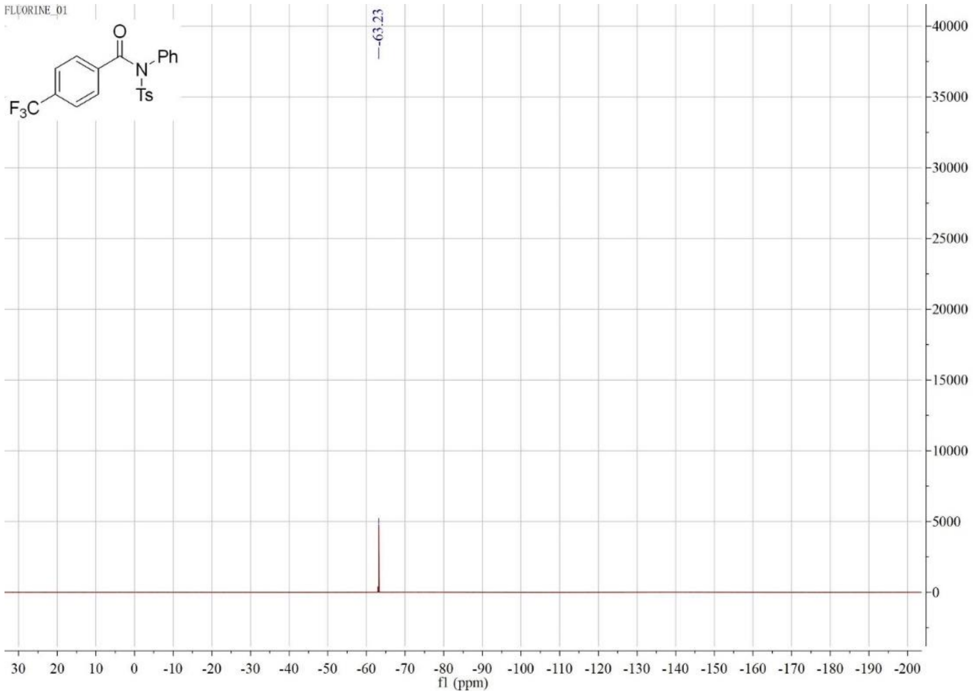

^{1}H NMR spectra of **3q**:

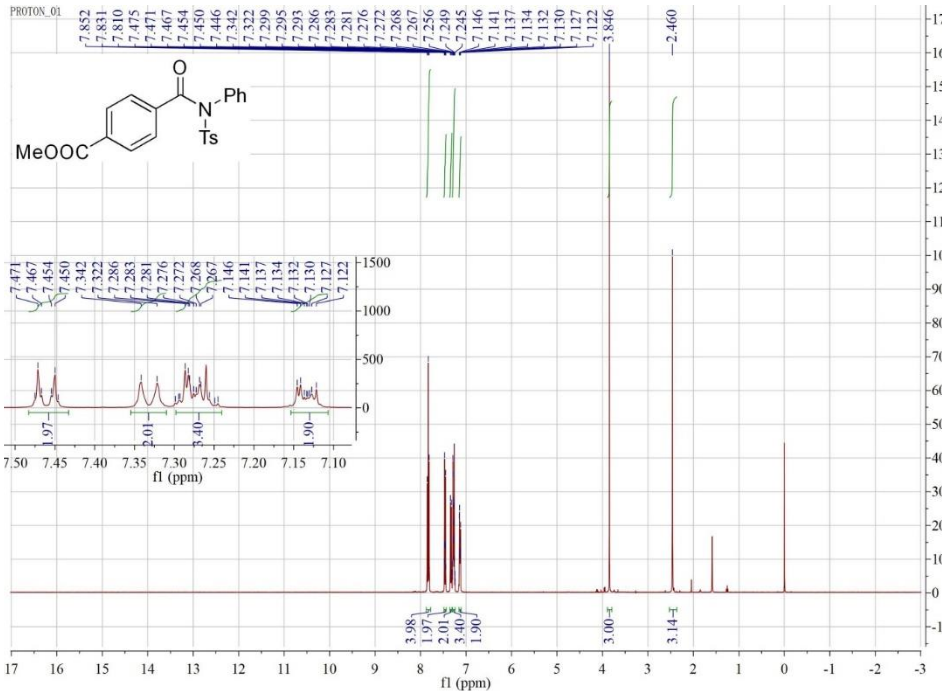

^{13}C NMR spectra of **3q**:

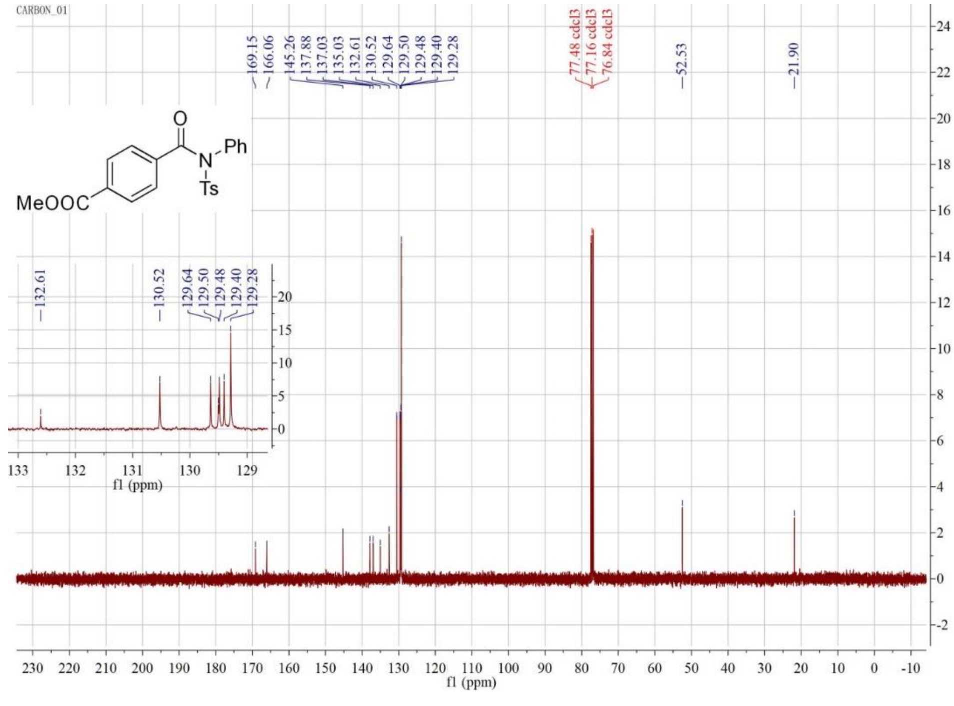

^{1}H NMR spectra of **3r**:

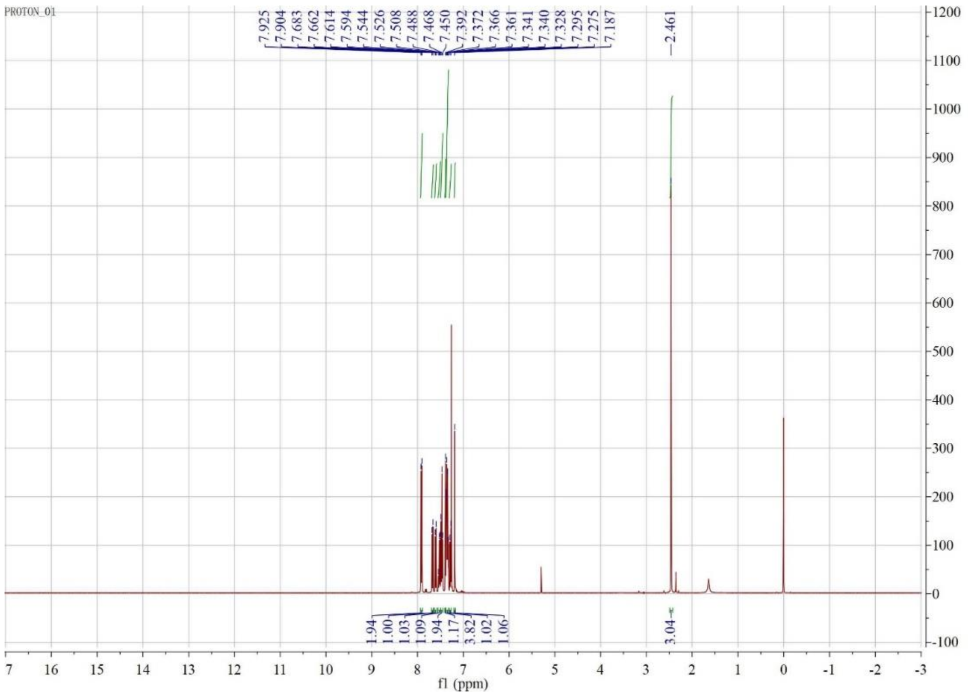

^{13}C NMR spectra of **3r**:

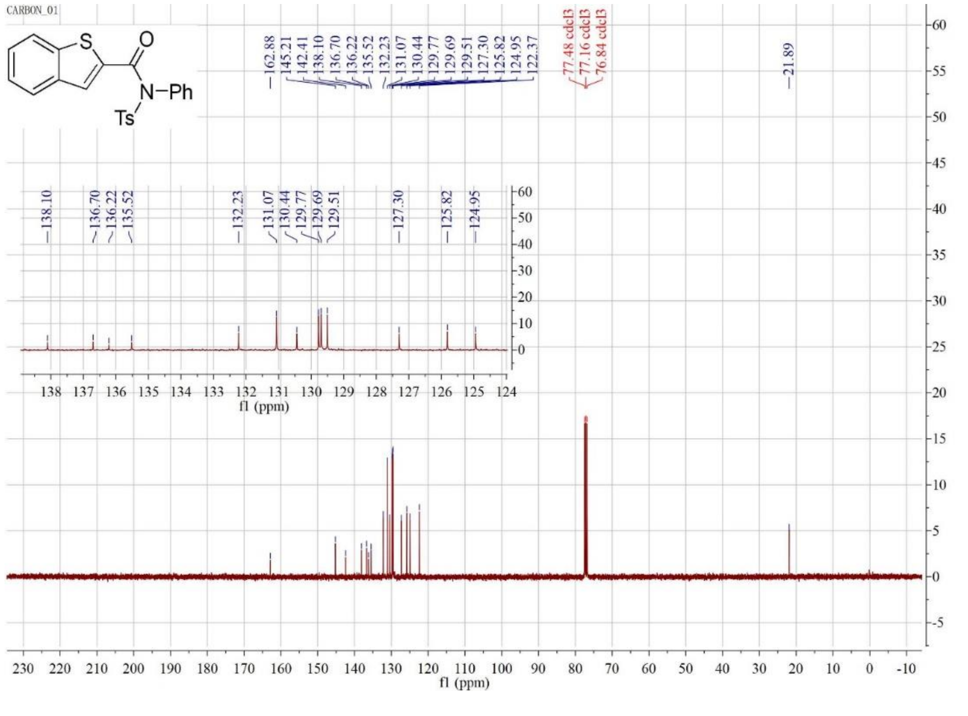

^{1}H NMR spectra of **3s**:

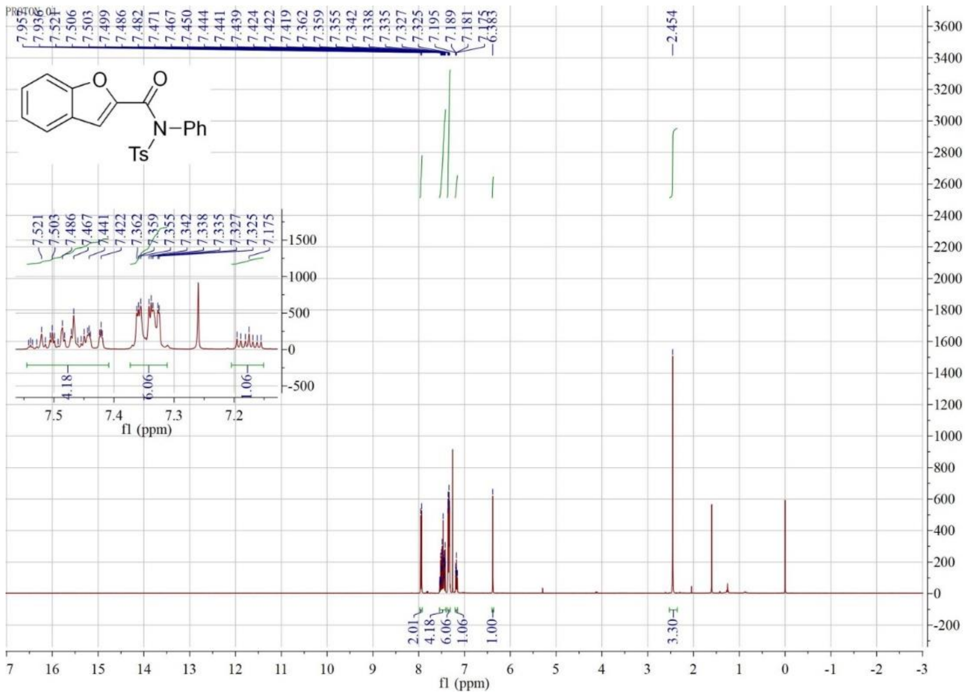

^{13}C NMR spectra of **3s**:

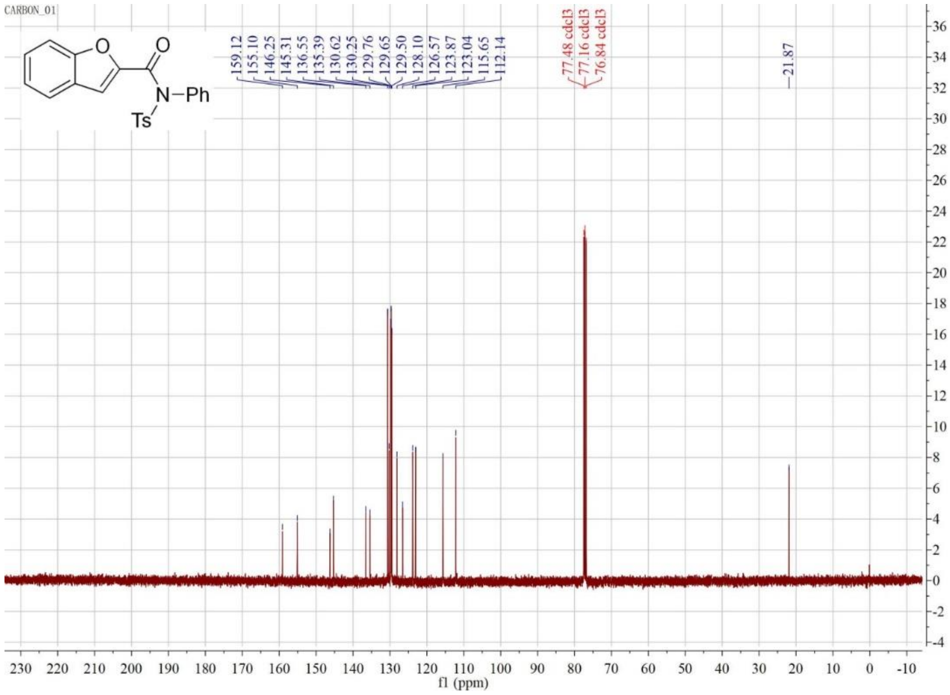

^{1}H NMR spectra of **4a**:

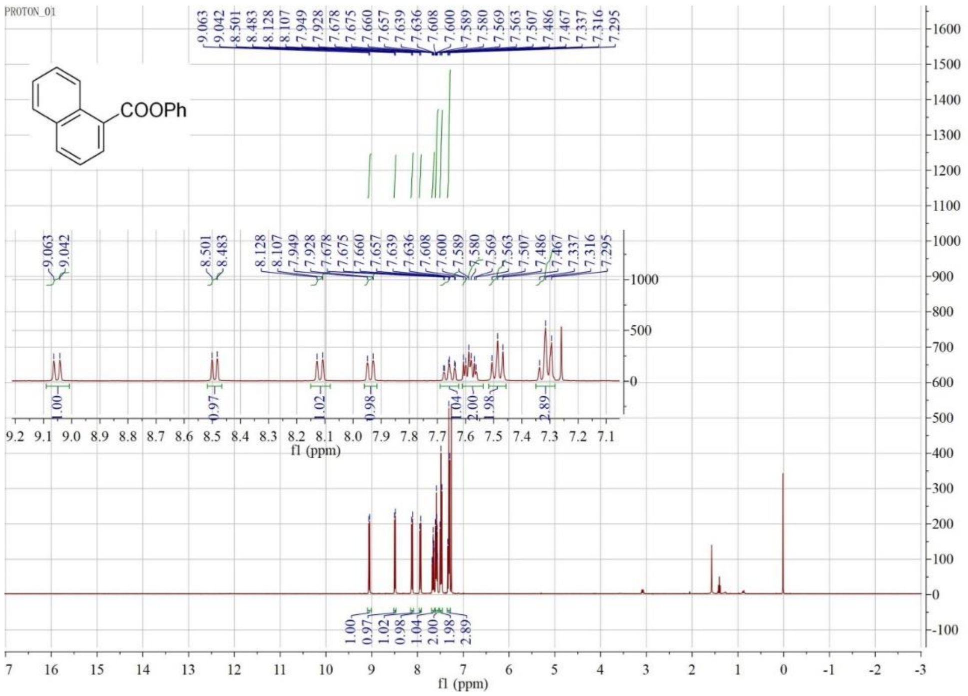

^{13}C NMR spectra of **4a**:

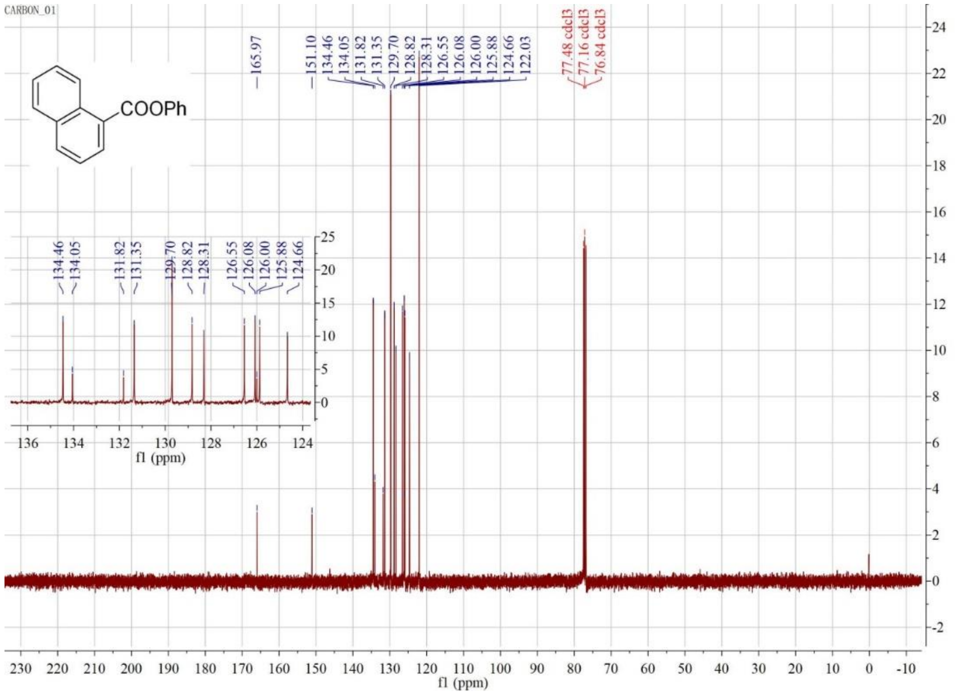

^{1}H NMR spectra of **4b**:

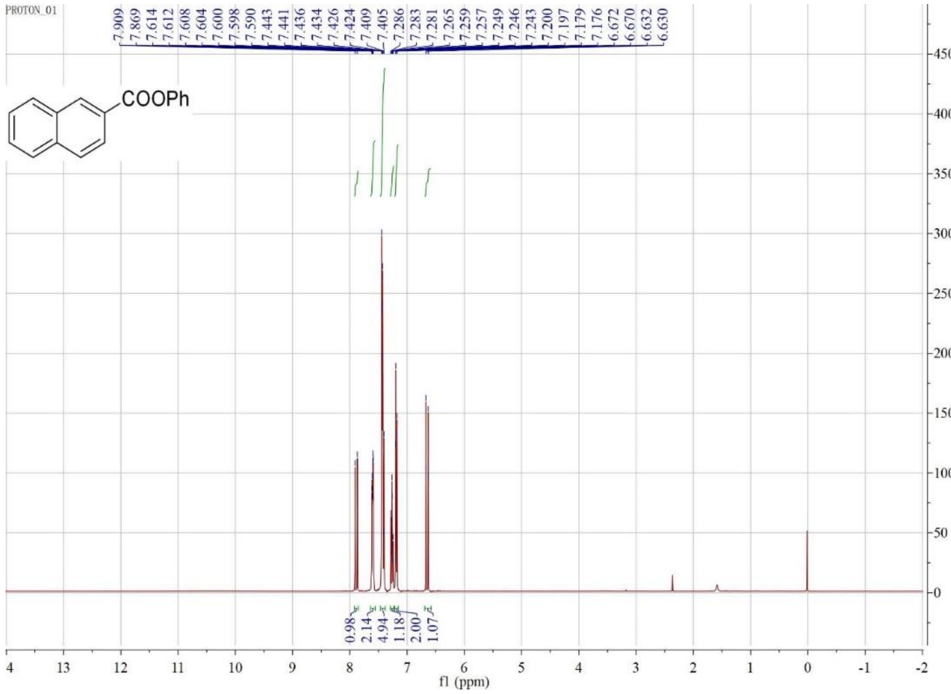

^{13}C NMR spectra of **4b**:

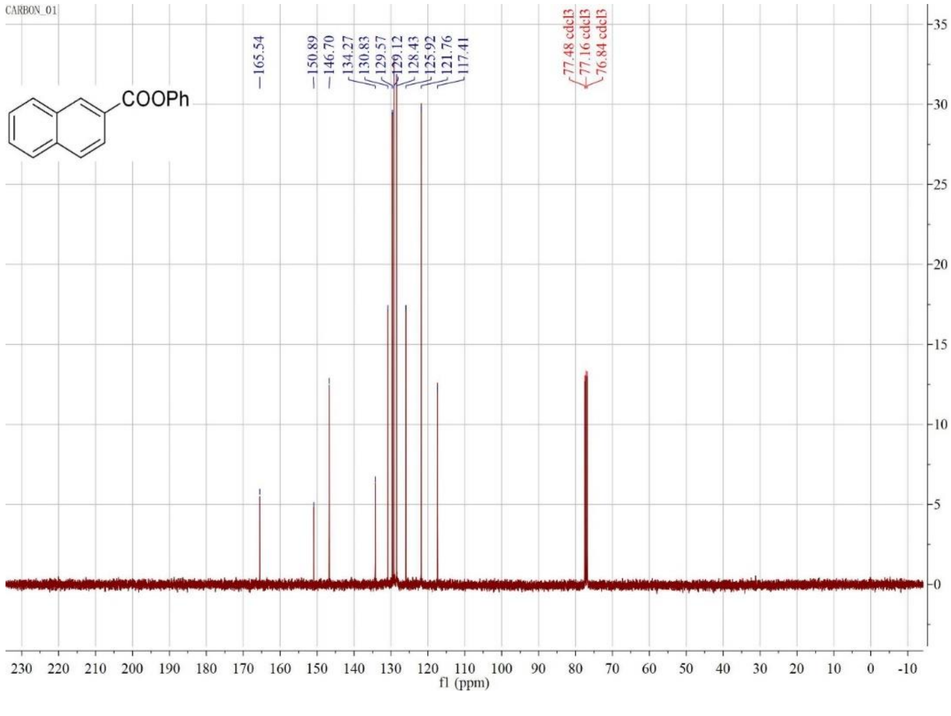

^{1}H NMR spectra of **4c**:

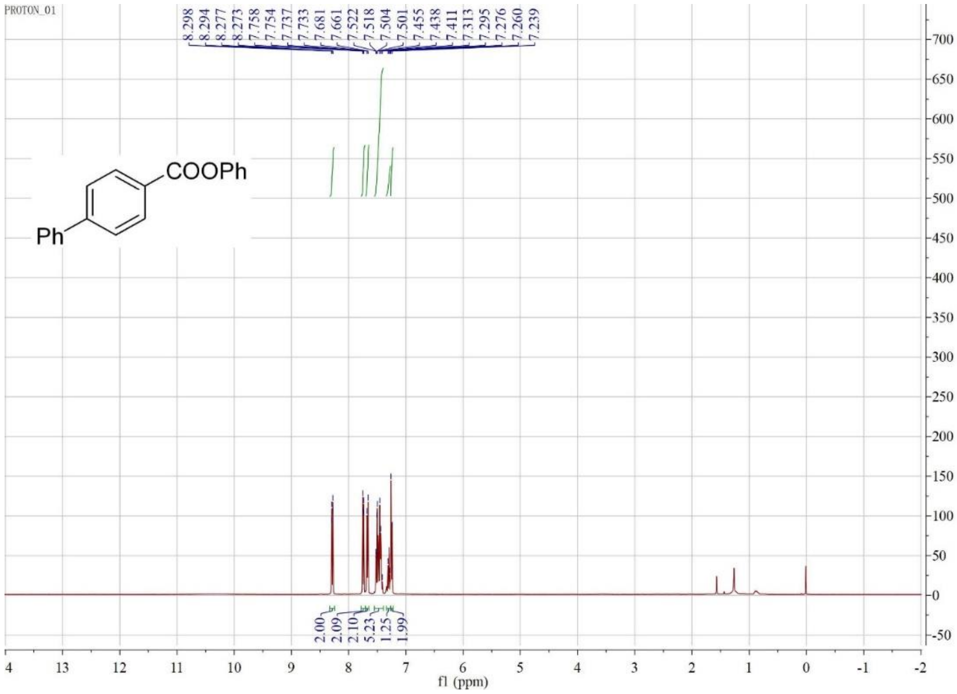

^{13}C NMR spectra of **4c**:

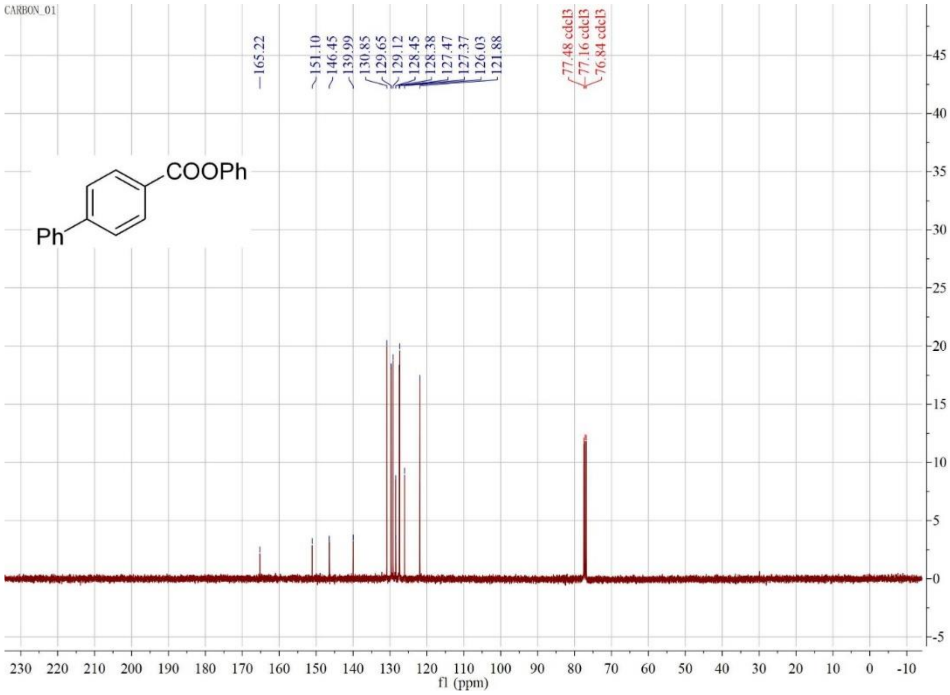

^{1}H NMR spectra of **4d**:

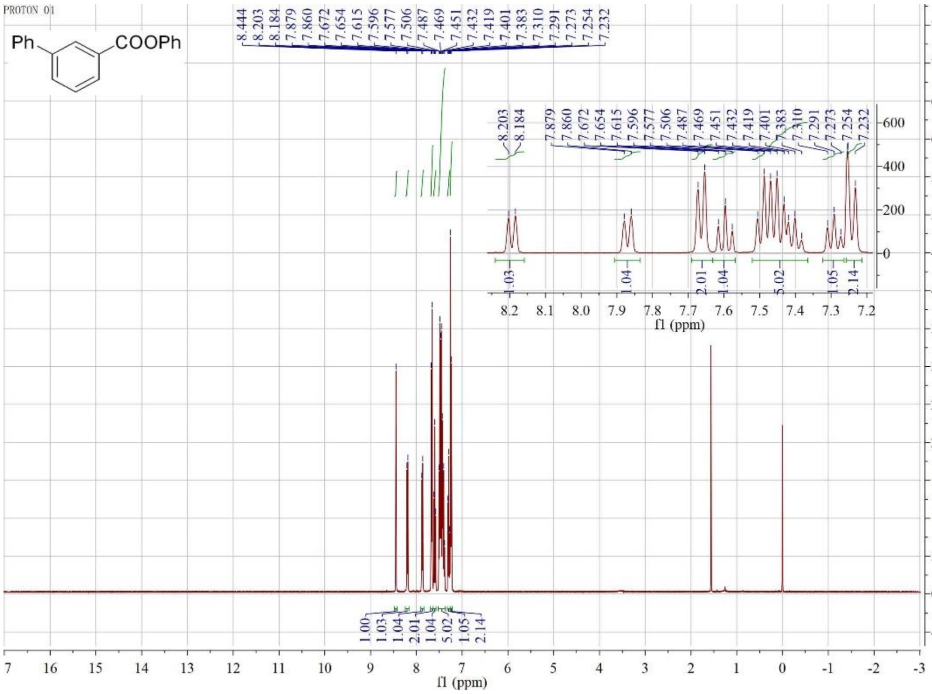

^{13}C NMR spectra of **4d**:

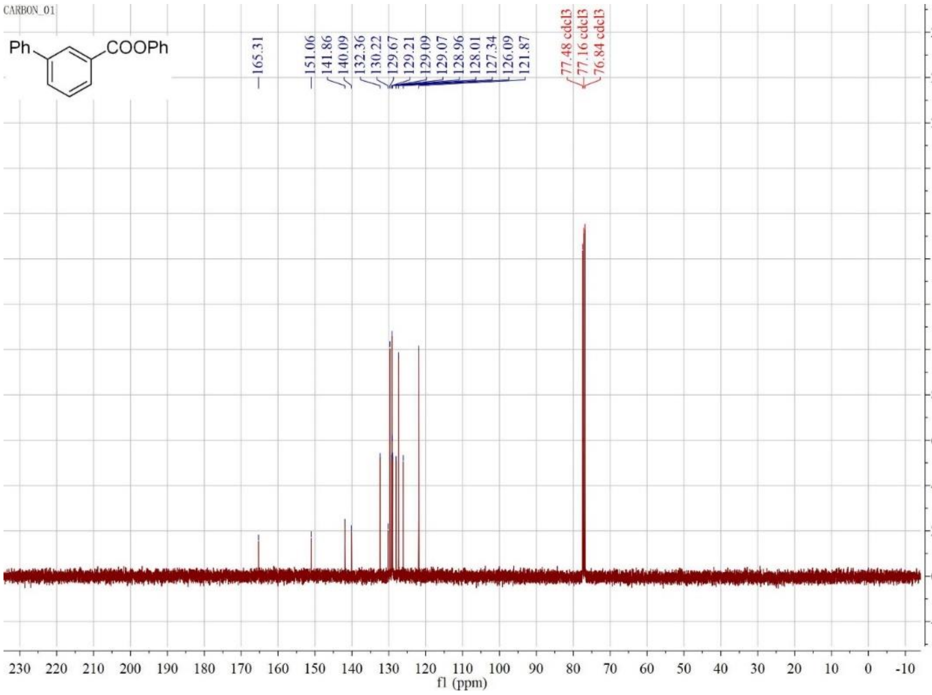

^{1}H NMR spectra of **4e**:

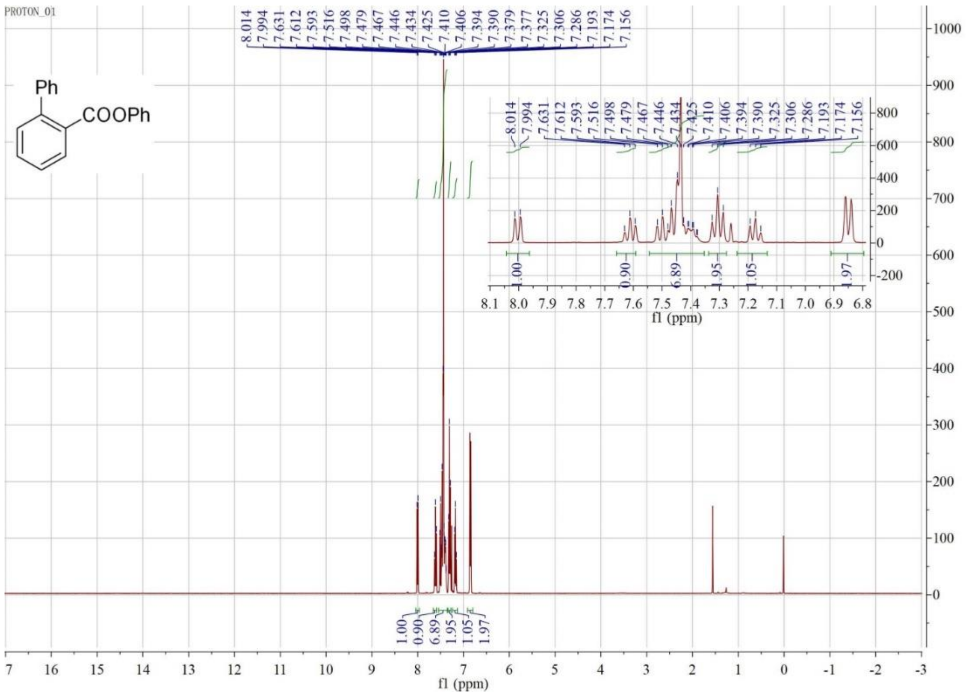

^{13}C NMR spectra of **4e**:

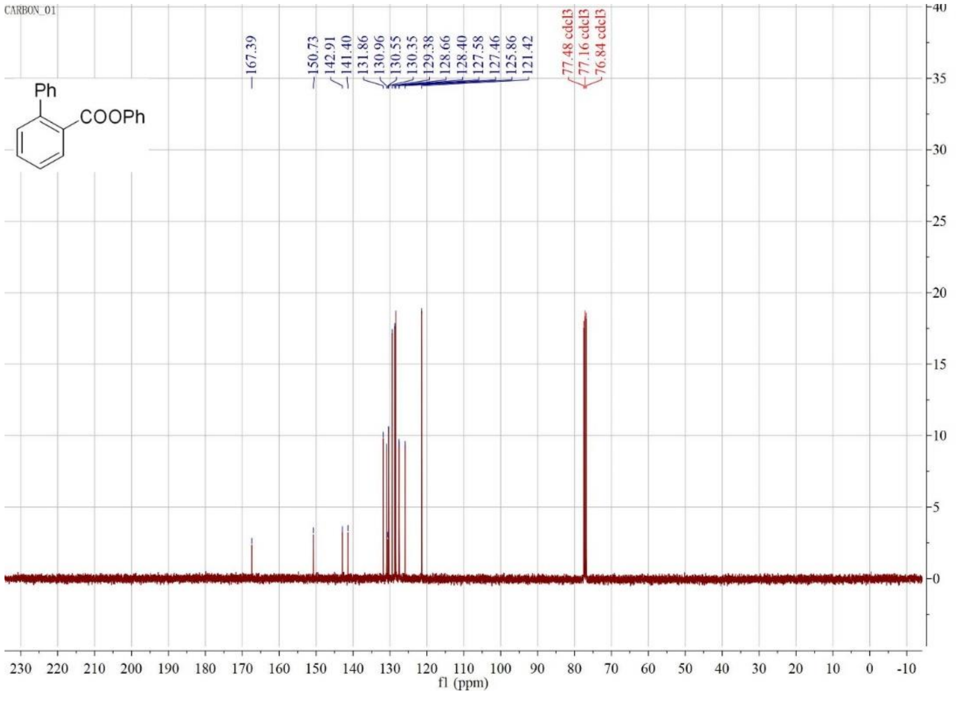

^{1}H NMR spectra of **4f**:

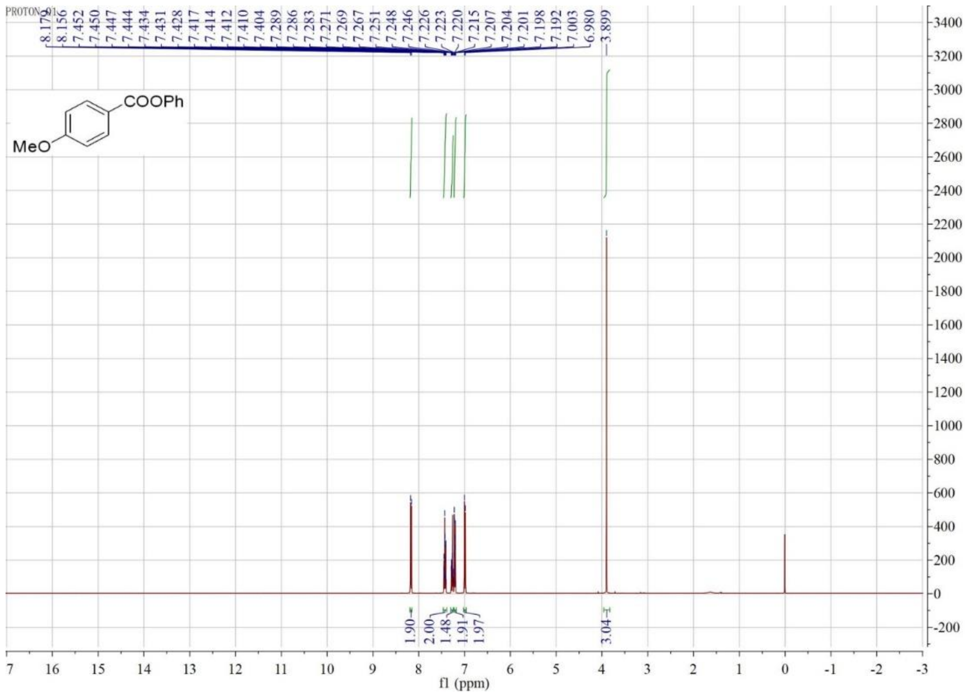

^{13}C NMR spectra of **4f**:

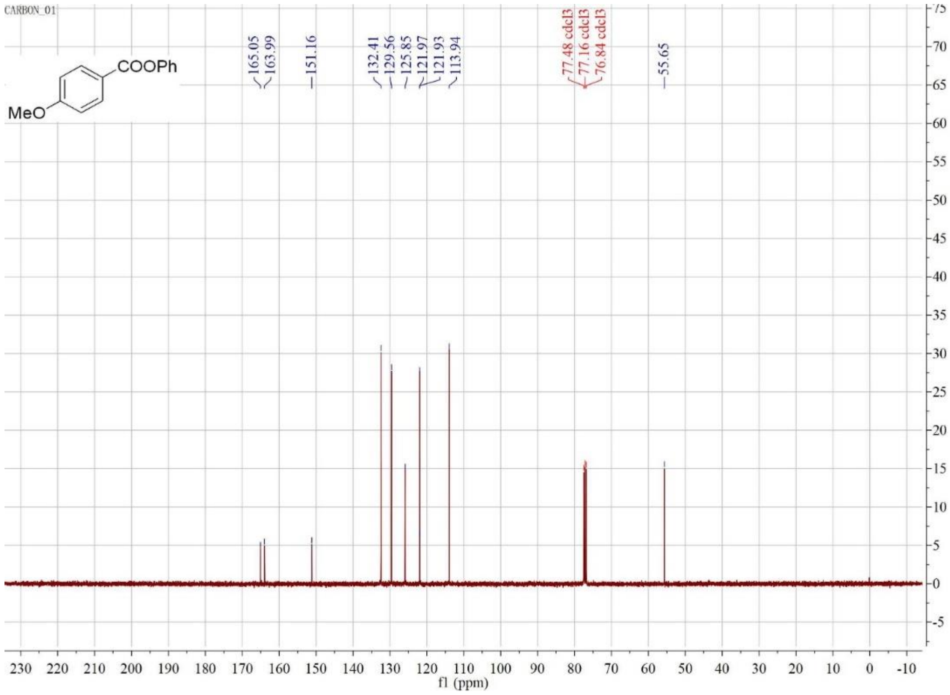

^{1}H NMR spectra of **4g**:

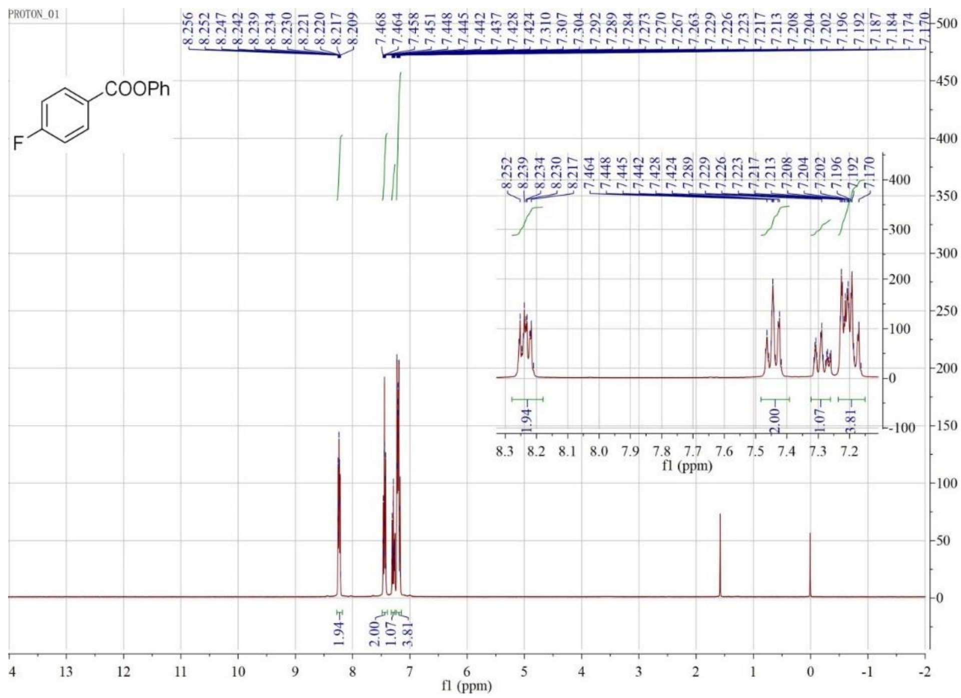

^{13}C NMR spectra of **4g**:

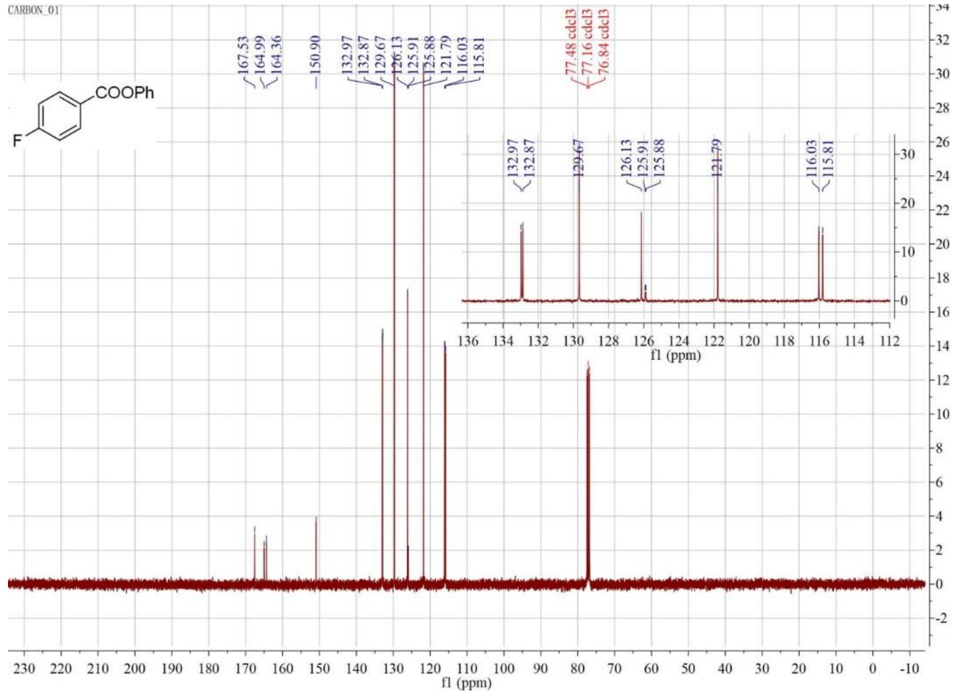

^{19}F NMR spectra of **4g**:

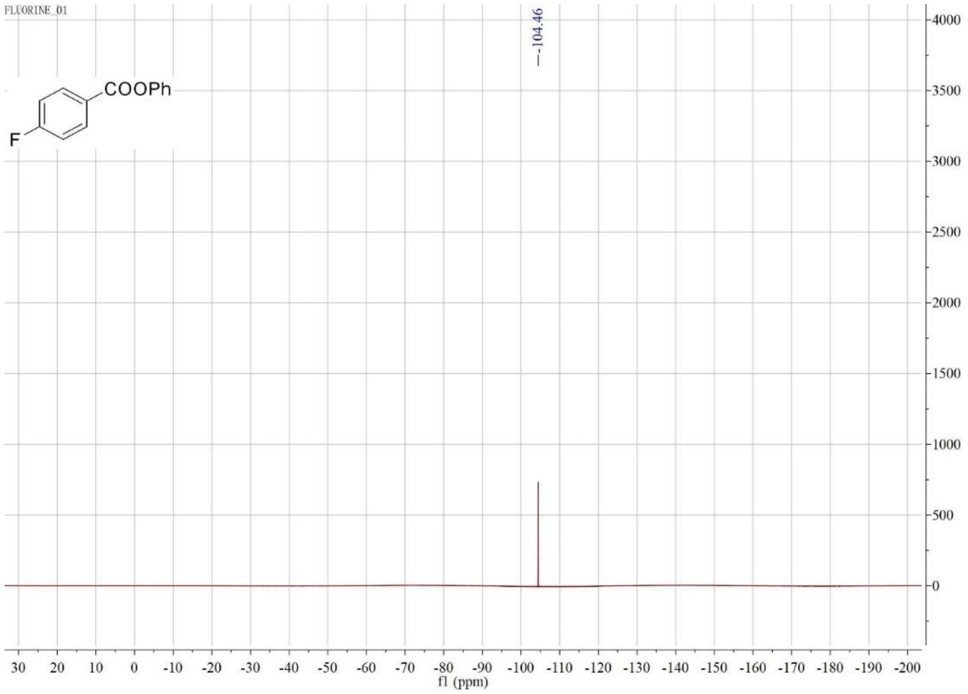

^{1}H NMR spectra of **4h**:

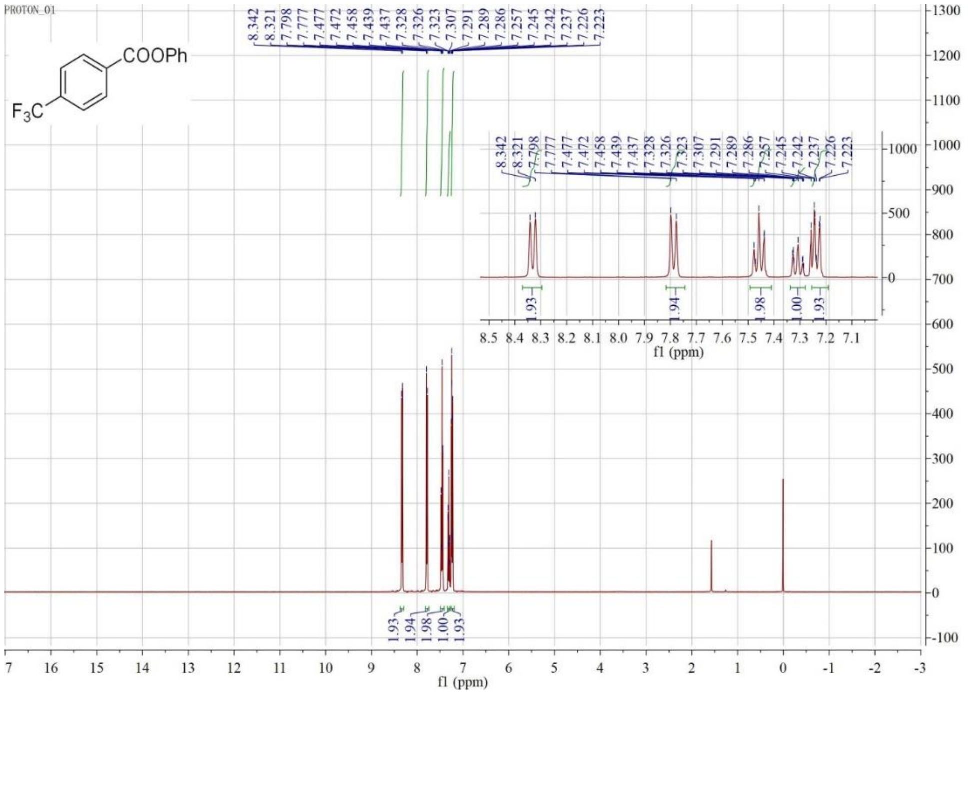

^{13}C NMR spectra of **4h**:

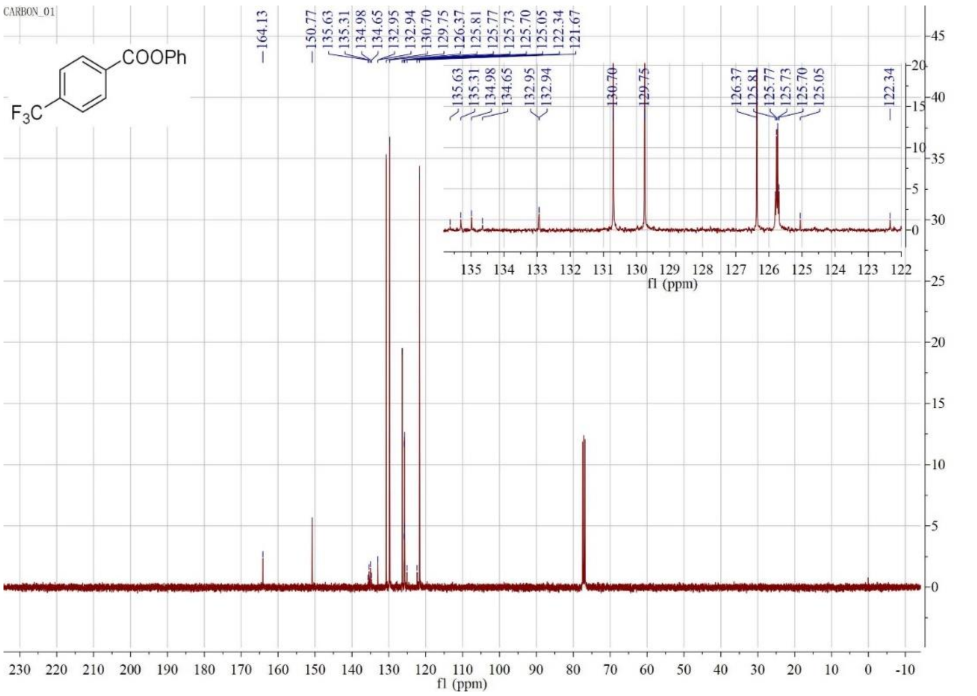

^{19}F NMR spectra of **4h**:

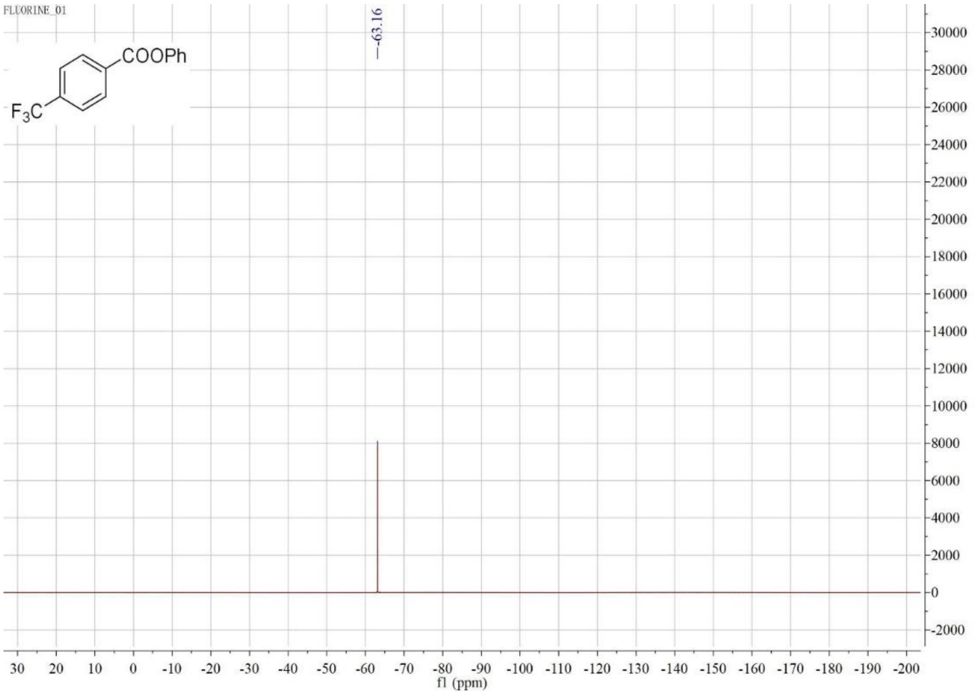

^{1}H NMR spectra of **4q**:

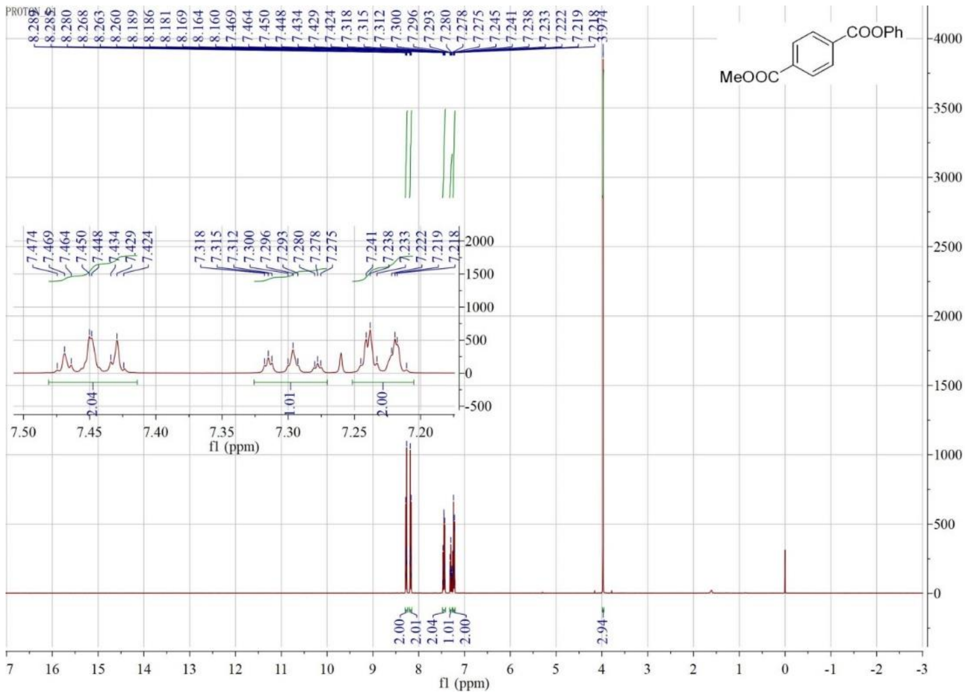

^{13}C NMR spectra of **4q**:

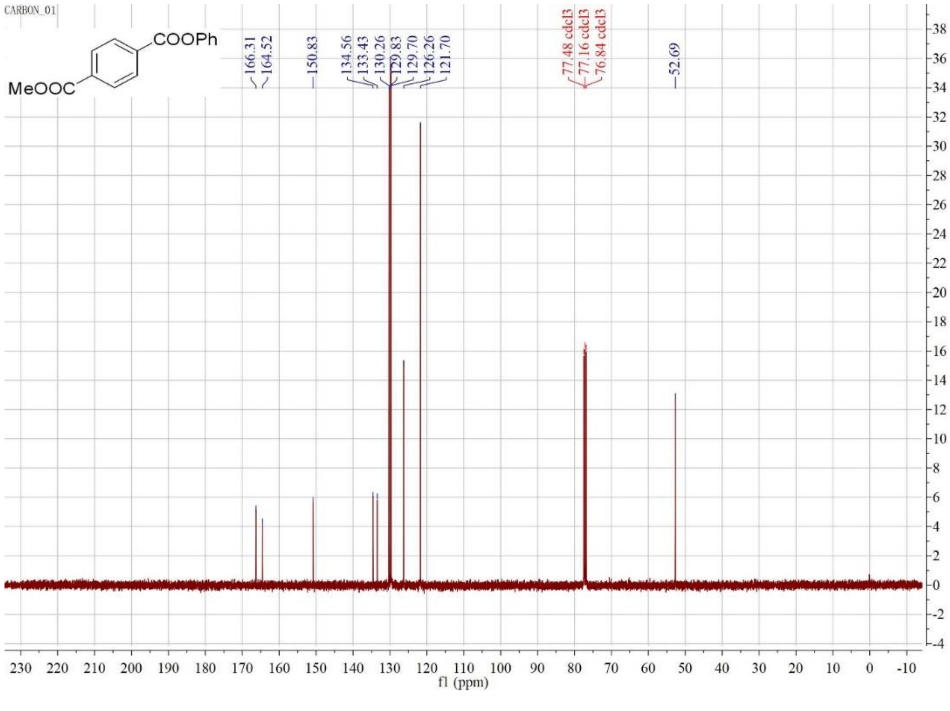

^{1}H NMR spectra of **4r**:

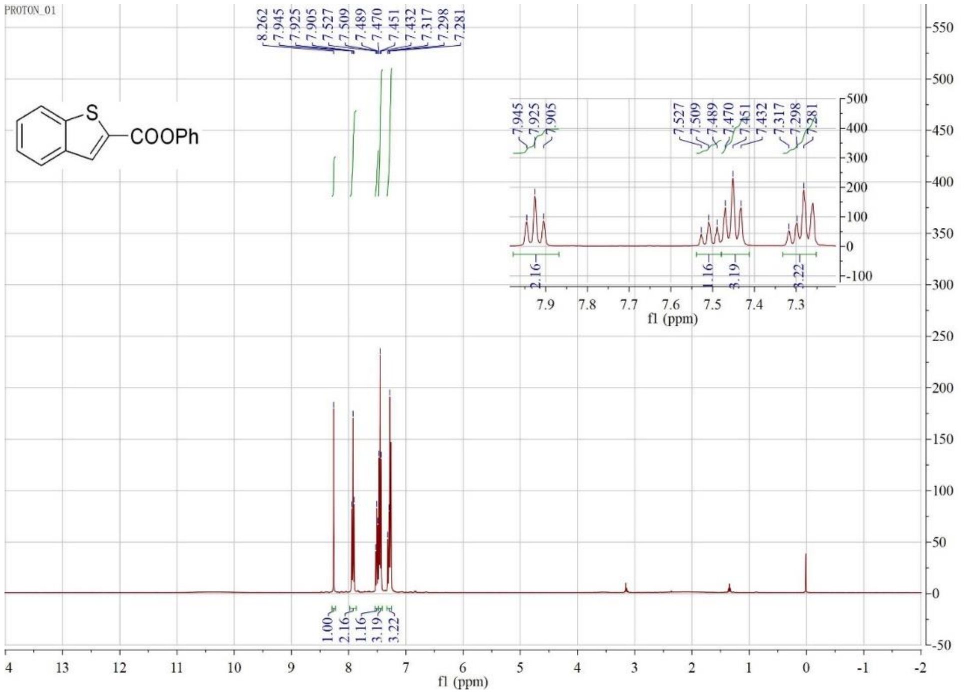

^{13}C NMR spectra of **4r**:

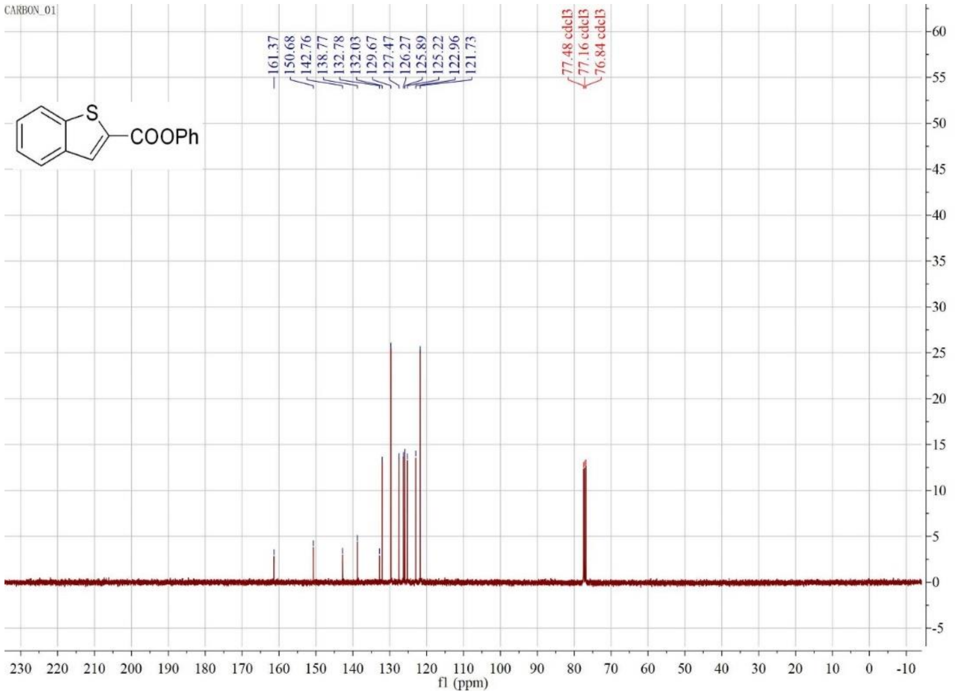

^{1}H NMR spectra of **4s**:

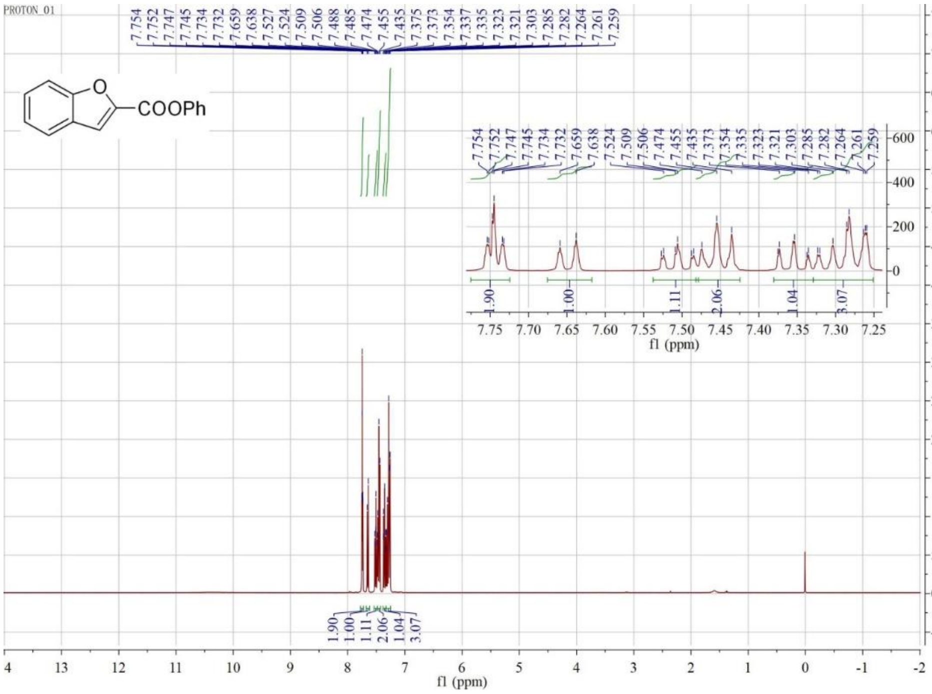

^{13}C NMR spectra of **4s**:

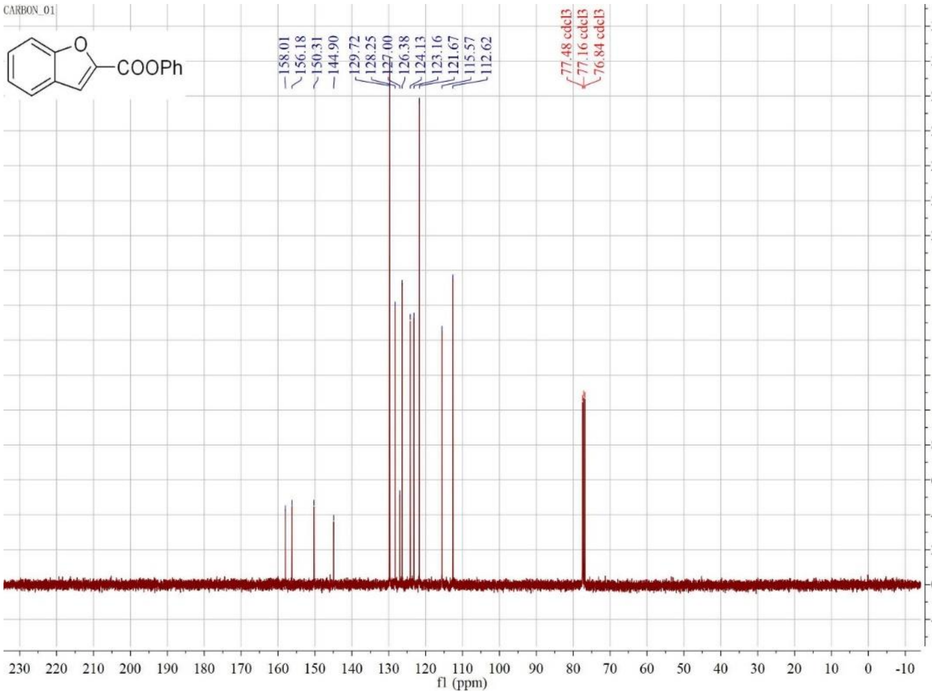

^{1}H NMR spectra of **4t**:

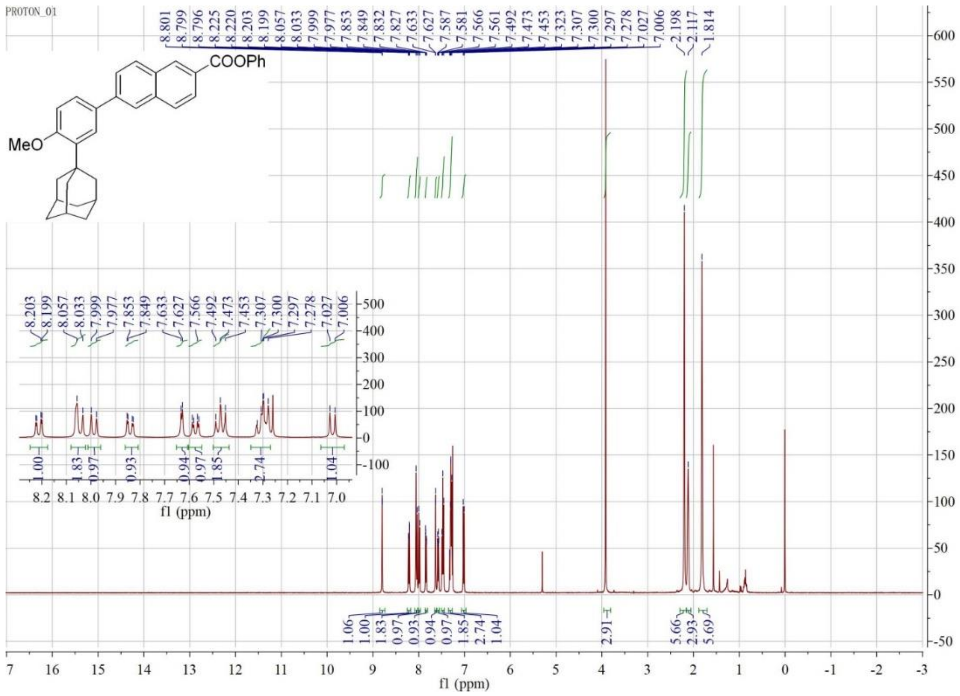

^{13}C NMR spectra of **4t**:

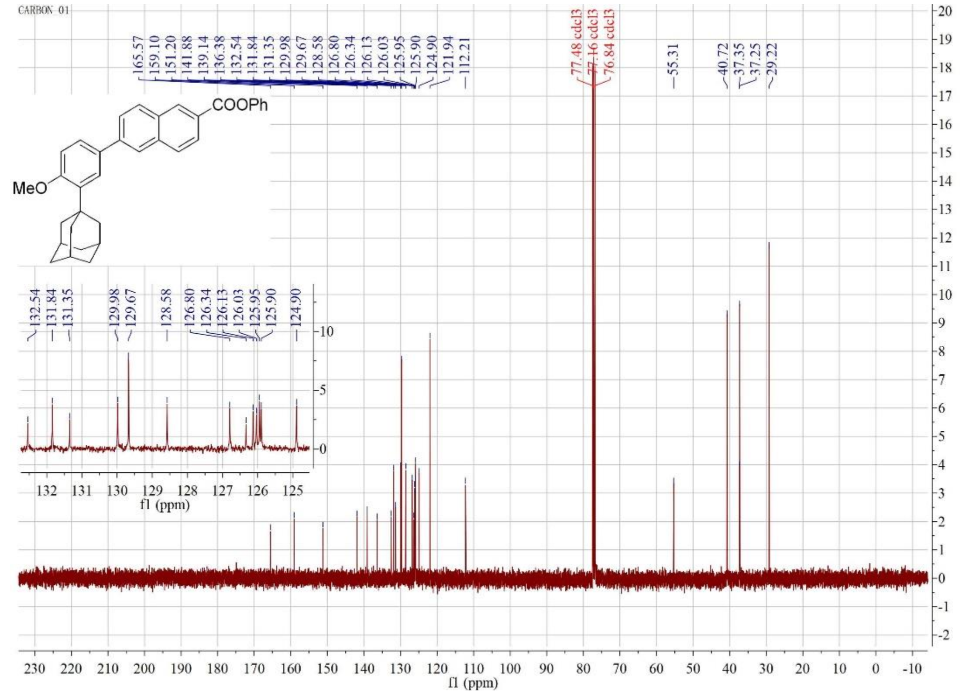

^{1}H NMR spectra of **9**:

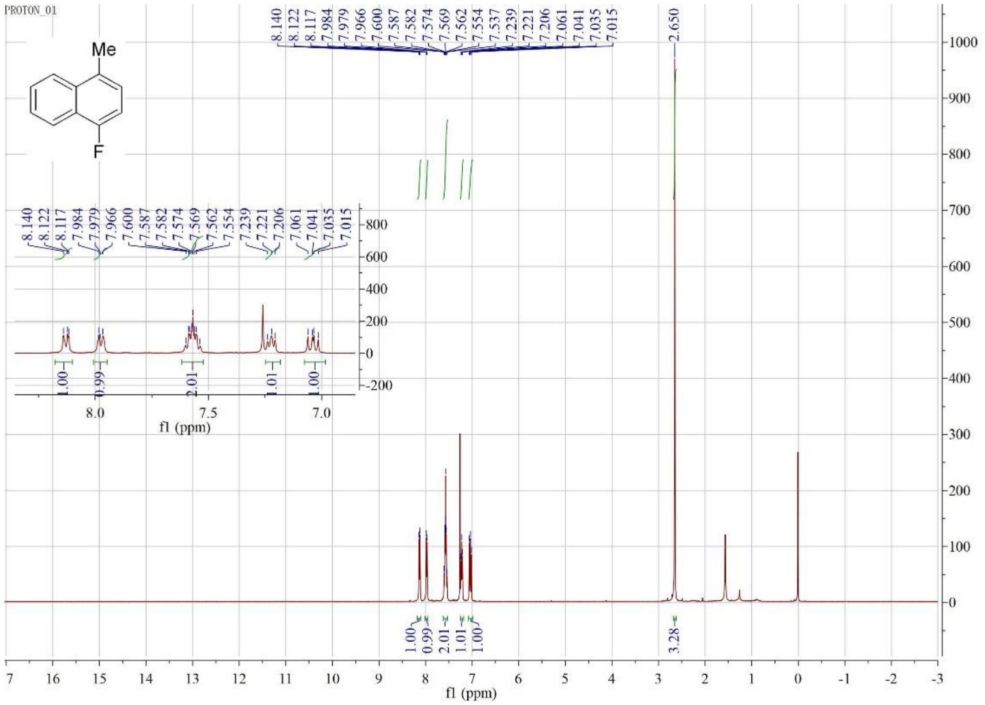

^{13}C NMR spectra of **9**:

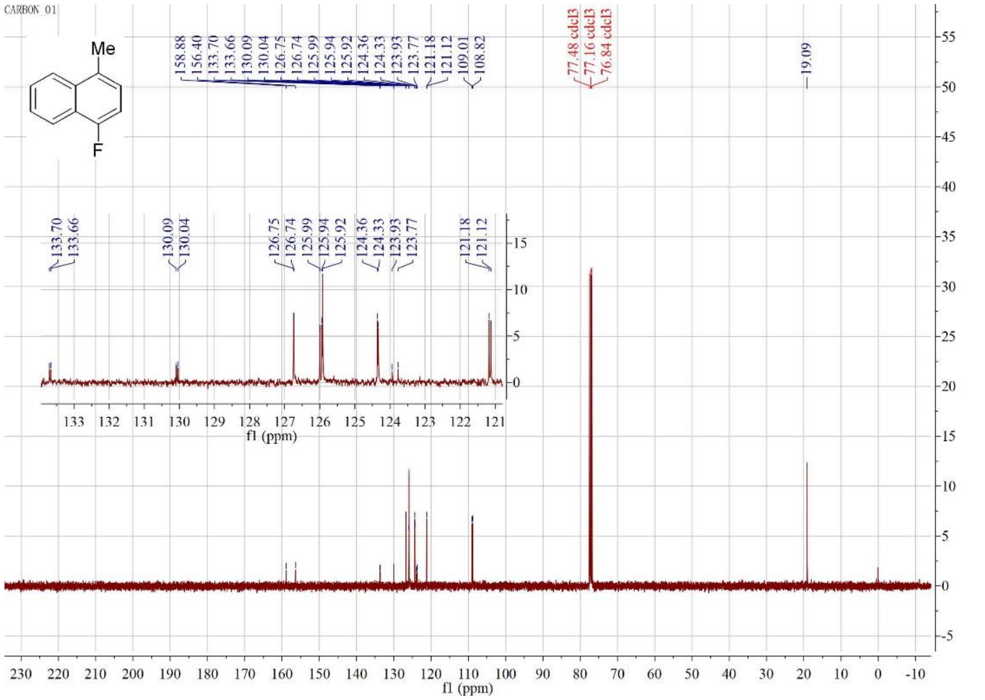

^{19}F NMR spectra of **9**:

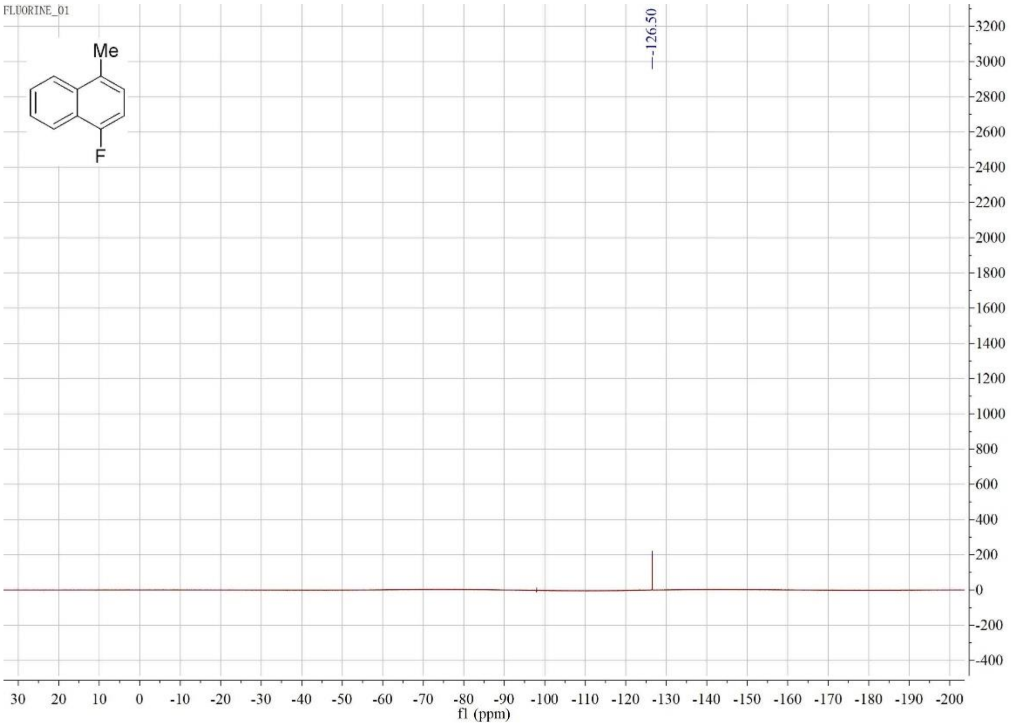

^{1}H NMR spectra of **10**:

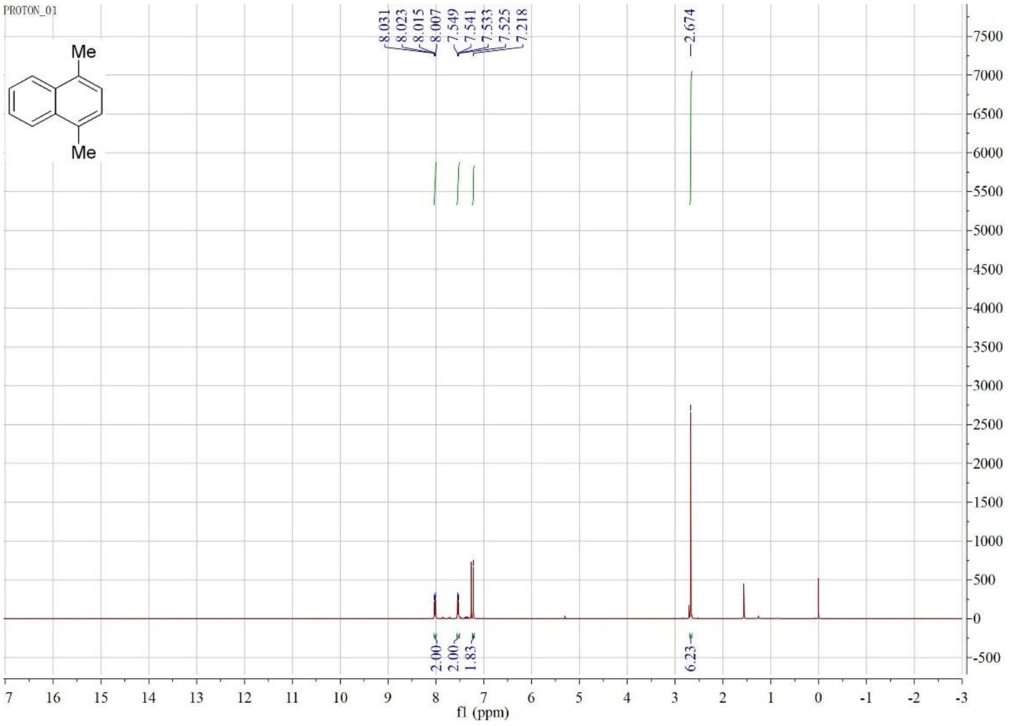

^{13}C NMR spectra of **10**:

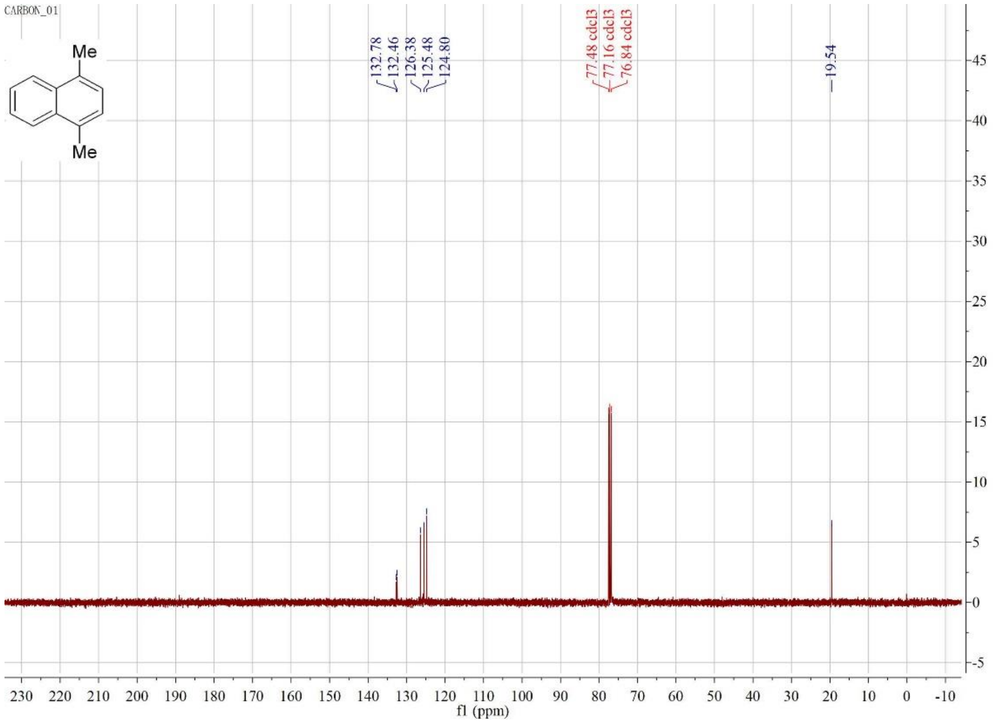

^{1}H NMR spectra of **12**:

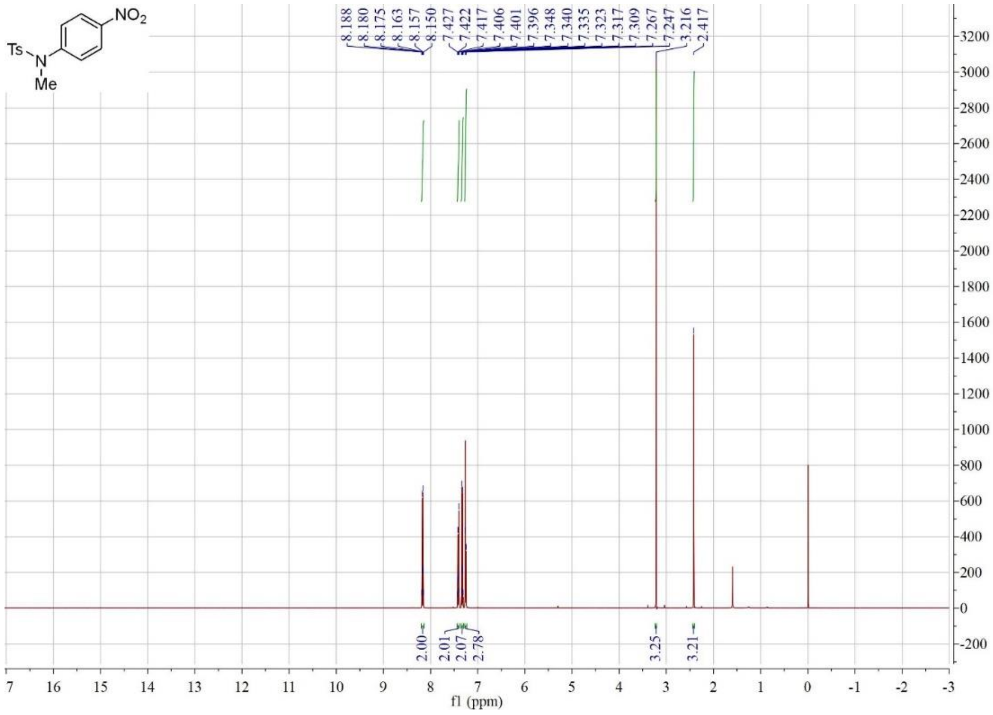

^{13}C NMR spectra of **12**:

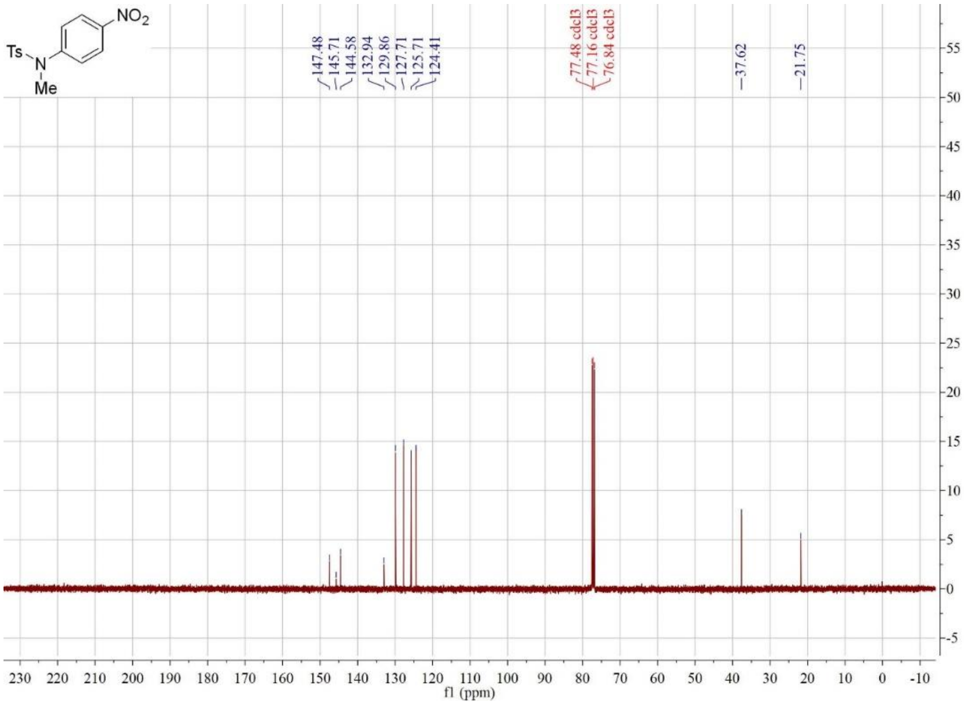

^{1}H NMR spectra of **13**:

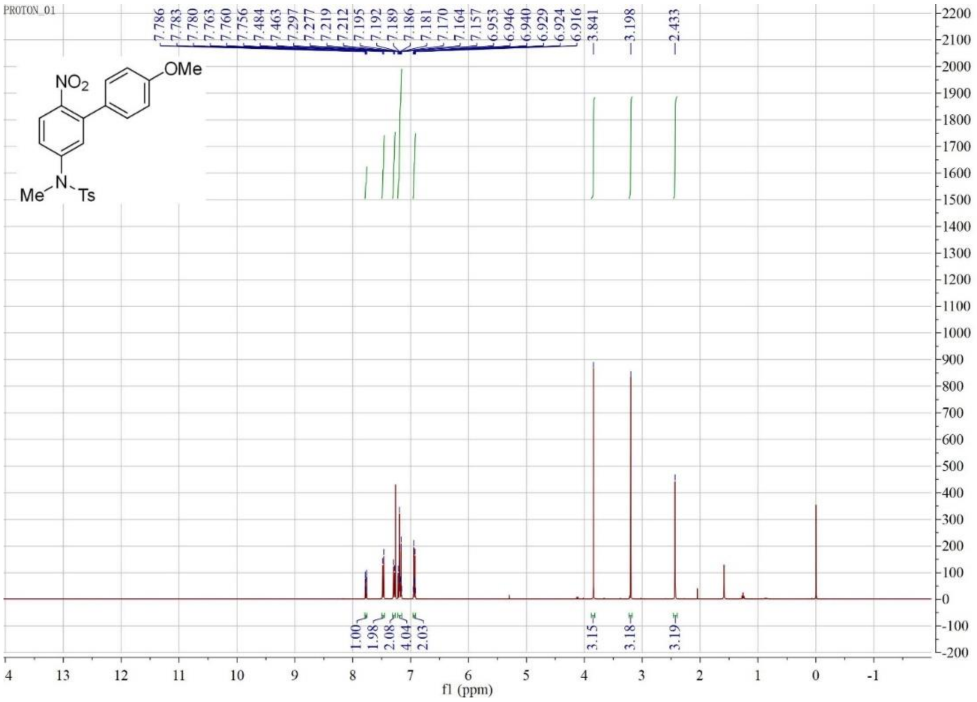

^{13}C NMR spectra of **13**:

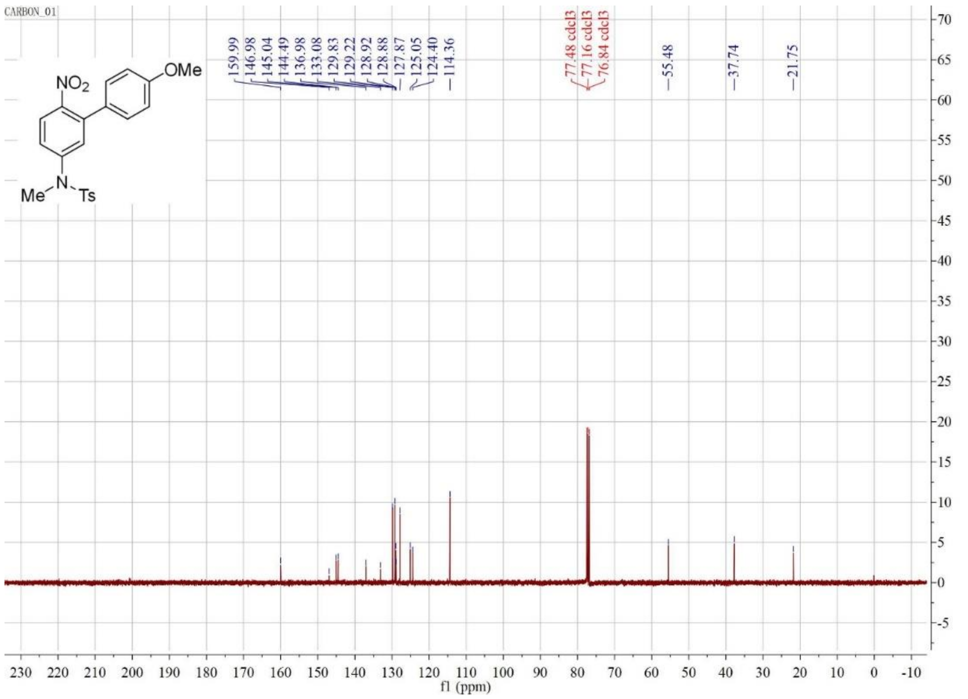

^{1}H NMR spectra of **14**:

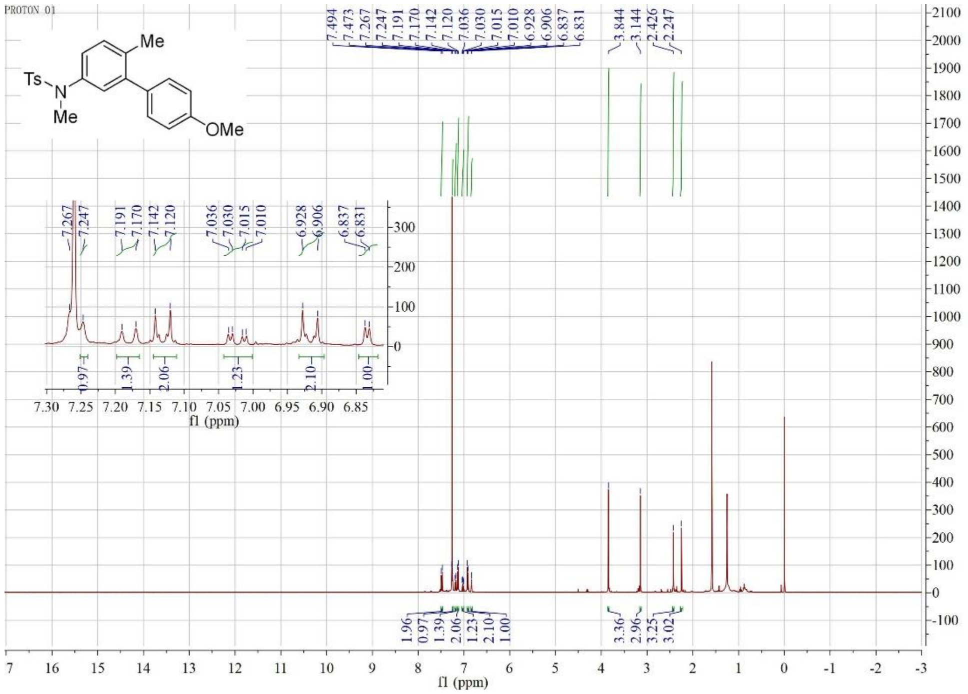

^{13}C NMR spectra of **14**:

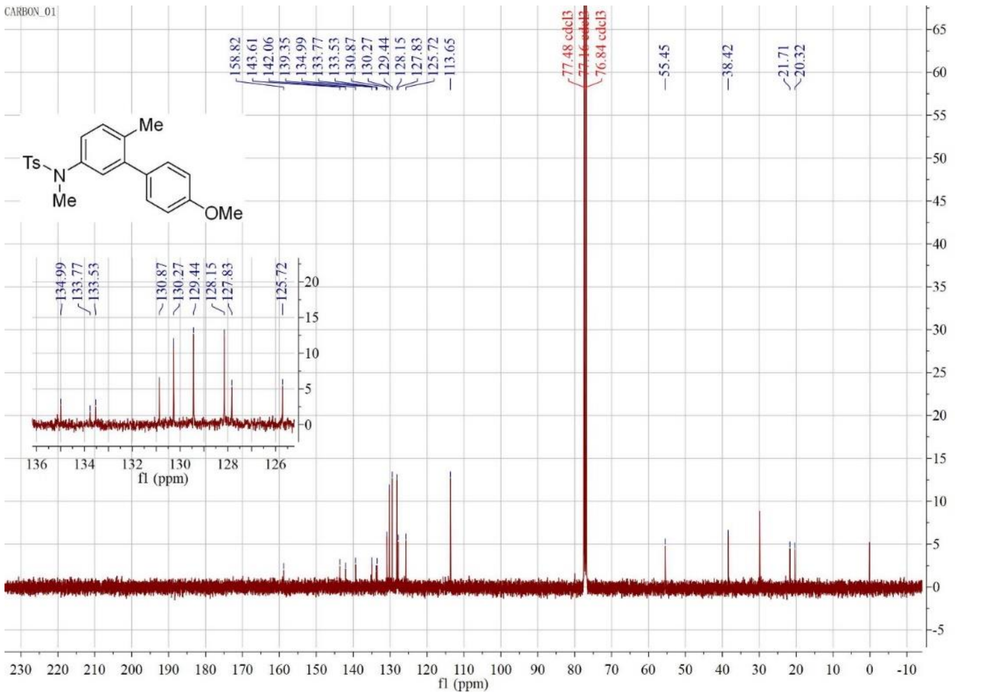

^{1}H NMR spectra of **15**:

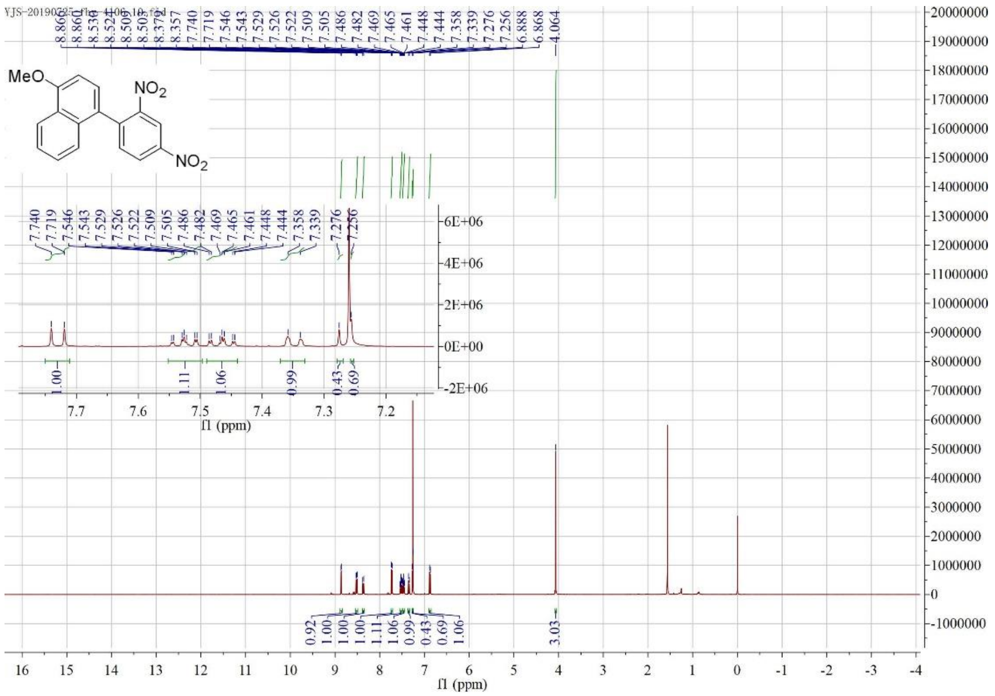

^{13}C NMR spectra of **15**:

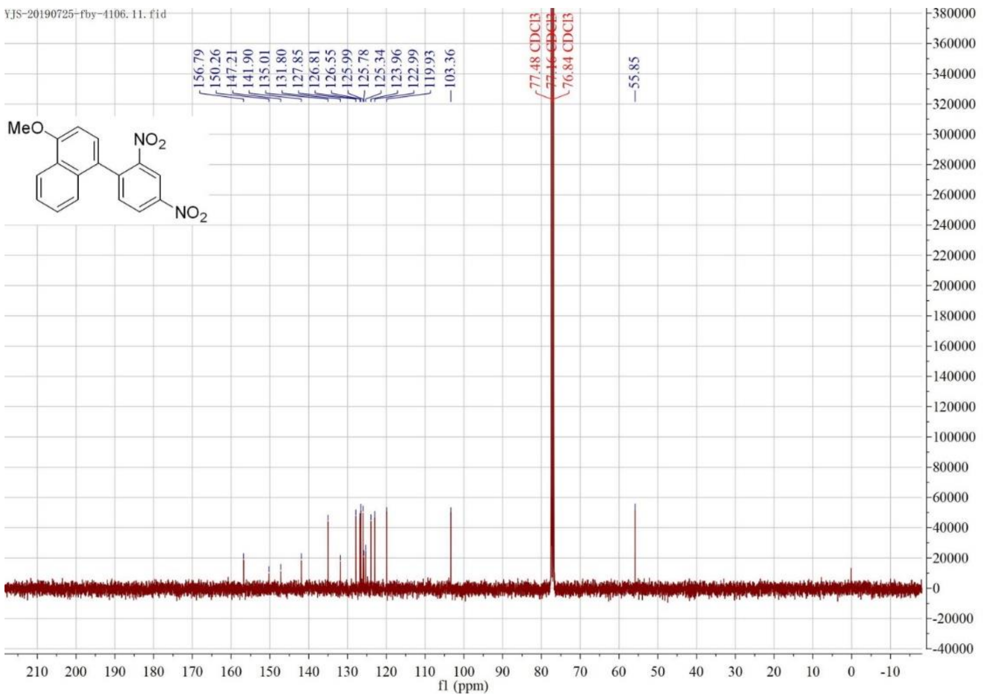

^{1}H NMR spectra of **16**:

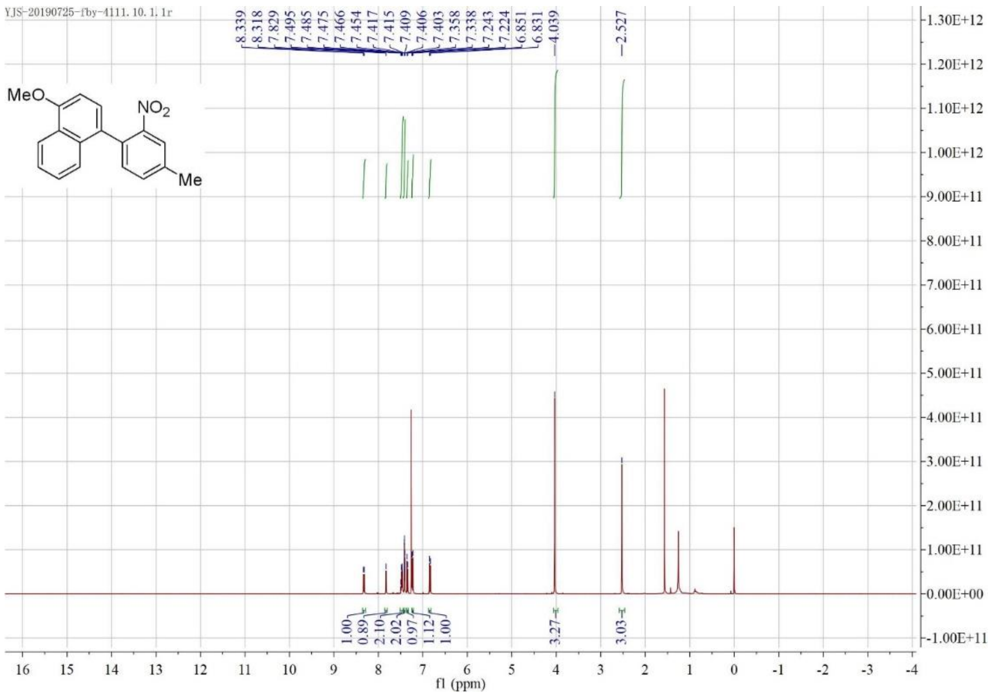

^{13}C NMR spectra of **16**:

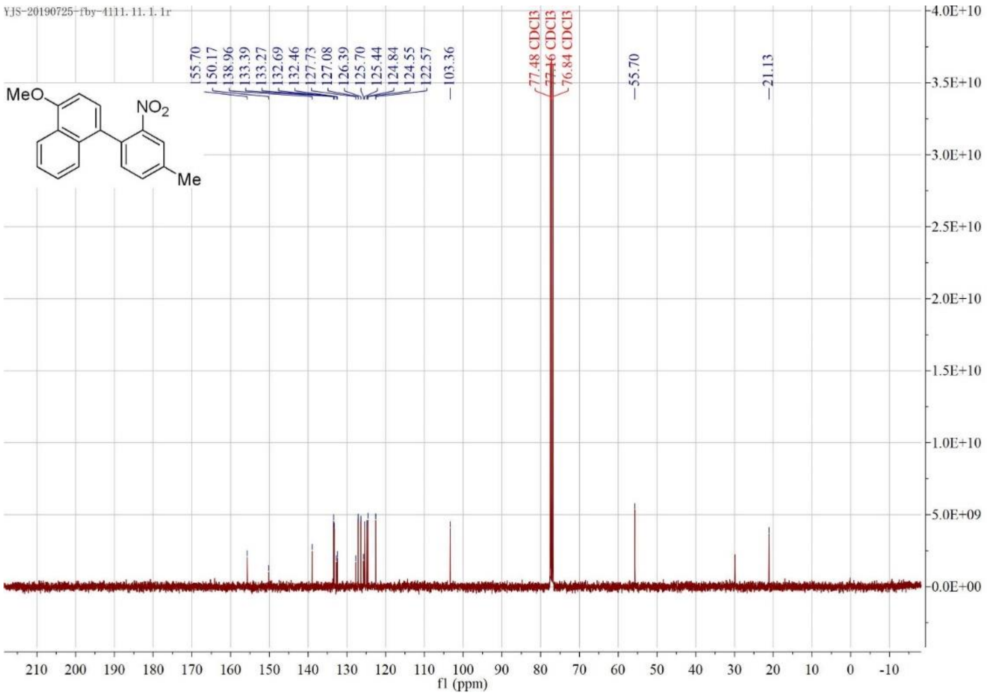

^{1}H NMR spectra of **17**:

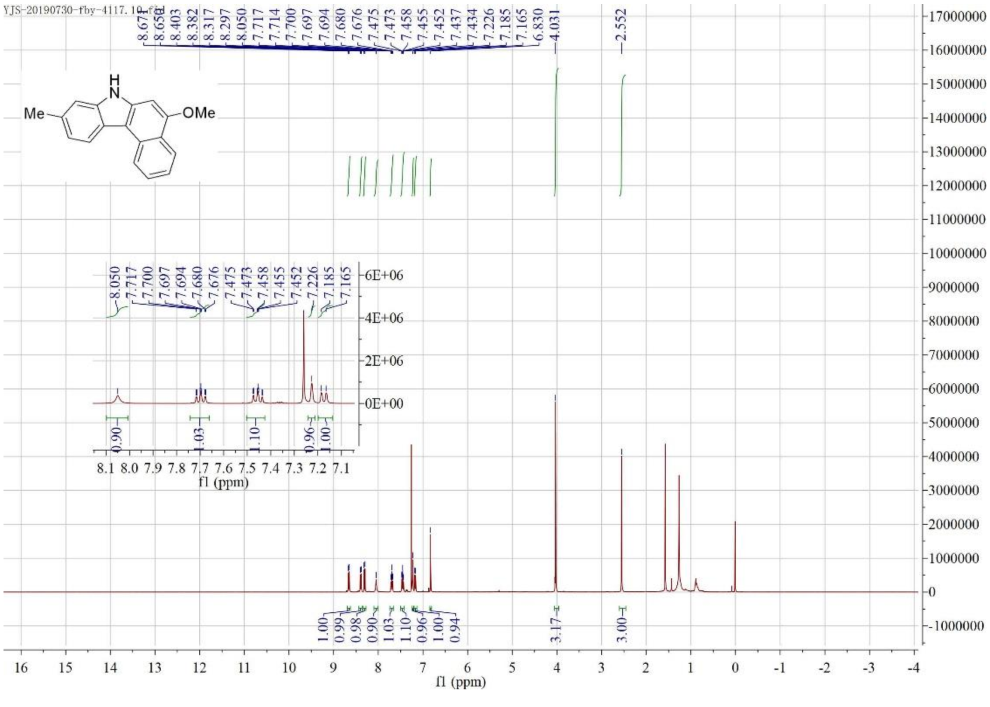

^{13}C NMR spectra of **17**:

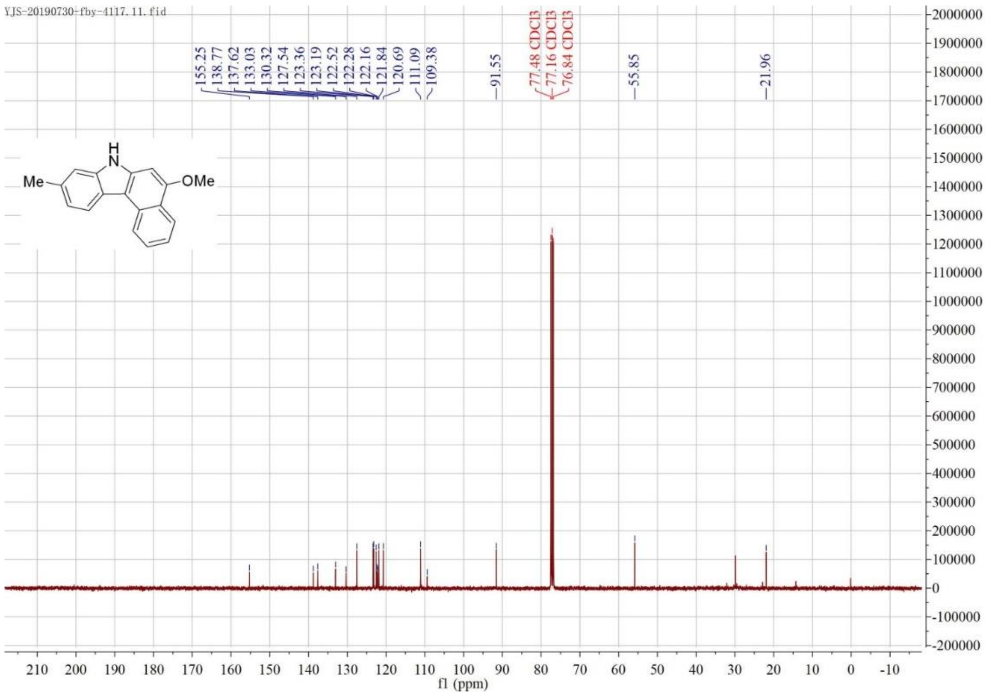

Supplement: SC-011-D0SC01641A-s001 [file SC-011-D0SC01641A-s001.pdf]
